# Transcriptomal profiling of bovine ovarian granulosa and theca interna cells in primary culture in comparison with their *in vivo* counterparts

**DOI:** 10.1371/journal.pone.0173391

**Published:** 2017-03-10

**Authors:** Nicholas Hatzirodos, Claire Glister, Katja Hummitzsch, Helen F. Irving-Rodgers, Philip G. Knight, Raymond J. Rodgers

**Affiliations:** 1 Discipline of Obstetrics and Gynaecology, School of Medicine, Robinson Research Institute, University of Adelaide, SA, Australia; 2 School of Biological Sciences, University of Reading, Hopkins Building, Reading, Whiteknights, United Kingdom; Baylor College of Medicine, UNITED STATES

## Abstract

*In vitro* culture of ovarian granulosa cells and theca cells has been very important for our understanding of their function and regulation. One of the most eagerly sought attributes of cell culture is the use of chemically-defined conditions. However, even under such *in vitro* conditions cell behaviour could differ from the *in vivo* situation because of differences in oxygen tension, nutrients, adhesion matrix and other factors. To examine this further we compared the transcriptomes of both granulosa cells and cells from the theca interna that were cultured in what are arguably the best *in vitro* conditions for maintaining the ‘follicular’ phenotypes of both tissue types, as displayed by their respective freshly-isolated counterparts. The array data analysed are from recently published data and use the same sizes of bovine follicles (small antral 3–6 mm) and the same Affymetrix arrays. We conducted analysis using Partek, Ingenuity Pathway Analysis and GOEAST. Principal Component Analysis (PCA) and hierarchical clustering clearly separated the *in vivo* from the *in vitro* groups for both cells types and transcriptomes were more homogeneous upon culture. In both cell cultures behaviours associated with cell adhesion, migration and interaction with matrix or substrate were more abundant. However, the pathways involved generally differed between the two cell types. With the thecal cultures a gene expression signature of an immune response was more abundant, probably by leukocytes amongst the cells cultured from the theca interna. These results indicate differences between *in vivo* and *in vitro* that should be considered when interpreting *in vitro* data.

## Background

In ovaries oocytes develop within follicles which at the initial primordial stage are composed of an inactive oocyte surrounded by a quiescent population of epithelial granulosa cells. A number of primordial follicles are activated daily and subsequently the granulosa cells begin to divide and, over a period of months in bovine ovaries, increase to about 50 million cells [1]. At the same time as the follicle expands it develops an antral cavity, filled with follicular fluid. Growth of follicles is important for expanding the number of granulosa cells to ensure that enough of the steroid hormone oestradiol is secreted to control and regulate the reproductive cycle. At about the time an antrum forms, the stroma surrounding the membrana granulosa specialises into the theca interna and externa layers. Specialised cells in the vascularised theca interna are steroidogenic and secrete androgens, such as androstenedione and testosterone, which are converted by granulosa cells into oestradiol. Differentiation of the theca interna is therefore integral for oestrogen synthesis.

During early stages of growth, the granulosa cells express receptors for follicle-stimulating hormone (FSH), which is secreted from the anterior pituitary gland and stimulates follicle growth. Very late in follicular development the granulosa cells also express receptors for luteinising hormone (LH) and large follicles which have granulosa cells expressing these receptors are capable of ovulating in response to a surge release of LH from the anterior pituitary. The steroidogenic cells of the theca, on the other hand, express LH receptors from an early stage and respond to LH by synthesising androgens. Both the granulosa cells and the theca interna cells are key somatic cell types whose function and regulation are pivotal to follicle development, steroidogenesis and female fertility.

The development of *in vitro* culture systems for both granulosa cells and theca cells has been very important for our understanding of their function and regulation within the context of follicle development and ovarian steroidogenesis. Earlier studies utilising culture media supplemented with fetal calf serum [2] showed that granulosa cells have a propensity to differentiate terminally into luteal cells, as occurs upon ovulation *in vivo*. Anchorage-independent culture was used to examine the stem cell population of granulosa cells [3,4] and this was followed by a serum free system [5], in which granulosa cells retained the ability to produce oestradiol. *In vivo*, this feature of granulosa cells is seen during the final stages of antral follicle maturation and particularly in large follicles approaching ovulatory size [6]. An important property of thecal cells in culture is their ability to produce androgens and retain responsiveness to LH [7]. These properties are lost when cells are cultured in serum-containing media and the cells undergo changes characteristic of luteinisation.

One of the most eagerly sought attributes of cell culture is the use of chemically-defined conditions where the use of fetal calf serum or other biological reagents that might vary from batch to batch is avoided. However, even such *in vitro* conditions could differ markedly from *in vivo* conditions because of differences in oxygen tension, nutrients and a myriad of other factors. *In vitro*, cell behaviour could also be affected by the sporadic changes of cell culture media that is likely to produce acute temporal fluctuations in the concentrations of nutrients and metabolites during culture. Additionally, interactions with cell culture plastic and altered cell-cell interactions could occur *in vitro*. Even just the process of harvesting cells and transferring them into culture could alter their phenotype *in vitro*. Therefore, it would be expected that differences exist between the *in vivo* and *in vitro* conditions. However, most *in vitro* studies on ovarian granulosa and theca interna cells have been confined to examination of a limited set of targeted parameters like steroidogenesis and expression of known receptors and growth factors.

To examine the effects of culture further we compared the transcriptomes of both granulosa cells and cells from the theca interna that were cultured in what are arguably regarded as the most physiologically relevant culture conditions for maintaining ‘follicular’ (i.e. non-luteinized) phenotypes *in vitro* of both cell types [5,8–10] comparable with those of their respective freshly-isolated (*in vivo*) counterparts. The microarray data are from recently published reports from our groups in which comparisons were made within *in vivo* [granulosa cells [11], thecal cells [12]] and within *in vitro* [granulosa cells [13], theca interna [14]] conditions but not between *in vivo* and *in vitro* conditions. These previous studies whilst conducted in different locations used the same sizes of bovine follicles (small antral 3–6 mm) and the same Affymetrix arrays. During culture, steroid hormone production was monitored and confirmed that both the granulosa and thecal cells maintained their follicular phenotypes [13,14].

## Materials and methods

### Tissues and cells, RNA extraction and array hybridisation

*In vivo* studies: Granulosa cells and intact theca interna were isolated from two different groups of individual small healthy follicles (n = 10 each group, 3–5 mm in diameter) obtained from an abattoir according to previously described methods [15,16]. The health status of the follicles was confirmed by histological examination of a portion of the follicle wall based on the number of apoptotic figures observed in the membrana granulosa [17,18]. RNA was extracted from granulosa cells and theca interna of each follicle preparation and two μg of RNA per sample was processed for hybridisation to a Bovine Affymetrix Genome Array (Australian Genomics Research Facility, Parkville and ACRF Cancer Genomics facility, Adelaide) whereby ten arrays each of either granulosa cells or theca interna were available for analysis as was previously published by our groups [11,12]. The CEL files can be obtained at GSE39589 and GSE49505 for granulosa and theca data, respectively.

*In vitro* studies: Two separate experiments utilised granulosa cells and cells of the theca interna each isolated from bovine ovarian follicles 4–6 mm in diameter obtained from an abattoir as previously described [8–10]. For granulosa cell isolation [10] medium-sized follicles (4- to 6-mm diameter) lacking obvious signs of atresia were removed, follicular fluid was aspirated, a slit was made in the follicle wall and the granulosa cell layer was gently disrupted with the aid of a plastic inoculation loop. Double-distilled water (10 ml) was added and the cells were agitated for 10 secs to lyse any red blood cells present; isotonicity was quickly restored by the addition of 10 ml of 3× concentrated PBS. For thecal cell isolation [9] follicles were isolated and granulosa cells removed. Follicle halves were then shaken vigorously to remove any remaining granulosa cells, and the medium was changed three times. Follicle halves were examined under a dissecting microscope, and the theca interna layer was gently peeled away from the basement membrane. Pooled theca layers were incubated with collagenase (type IV, 1 mg/ml; Sigma Ltd., Poole, UK) and trypsin inhibitor (100 μg/ml; Sigma). After 30 min, the cell layers were triturated with a Pasteur pipette and returned to the water bath for another 15 min. Finally, any remaining undigested material was allowed to settle, and the resulting theca-cell-rich supernatant decanted. In each of 4 independent replicate cultures, pooled granulosa or theca cells from approximately 50 follicles were seeded at 5 x 10^5^/ml in 24 well plates and incubated for six days with hormone /growth factor treatments on days 4 to 6 of culture in McCoys 5A medium supplemented with antibiotics, 0.1% bovine serum albumin and other growth supplements as detailed previously [13,14]. The granulosa cells were cultured under four conditions: FSH only (0.33 ng/ml), tumour necrosis factor α (TNFα) only (10 ng/ml), FSH plus TNFα or no treatment (control) [13], but only control and FSH treated cells were analysed here. The theca cells were also subjected to four treatments: LH only (160 pg/ml), bone morphogenic protein-6 (BMP-6) only (10 ng/ml), LH plus BMP-6 or control [14], but only control and LH treated cells were analysed here. In both experiments the production of steroid hormones was measured to ensure the cells retained their follicular phenotype [13,14]. Each independent experiment used n = 4 wells for each condition and two μg of RNA from pooled extracts was used to hybridise to a Bovine Affymetrix Genome Array for analysis of global gene expression (Almac Diagnostics Limited, Craigavon, UK) forming the basis of recently published studies [13,14]. The CEL files can be obtained at GSE42535 and GSE44704 for granulosa and theca data, respectively.

### Array analyses

Affymetrix CEL file data were pre-processed in Partek Genomics Suite (version beta 6.6; St Louis, Missouri, USA) using RMA background summarisation, with quantile normalisation and log base 2 transformation and mean probe set summarisation with adjustment for GC content. All arrays passed the spike-in hybridisation quality controls as conducted previously [11].

### Clustering and statistical analyses

Partek Genomics Suite was used for clustering and statistical analysis of the array data. Initially, PCA and hierarchical clustering were conducted separately on granulosa (n = 17) and thecal cell arrays (n = 18) according to parameters previously published [11,15]. The hormone-treated (either FSH or LH, for granulosa and thecal cell, respectively) and control cultured arrays were analysed collectively as the single group *in vitro* due to their similar transcriptional profiles [13,14]. Comparison of the *in vitro* and *in vivo* groups for both the granulosa cell and theca interna arrays was performed by one-way ANOVA with followed by Benjamini-Hochberg false discovery rate corrections for multiple testing.

### Ingenuity Pathway Analyses (IPA) and GOEAST snalyses

Canonical pathway analysis was conducted in IPA and Gene Ontology enrichment analysis in GOEAST (Gene Ontology Enrichment Analysis Software Toolkit) as previously described and using statistically significantly differentially-expressed genes identified by ANOVA with followed by Benjamini-Hochberg false discovery rate corrections for multiple testing [11]. Regulator effect network analysis was also conducted in IPA whereby possible upstream regulators determined from the Ingenuity knowledge base from our differentially expressed data sets were matched with predicted downstream processes using Fishers exact *t*-test and an algorithm which computes the consistency of these interactions [19]. Only the highest ranked networks are presented for each analysed data set.

## Results

### Behaviour of granulosa cells and theca interna cells during in vitro culture

**Fig 1:**
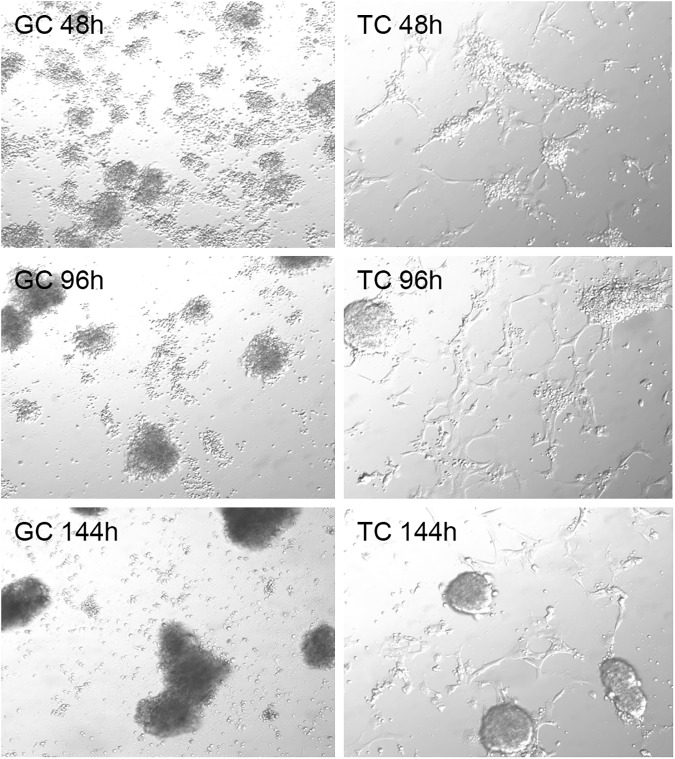
Appearance of Granulosa Cells (GC) and Thecal Cells (TC) cultured under serum-free conditions for 48, 96 and 144 hours.

Dispersed granulosa cells and theca interna cells re-aggregated into multicellular clumps during the course of the 6-day culture periods (Fig 1) indicating cell-cell or cell-extracellular matrix interactions as seen previously [20].

### Comparisons of in vivo and in vitro granulosa cell arrays

The expression of the housekeeping genes was similar between the *in vivo* and *in vitro* situations for granulosa cells with the mean probe intensity of the housekeeping genes GAPDH, ACTB, PPIA and MRPL32 on the *in vitro* chips being 1.229, 1.041, 0.875 and 1.385-fold of that of the *in vivo* chips, respectively.

**Fig 2:**
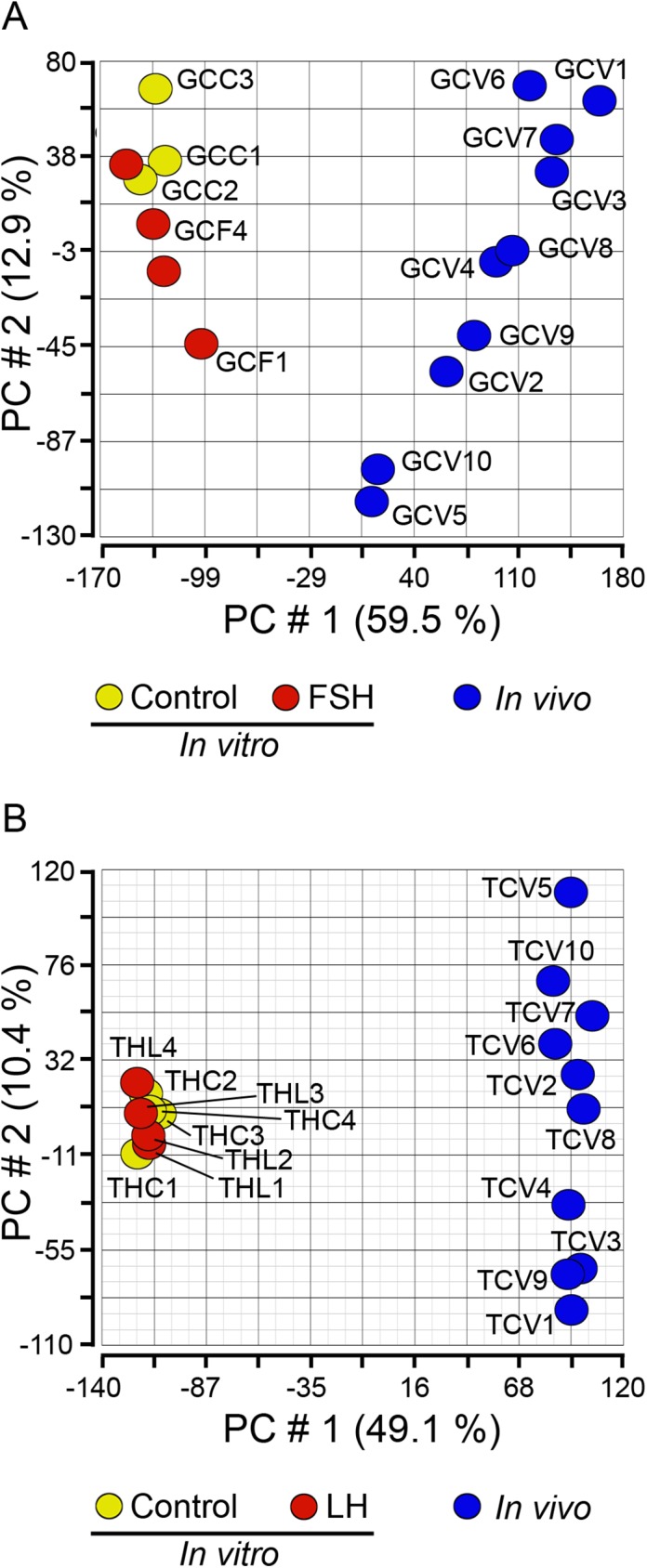
**Unsupervised PCA of granulosa cell (A) and theca interna (B) arrays**. The graphs are scatter plots of the values for the first (X) and second (Y) principal components based on the correlation matrix of the total normalised array intensity data. The numbering of each sample enables the samples in the figure to be identified in S1 and S2 Figs. Abbreviations are GC (granulosa cell), TH (theca interna), CC (cultured under control conditions), CF (cultured and FSH treated), CL (cultured and LH treated) and V (*in vivo* freshly isolated cells).

Principal component analysis (PCA) (Fig 2A) and hierarchical clustering (S1 Fig) were applied to *in vitro* and *in vivo* granulosa cell array data. These two methods separated the two groups from each other with a greater spread evident in the *in vivo* group. The differentially expressed genes are listed in Tables 1 and 2. Genes that are known to be specifically highly expressed in granulosa cells from antral follicles such as *CYP19A1*, *FSHR* and *AMH* were found in lower abundance *in vitro* (Table 1). In contrast, many of the genes which more abundant *in vitro* (Table 2), including *CTGF*, *COL1A2*, *COL5A2*, *DCN* and *BMP2* are related to extracellular matrix production and hence tend to fit a mesenchymal tissue expression profile.

**Table 1:** Top 50 genes that were most differentially up regulated *in vitro* in granulosa cells.

Gene Symbol	Fold-Change	Gene Symbol	Fold-Change
*TNFAIP6*	316.1	S100A12	29.8
*XCL1*	145.2	NUAK1	28.3
*STC1*	102.7	*COL5A2*	27.5
*ANKRD1*	102.1	*BMP2*	26.2
*SFRP2*	97.1	*CLCA3*	26.1
*PLAT*	79.4	*SGK1*	24.9
*CXCL6*	67.1	*CTSH*	24.7
*DCN*	66.7	*GFPT2*	24.7
*STAR*	62.8	*PLK2*	24.3
*PDGFRA*	57.8	*TIMP1*	23.5
*PTX3*	55.1	*CXCL2*	22.8
*IL18*	52.6	*NCAM1*	22.5
*DKK3*	50.1	*AXL*	22.4
*SCG2*	49.0	*GIMAP8 /// LOC100847526*	21.0
*NID2*	49.0	*FN1*	20.9
*CLDN11*	43.4	*CAV1*	20.8
*RND3*	39.1	*ANXA1*	19.3
*THBS2*	38.6	*OLR1*	19.3
*TNFRSF12A*	38.2	*COL3A1*	19.2
*LGALS3*	35.6	*TRIB1*	18.5
*MMP9*	34.9	*IGFBP5*	18.5
*CTGF*	33.2	*MAP1LC3C*	18.4
*COL1A2*	32.8	*NDP*	17.8
*PRKAG2*	31.0	*DCLK1*	17.6
*LUM*	30.5	*POSTN*	17.4

**Table 2:** Top 50 genes that were most differentially down regulated *in vitro* in granulosa cells.

Gene Symbol	Fold-Change	Gene Symbol	Fold-Change
*IHH*	26.8	*LAMC2*	6.8
*CARTPT*	20.0	*NALCN*	6.7
*MEST*	18.9	*CHRDL1*	6.5
*HSD17B1*	15.5	*LTF*	6.4
*ETNK2*	13.0	*PLEK*	6.3
*PTI*	12.8	*SVOPL*	6.2
*GUCA1A*	12.6	*CRISPLD2*	6.0
*PLA2G1B*	12.6	*CA14*	6.0
*GPC3*	12.2	*KIFC1*	6.0
*SLC27A3*	10.0	*STAC3*	6.0
*SEP4*	10.0	*SLC10A2*	5.8
*CYP19A1*	9.3	*NOS2*	5.8
*FSHR*	9.1	*HAUS4*	5.7
*HSPA1A*	9.0	*JAKMIP1*	5.7
*MYH11*	8.8	*PABPN1*	5.5
*ANGPT2*	8.8	*RAC3*	5.5
*SLC22A3*	8.7	*MOB3B*	5.4
*GANAB*	8.6	*AQP1*	5.4
*GYLTL1B*	8.0	*PGF*	5.2
*ABCC8*	7.8	*AOAH*	5.2
*AMH*	7.7	*TOP2A*	5.2
*ACSS1*	7.3	*PKDCC*	5.1
*GPT*	7.2	*IQGAP2*	5.1
*NUP210*	7.2	*EFHD1*	5.1
*CCBL1*	7.0	*SORL1*	5.1

**Fig 3:**
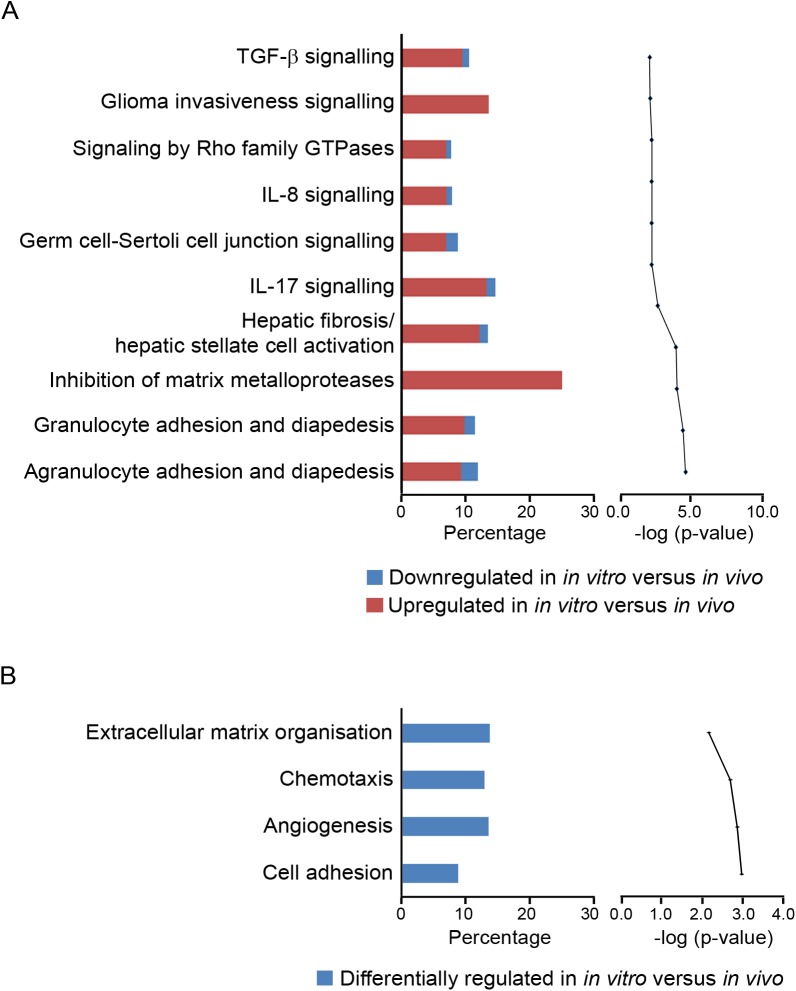
**Top canonical pathways in granulosa cells mapped in IPA (A) and GO terms (B) classified under biological process**. In (A) the bar chart on the left represents the percentage of genes from the data set that map to each canonical pathway showing those which are up regulated (in red) and down regulated (in blue) *in vitro* with respect to *in vivo*. The line chart on the right ranks these pathways derived for the same data set, from the highest to lowest degree of association based on multiple correction testing for the Benjamini-Hochberg False Discovery Rate. In (B) the bar chart on the left represents the percentage of genes from the data set that map to each GO term showing those which are differentially regulated (in blue) *in vitro* with respect to *in vivo*. The line chart on the right ranks these pathways derived for the same data set, from the highest to lowest degree of association using the Benjamini-Yuketeli test for multiple corrections (bottom to top in graphs on right).

**Fig 4:**
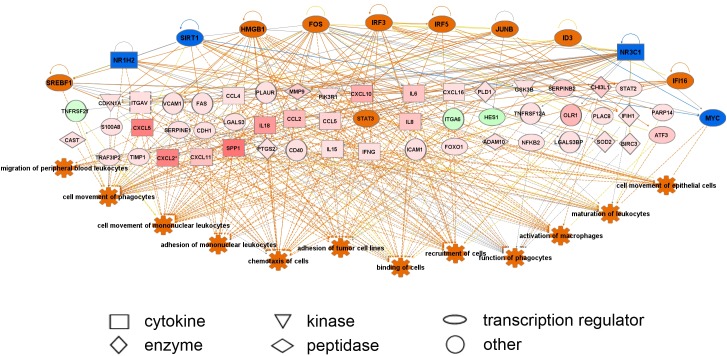
Regulator effect network analysis in IPA identifying altered regulators and networks in granulosa cells cultured *in vitro*. Orange and blue are predicted up and down regulated regulators on top, and the pink and green represent up and down regulated genes from the array data with the intensity of colour reflecting the degree of change. The pathways altered are listed at the bottom and orange indicates they are up regulated *in vitro*.

A subset of differentially regulated genes (4-fold change, FDR *P* < 0.05) was uploaded to the Ingenuity database and GOEAST for network, pathway and function analyses (Fig 3). This subset (n = 604 genes) is listed in S1 Table. Ingenuity Pathway Analyses and GOEAST analyses identified that cell adhesion and extracellular matrix related pathways were up regulated *in vitro*. Regulator effect network analysis (Fig 4) identified many effects on cell adhesion, movement, binding, migration and chemo-attraction and macrophage functions including phagocytosis. The upstream regulators that were predicted to be switched on included *SREB1*, *HMGB1*, *FOS*, *IRF2*, *IRF5*, *JUNB*, *ID3* and *IFI16* those predicted to be switched off included *NR1H2*, *SIRT1*, *NR3C1* and *MYC*.

To compare transcriptional processes of granulosa cells *in vitro* under specific conditions with those known to occur naturally *in vivo* such as atresia or maturation prior to ovulation, we decided to compare the differentially regulated dataset between *in vitro* and *in vivo* granulosa cells with the small atretic versus healthy follicle granulosa gene set [11], and with the small healthy follicle versus large healthy follicle granulosa gene set [15], from previous studies. S2A Fig shows that for granulosa cells 39.8% and 13.9% of genes differentially expressed (4-fold different between groups with FDR *P* <0.05) between *in vivo* and *in vitro* conditions were also differentially expressed in atresia and upon growth to large follicle sizes, respectively, indicating the majority of changes upon culture were not associated changes that would be observed with growth or atresia of follicles. We additionally checked to see if there was any potential thecal contamination that could have artefactually contributed to these results (S2B Fig). As only 17.5% of differentially expressed genes between the *in vivo* and *in vitro* (4-fold different between groups with FDR *P* < 0.05) were higher in the theca versus granulosa cells in the *in vivo* arrays and the thecal-specific markers *CYP17* and *INSL3* were not present in this group, any potential thecal contamination of granulosa cells is unlikely to have contributed to the results.

### Comparisons of in vivo and in vitro theca cell arrays

The expression of the housekeeping genes was similar between the *in vivo* and *in vitro* situations for thecal cells with the mean probe intensity of the housekeeping genes GAPDH, ACTB, PPIA and MRPL32 on the *in vitro* chips being 1.254, 0.997, 0.714 and 0.881-fold of that of the *in vivo* chips, respectively.

PCA (Fig 2B) and hierarchical clustering (S3 Fig) were applied to the *in vitro* and *in vivo* thecal cell array data. These two methods separated the two groups from each other with a far greater spread amongst the *in vivo* group indicating greater heterogeneity in gene expression amongst the *in vivo* group. The differentially expressed genes are listed in Tables 3 and 4. There was a high proportion of genes related to immune function in the group up regulated *in vitro* including; those which encode the major histocompatibility complex (classes I, *BOLA-N JSP*.*1*, *BOLA-A*; class II; *BOLA-DQA2*, *BOLA-DQA5*, *CD74*); interleukins *IL18*, *IL6* and *IL8* and the inflammatory chemokines *CXCL6*, *CXCL2*, *CCL5* and *CCL3*. Many other genes associated with immune responses were also more abundant, such as interferon gamma (S2 Table). Those genes which constitute the less abundant group *in vitro* were heterogeneous in function ranging from developmental differentiation for example *SHISA2*, *ALDH1A2* and *FRZB* to extracellular matrix such as *ASPN*, *COL16A1* and *COL13A1*.

**Table 3:** Top 50 genes that were most differentially up regulated *in vitro* in thecal cells.

Gene Symbol	Fold-Change	Gene Symbol	Fold-Change
*BOLA /// BOLA*	213.7	*IL6*	23.6
*CFB*	123.4	*IL8*	23.1
*NT5E*	59.4	*BoLA /// BOLA /// BOLA-A*	23.1
*IDO1*	58.3	*TNC*	22.8
*SPP1*	55.2	*TNFAIP6*	21.6
*CXCL6*	50.8	*ATF3*	21.5
*CXCL2*	49.9	*CHI3L1 /// LOC788983*	21.4
*GBP5*	45.3	*IRF1*	21.0
*ANKRD1*	43.2	*SERPINB2*	20.3
*BoLA /// BOLA-A*	43.0	*MMP9*	20.2
*IL18*	40.8	*GRO1*	19.5
*NUPR1*	40.8	*C1R*	19.4
*C1S*	36.0	*CLDN1*	18.6
*SAA3*	34.6	*SRRM2*	18.1
*CXCL10*	32.6	*CD74*	17.5
*GPNMB*	30.1	*CCL5*	17.4
*OLR1*	29.2	*POSTN*	17.1
*CCL2*	27.9	*ARHGAP29*	16.8
*BOLA-N /// JSP*.*1*	27.8	*EFEMP1*	16.8
*GBP4 /// LOC507055*	27.2	*CCL3*	16.2
*ERAP2*	27.2	*ISG20*	15.6
*MX2*	27.1	*BOLA-DQA5*	15.5
*CXCL11*	25.1	*AP2B1*	15.2
*WARS*	24.3	*BOLA-DQA2*	14.7
*RASSF4*	24.1	*EFR3A*	14.5

**Table 4:** Top 50 genes that were most differentially down regulated *in vitro* in thecal cells.

Gene Symbol	Fold-Change	Gene Symbol	Fold-Change
*CXCL14*	76.5	*APLNR*	11.0
*SHISA2*	57.2	*LMCD1*	10.7
*DUSP12*	26.0	*PRMT1*	10.5
*COLEC11*	20.3	*PTH1R*	10.4
*AQP1*	17.9	*CCL14*	10.1
*HMGB3*	17.7	*PABPN1*	10.0
*DHRS3*	17.7	*C1QA*	9.9
*BRB*	17.4	*NALCN*	9.9
*GPC3*	16.6	*CD34*	9.8
*HPGD*	16.0	*TCF21*	9.8
*HSD17B1*	15.5	*COL13A1*	9.6
*SDPR*	13.7	*HEYL*	9.1
*ASPN*	13.6	*AKAP8L*	8.6
*ALDH1A2*	13.2	*ISLR*	8.6
*PLIN5*	12.5	*AFAP1L1*	8.5
*CLEC14A*	12.4	*GANAB*	8.4
*TMEM88*	12.3	*MEOX2*	8.4
*RGS5*	12.2	*TM4SF18*	8.4
*LRRC70*	12.0	*PLXND1*	8.2
*LAMA2 /// LOC100848461*	11.9	*FAM64A*	8.2
*FRZB*	11.8	*HFM1*	8.0
*COL16A1 /// LOC100849968*	11.8	*RBM3*	7.9
*AMH*	11.5	*CDKN1C*	7.6
*RNASE6*	11.4	*TFF2*	7.5
*EGFLAM /// LOC100847583*	11.4	*C1QC*	7.5

**Fig 5:**
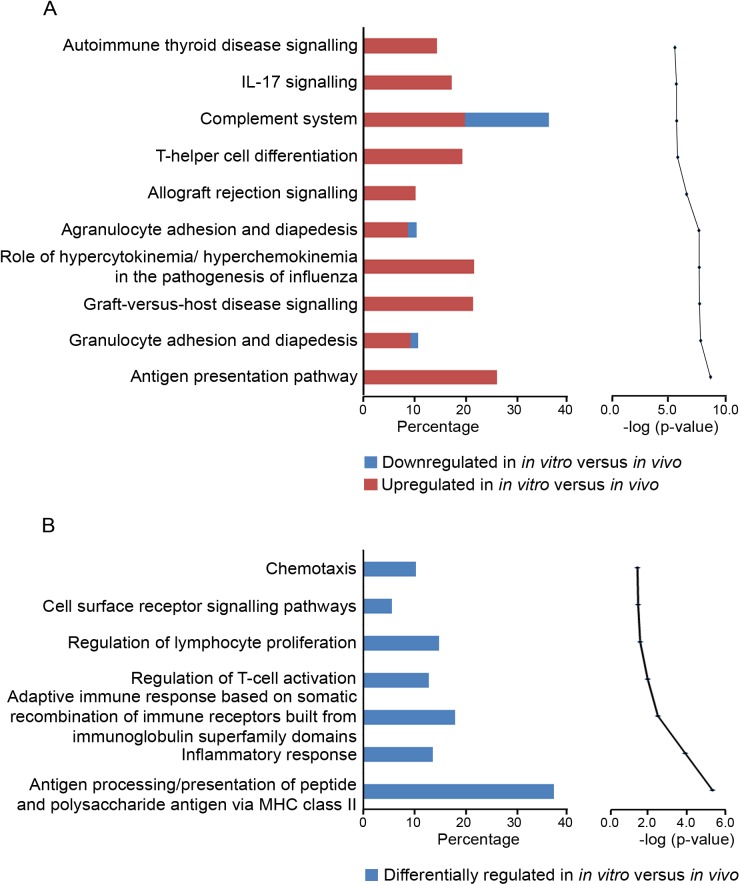
**Top canonical pathways in thecal cells mapped in IPA (A) and GO terms (B) classified under biological process**. In (A) on the left, the percentage of genes from the data set that map to each canonical pathway which are up regulated (in red) and down regulated (in blue) *in vitro* with respect to *in vivo* are shown. On the right, these pathways are then ranked from the highest to lowest degree of association based on multiple correction testing for the Benjamini-Hochberg False Discovery Rate (A). In (B) on the left, the percentage of genes from the data set that map to each GO term which are differentially regulated (in blue) *in vitro* with respect to *in vivo* are shown. These pathways are ranked from the highest to lowest degree of association using the Benjamini-Yuketeli test for multiple corrections (bottom to top in graphs on right).

**Fig 6:**
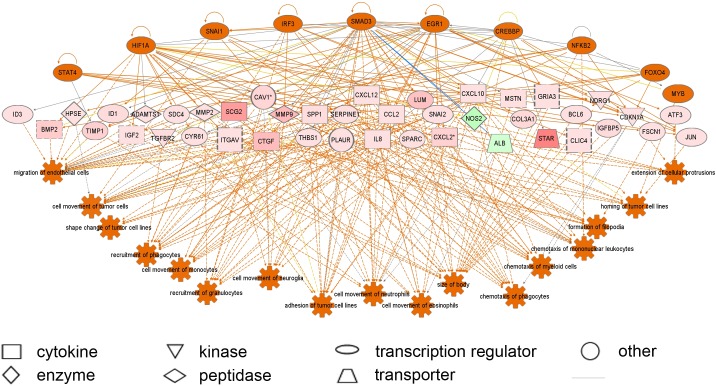
Regulator effect network analysis in IPA identifying altered regulators and networks in thecal cells cultured *in vitro*. Orange and blue are predicted up and down regulated regulators on top, and the pink and green represent up and down regulated genes from the array data with the intensity of colour reflecting the degree of change. The pathways altered are listed at the bottom and orange indicates they are up regulated *in vitro*.

A subset of differentially regulated genes (> 4-fold change, FDR *P* < 0.05) was uploaded to the Ingenuity database and GOEAST for network, pathway and function analyses (Fig 5). This subset (n = 610 genes) is listed in S2 Table. IPA and GOEAST analyses identified pathways related to immune response to be up regulated *in vitro*. Regulator effect network analysis (Fig 6) identified many effects on cell movement, adhesion, migration, homing, filopodia formation, extension of cell processes and chemo-attraction. The upstream regulators that were predicted to be switched on included *STAT4*, *HIF1A*, *SNAI1*, *IRF3*, *SMAD3*, *EGR1*, *CREBP*, *NFKB2*, *FOXO4* and *MYB*.

Similar to the comparison described for granulosa cells above, thecal transcriptional profiles associated with cultured cells and atretic follicles revealed that there was only 3.6% overlap in the genes differentially expressed between these conditions and healthy follicles *in vivo*. Furthermore, only one gene showed altered expression during antral follicle development, out of the total number of 604 due to the effect of *in vitro* culture.

## Discussion

*In vitro* culture models utilising isolated granulosa cells and cells from the thecal layers of ovarian follicles have been used extensively to produce data to create and test hypotheses about ovarian follicular development [21–25]. However, it is generally well recognised that *in vitro* conditions will likely alter the behaviour of the cells once removed from their normal follicular environment where they are exposed to a myriad of factors derived from neighbouring somatic cells, the oocyte and from the circulation. To identify which behaviours and genes are altered upon culture we therefore conducted analyses of microarrays of RNA expression profiles of both bovine granulosa cells and cells from theca interna layer to compare the transcriptomes of cultured cells with those of their *in vivo* counterparts.

Both granulosa cell and cells from the thecal layers behaved similarly in some respects. Both hierarchical clustering and PCA indicated that the *in vitro* transcriptomes were different to the *in vivo* transcriptomes, and that culture produced a more uniform expression pattern across the whole mRNA pool in both cell types. This might have been expected as the cultures were conducted for a 6-day period and the composition of the media with respect to added hormones and factors were constant. However, it could reflect that the *in vivo* data were derived from individual follicles whereas the *in vitro* data were from pools of approximately 50 follicles.

Ingenuity Pathway Analyses and GOEAST analyses of granulosa cells identified that cell adhesion and extracellular matrix related pathways were up regulated *in vitro*. Regulator effect network analysis identified many effects on cell adhesion, movement, binding, migration and chemo-attraction. Clearly the culture conditions in two dimensions on plastic, activated granulosa cell movement and cell matrix interactions. Indeed this would be consistent with the observation that the mono-dispersed cells granulosa cells plated out in the culture wells re-aggregate into 3-dimensional clumps over the course of the culture period [5,10]. Regulator effect network analysis of thecal cell cultures also identified many effects on cell movement, adhesion, migration, homing, filopodia, extension of cell processes and chemo-attraction, probably indicating interactions associated with attachment and interaction with the culture dish and cellular clumping [7,9]. The upstream regulators in granulosa cells that were predicted to be switched on included *SREB1*, *HMGB1*, *FOS*, *IRF3*, *IRF5*, *JUNB*, *ID3* and *IFI16* and those predicted to be switched off included *NR1H2*, *SIRT1*, *NR3C1* and *MYC*. Notably, only one of these, *IRF3*, is specifically common with the upstream regulators in thecal cell cultures that were predicted to be switched on and that also included *STAT4*, *HIF1A*, *SNAI1*, *IRF3*, *SMAD3*, *EGR1*, *CREBP*, *NFKB2*, *FOXO4* and *MYB*. Members of the interferon regulatory family (*IRF*) that regulate transcription of interferon genes were identified in the upstream regulator pathways (*IRF3* and *IRF5*) and also identified in the list of differentially up regulated genes in both cell types (*IRF1*).

This type of effect has been reported for other cell types where adherence to plastic initiates cytoskeletal changes in epithelial cells and immune signalling in macrophages [26–28]. Granulosa cells, like macrophages, express Toll-Like Receptors (TLRs) and are capable of responding to bacterial endotoxins and inflammatory cytokines [29]. It is possible that the culture system used in our experiments produced these kinds of phenotypic changes in the granulosa cells. This has possible implications for the design of experiments which investigate granulosa cell behaviour, whereby the surface substrate composition and rigidity become important due to the effect on specific signalling pathways as shown here.

Our transcriptome analysis also indicated that phagocytosis was activated in cultures of granulosa cells. Macrophages can undertake phagocytosis for immunity (M1) or tissue repair (M2). However, the bovine membrana granulosa is free of immune cells until follicle atresia or ovulation commences [17,30] and granulosa cells have the ability to carry out phagocytosis [31], as can other epithelial cells [32]. Additionally there did not appear to be an activation of an innate immune response in the cultured granulosa cells despite these cells having this capability [29,33] as there was no increase in abundance of IL-1β or IL-6. Hence it is likely that phagocytosis is carried out by the granulosa cells *in vitro* as they can do *in vivo* to remove dead and dying neighbouring granulosa cells [31].

Ingenuity Pathway Analyses and GOEAST analyses of thecal cells identified substantial up regulation of immune responses and the major histocompatibility complex molecules (MHC Class 1: *BOLA-N JSP*.*1*, *BOLA-A*; MHC class 2: *BOLA-DQA2*, *BOLA-DQA5*, *CD74*); interleukins IL18, *IL6* and *IL8*. The inflammatory chemokines *CXCL6*, *CXCL2*, *CCL5*, *CCL3* and interferon gamma were also up regulated upon culture. These responses appear to be driving an innate immune response but possibly also initiating an adaptive immune response. MHC class II antigens are found in antigen presenting cells such as dendritic cells and macrophages. The vast majority of the immune cells in the theca interna layer are macrophages and neutrophils with relatively few T lymphocytes present [34–36] and these would have undoubtedly been present in the thecal interna cell cultures. The question arises: why would an immune response be triggered at all? The culture medium was serum free, but with additives of insulin or serum albumin which were bovine in origin and highly unlikely to induce expression of these genes involved in an immune response. The media also contained penicillin, streptomycin and amphotericin B but these agents would not be predicted to initiate an immune response. Another possibility is that microbial contamination during the culture period up regulated immune response genes but no overt signs of microbial contamination were ever observed. There is the possibility that the response is to dead cells or to non-self antigens, as the theca preparations were from pools of follicles from different animals. Thus, whilst we do not know the type of immune response or its cause it is clear that, *in vitro*, increased expression of inflammatory cytokines occurs and this could possibly affect the outcome and/or interpretation of any *in vitro* experiment using this culture system.

In conclusion, *in vitro* culture of both granulosa cells and cells from the theca interna layer alters the behaviour and transcriptomes of these cells, making the transcriptomes more uniform but with up regulation of cell behaviour associated with cell matrix interactions and with adhesion. Additionally, with cultured thecal interna cells there was clear evidence of an immune response likely mediated by leukocytes present within those cultures. These changes are important to be aware of when interpreting data derived from such cell culture systems. The approach we have taken could also be applied more widely to design and validate cell culture systems that more faithfully mimic *in vivo* situations.

## Supporting information

S1 Fig Unsupervised hierarchical clustering for granulosa cells. The unsupervised hierarchical clustering across all probe sets (n = 24,182) for 17 arrays from granulosa cells was performed using the Euclidian dissimilarity algorithm with the average linkage method in Partek Genomics Suite. The heatmap represents the distribution of normalised signal intensity, grouping by pattern similarity for both probe set and array. Abbreviations are as for Fig 2A.
(JPG)

S2 Fig Venn diagrams of genes which are differentially expressed in granulosa cells from small antral follicles. All statistical cut-offs for differential expression were > 4-fold change with FDR *P* < 0.05. In **A,** the upper diagram shows numbers of genes with altered expression due to culture or atresia, the lower diagram shows numbers of genes with differences in expression due to culture or maturation. Intersected regions indicate those genes which are shared between different conditions and the proportion of the total numbers with changed expression resulting from culture alone. **B** shows those genes which are differentially regulated during atresia and culture and also those genes up regulated in the theca interna *in vivo*, indicating the proportion of genes with altered expression common to the different cell types of the follicle.
(JPG)

S3 Fig Unsupervised hierarchical clustering for thecal cells. The unsupervised hierarchical clustering occurred across all probe sets (n = 24,182) for 18 arrays from thecal cells was performed using the Euclidian dissimilarity algorithm with the average linkage method in Partek Genomics Suite. The heatmap represents the distribution of normalised signal intensity, grouping by pattern similarity for both probe set and array. Abbreviations are as for Fig 2B.
(JPG)

S1 Table Genes which are differentially regulated *in vitro* compared with *in vivo* in granulosa cells. The complete gene list is showing full names of the genes, fold changes, signal intensities and FDR values (> 4-fold change, FDR *P* < 0.05).
(PDF)

S2 Table Genes which are differentially regulated *in vitro* compared with *in vivo* in cells from the theca layers. The complete gene list showing full names of the genes, fold changes, signal intensities and FDR values (> 4-fold change, FDR *P* < 0.05).
(PDF)
