# Supplementary figures and images for: Transcriptomal profiling of bovine ovarian granulosa and theca interna cells in primary culture in comparison with their in vivo counterparts
Source: PLoS One. 2017 Mar 10;12(3):e0173391. doi: 10.1371/journal.pone.0173391 (PMC5345798; doi:10.1371/journal.pone.0173391)

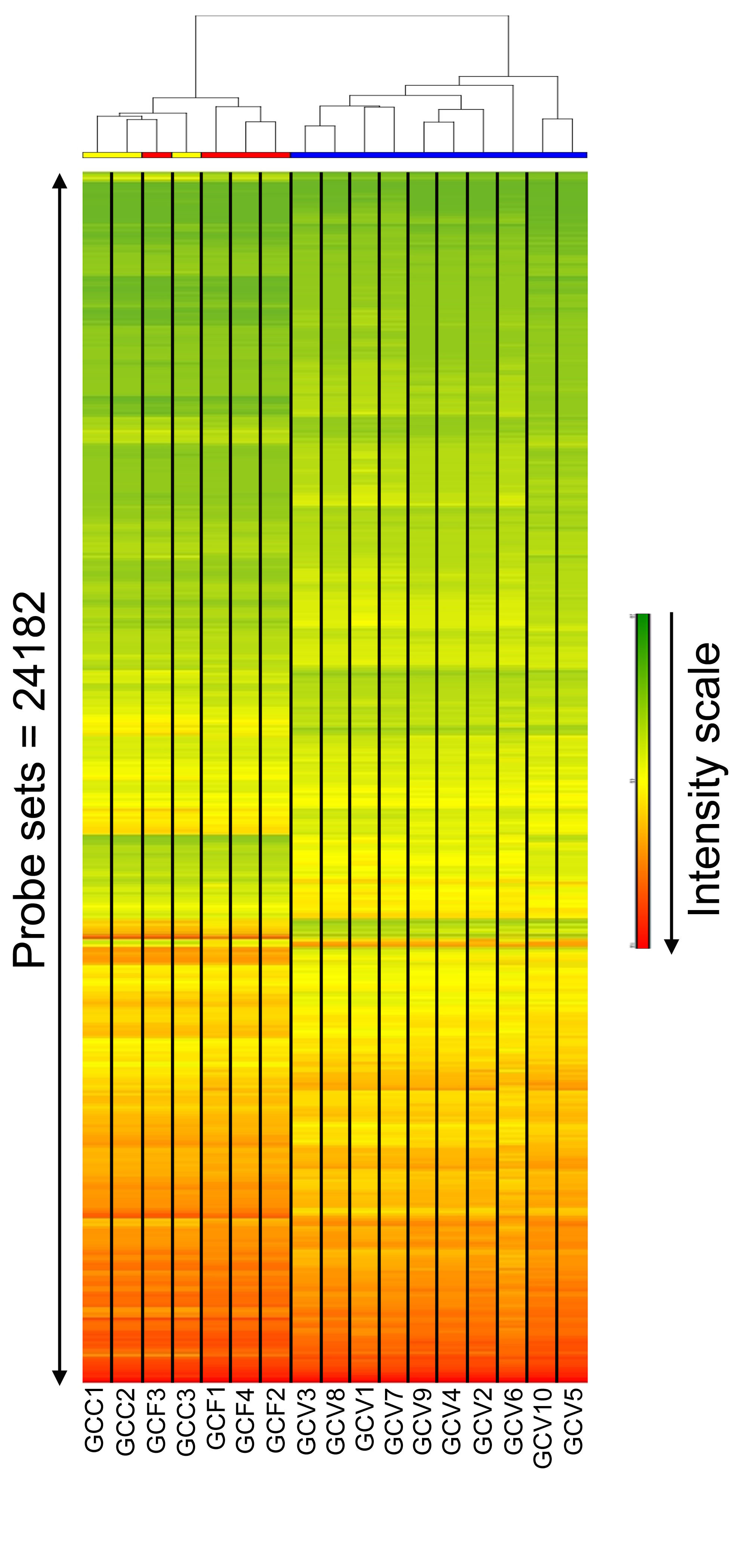

Supplement: S1 Fig — The unsupervised hierarchical clustering across all probe sets (n = 24,182) for 17 arrays from granulosa cells was performed using the Euclidian dissimilarity algorithm with the average linkage method in Partek Genomics Suite. The heatmap represents the distribution of normalised signal intensity, grouping by pattern similarity for both probe set and array. Abbreviations are as for Fig 2A. (JPG) [file pone.0173391.s001.jpg]

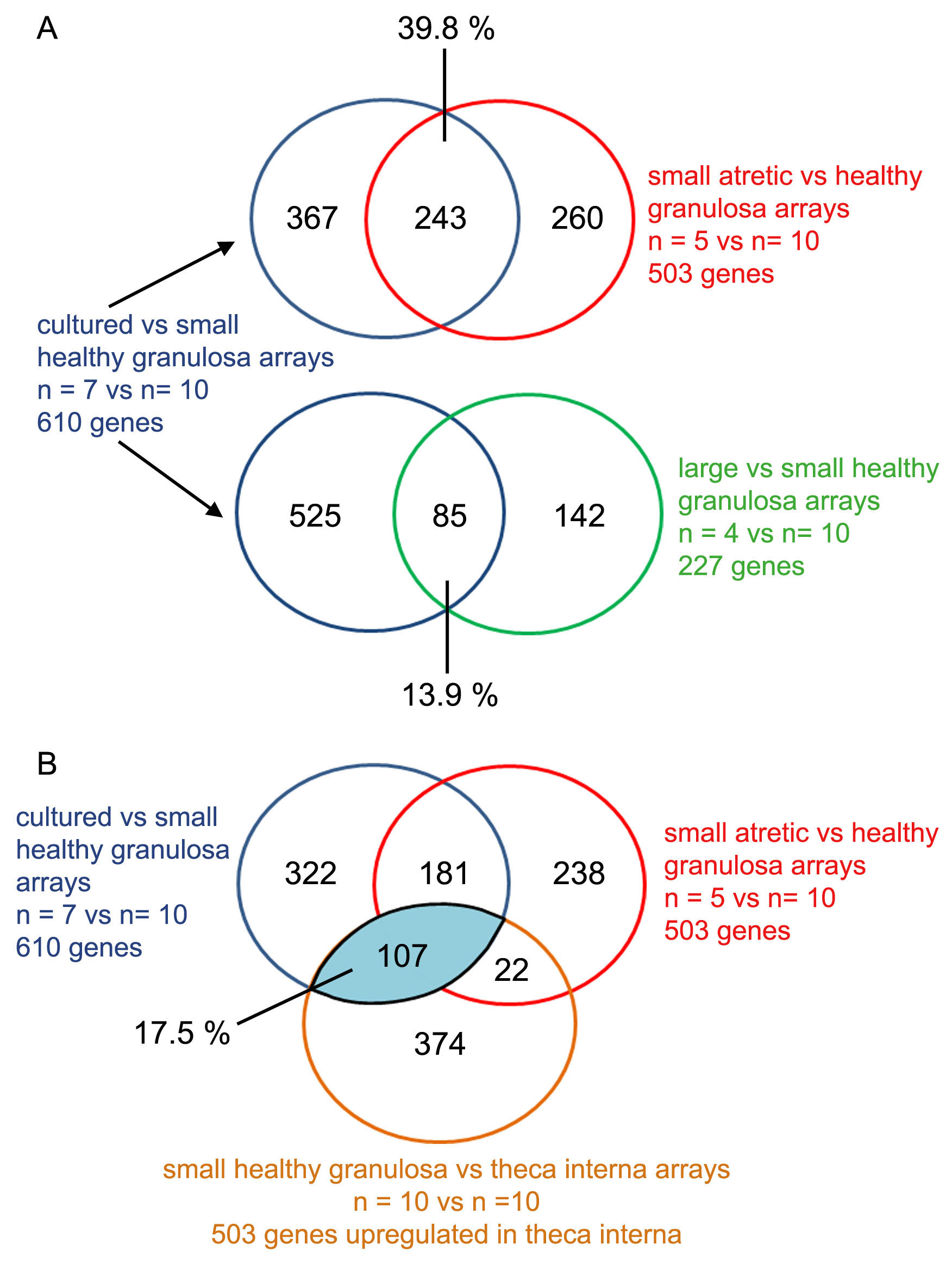

Supplement: S2 Fig — All statistical cut-offs for differential expression were > 4-fold change with FDR P < 0.05. In A, the upper diagram shows numbers of genes with altered expression due to culture or atresia, the lower diagram shows numbers of genes with differences in expression due to culture or maturation. Intersected regions indicate those genes which are shared between different conditions and the proportion of the total numbers with changed expression resulting from culture alone. B shows those genes which are differentially regulated during atresia and culture and also those genes up regulated in the theca interna in vivo, indicating the proportion of genes with altered expression common to the different cell types of the follicle. (JPG) [file pone.0173391.s002.jpg]

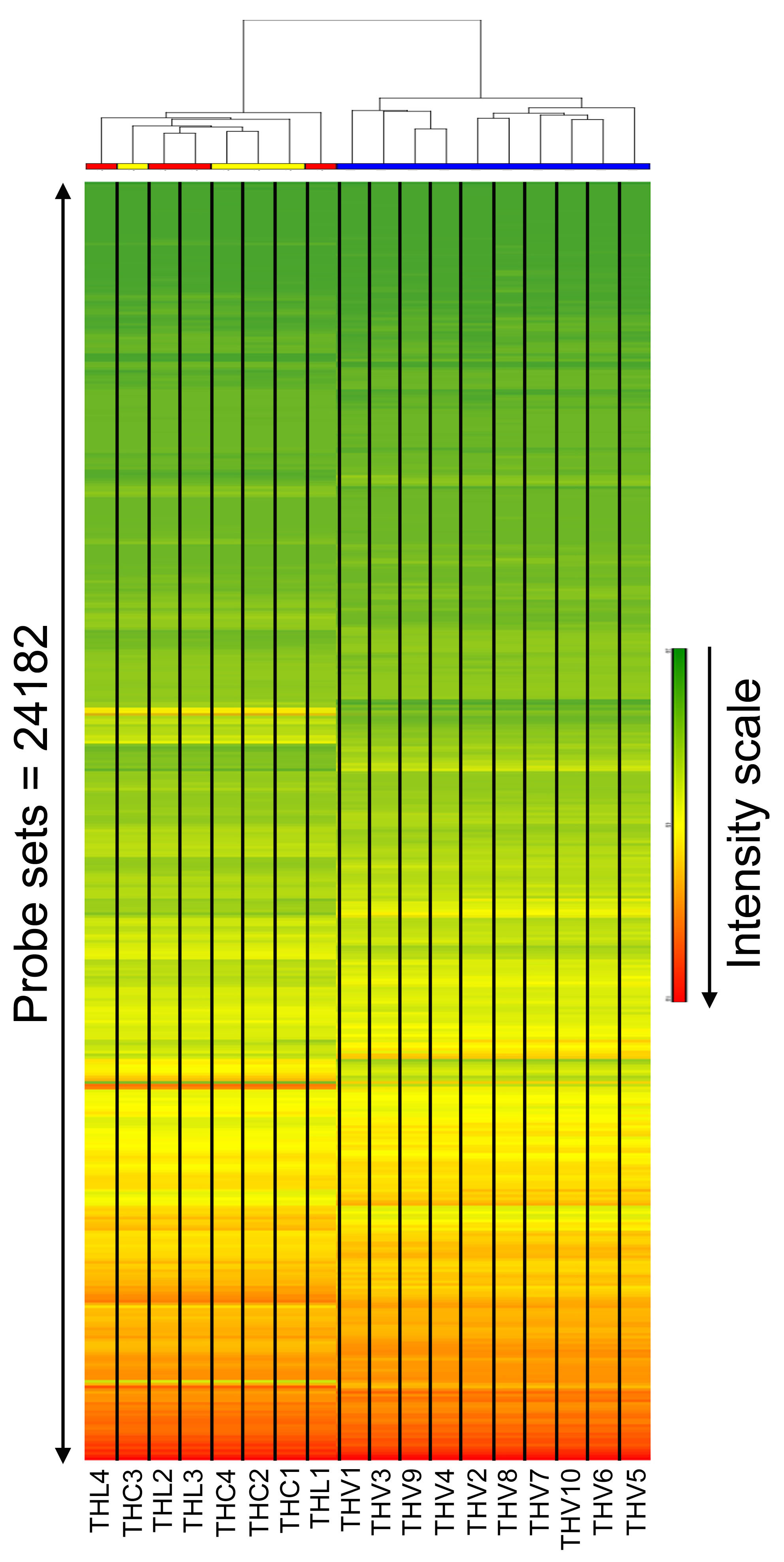

Supplement: S3 Fig — The unsupervised hierarchical clustering occurred across all probe sets (n = 24,182) for 18 arrays from thecal cells was performed using the Euclidian dissimilarity algorithm with the average linkage method in Partek Genomics Suite. The heatmap represents the distribution of normalised signal intensity, grouping by pattern similarity for both probe set and array. Abbreviations are as for Fig 2B. (JPG) [file pone.0173391.s003.jpg]
