# Supplementary material for: Transcriptomal profiling of bovine ovarian granulosa and theca interna cells in primary culture in comparison with their in vivo counterparts
Source: PLoS One. 2017 Mar 10;12(3):e0173391. doi: 10.1371/journal.pone.0173391 (PMC5345798; doi:10.1371/journal.pone.0173391)
Supplement: S1 Table — The complete gene list is showing full names of the genes, fold changes, signal intensities and FDR values (> 4-fold change, FDR P < 0.05). (PDF) [file pone.0173391.s004.pdf]

S1 Table. **Genes which are differentially regulated *in vitro* compared with *in vivo* in granulosa cells.**

**Gene Symbol**	**Gene Title**	**RefSeq Transcript ID**	**Fold-Change**	**Log_2 Mean Intensity**		**FDR *P*- value**
				In vitro	***In vivo***	
TNFAIP6	tumor necrosis factor, alpha-induced protein 6	NM_001007813	316.1	12.7	4.4	6.4E-15
XCL1	chemokine (C motif) ligand 1	NM_175716	145.2	12.3	5.1	7.3E-13
STC1	stanniocalcin 1	NM_176669	102.7	12.0	5.3	4.2E-14
ANKRD1	ankyrin repeat domain 1 (cardiac muscle)	NM_001034378	102.1	12.8	6.1	3.6E-12
SFRP2	secreted frizzled-related protein 2	NM_001034393	97.1	10.9	4.3	6.2E-11
PLAT	plasminogen activator, tissue	NM_174146	79.4	12.5	6.2	1.0E-10
CXCL6	chemokine (C-X-C motif) ligand 6 (granulocyte chemotactic protein 2)	NM_174300	67.1	10.5	4.4	1.6E-12
DCN	decorin	NM_173906	66.7	12.2	6.1	5.7E-12
STAR	steroidogenic acute regulatory protein	NM_174189	62.8	11.1	5.1	7.9E-14
PDGFRA	platelet-derived growth factor receptor, alpha polypeptide	NM_001192345	57.8	10.6	4.8	1.4E-11
PTX3	pentraxin 3, long	NM_001076259	55.1	10.0	4.2	8.8E-13
IL18	interleukin 18 (interferon-gamma-inducing factor)	NM_174091	52.6	11.0	5.2	5.0E-11
DKK3	dickkopf homolog 3 (Xenopus laevis)	NM_001100306	50.1	10.5	4.8	5.1E-10
SCG2	secretogranin II	NM_174176	49.0	10.4	4.8	1.0E-11
NID2	nidogen 2 (osteonidogen)	NM_001102065	49.0	10.8	5.2	5.4E-11
CLDN11	claudin 11	NM_001035055	43.4	11.1	5.7	2.9E-10
C27H8orf4	chromosome 27 open reading frame, human C8orf4	NM_001035490	41.6	9.8	4.4	3.6E-12
RND3	Rho family GTPase 3	NM_001191158 /// XM_003585297	39.1	11.4	6.2	2.0E-09
THBS2	thrombospondin 2	NM_176872	38.6	11.6	6.3	1.1E-11
TNFRSF12A	tumor necrosis factor receptor superfamily, member 12A	NM_001206327 /// NM_001206328	38.2	11.1	5.8	5.0E-12
LGALS3	lectin, galactoside-binding, soluble, 3	NM_001102341	35.6	11.6	6.4	2.8E-09
MMP9	matrix metallopeptidase 9 (gelatinase B, 92kDa gelatinase, 92kDa type IV collagenase)	NM_174744	34.9	9.8	4.7	2.9E-12
CTGF	connective tissue growth factor	NM_174030	33.2	12.3	7.3	5.1E-08
COL1A2	collagen, type I, alpha 2	NM_174520	32.8	11.8	6.7	5.3E-10

PRKAG2	protein kinase, AMP-activated, gamma 2 non-catalytic subunit	XM_002686979 /// XM_002704191	31.0	10.9	6.0	6.5E-11
LUM	lumican	NM_173934	30.5	11.8	6.9	4.4E-10
S100A12	S100 calcium binding protein A12	NM_174651	29.8	9.5	4.6	2.5E-09
NUAK1	NUAK family, SNF1-like kinase, 1	NM_001205496	28.3	10.7	5.9	9.5E-11
COL5A2	collagen, type V, alpha 2	XM_003581798 /// XM_003585717	27.5	10.8	6.0	7.9E-14
LOC100336535	transcription factor SOX-9-like	XR_083993	27.3	10.3	5.5	1.8E-09
BMP2	bone morphogenetic protein 2	NM_001099141	26.2	10.0	5.2	2.0E-11
CLCA3	chloride channel regulator 3	NM_181018	26.1	9.2	4.5	1.3E-11
SGK1	serum/glucocorticoid regulated kinase 1	NM_001102033	24.9	9.7	5.1	6.9E-12
CTSH	cathepsin H	NM_001034385	24.7	9.8	5.2	1.4E-11
GFPT2	glutamine-fructose-6-phosphate transaminase 2	NM_001076883	24.7	10.7	6.1	1.9E-11
PLK2	polo-like kinase 2	NM_001192245	24.3	8.6	4.0	7.6E-13
TIMP1	TIMP metallopeptidase inhibitor 1	NM_174471	23.5	12.8	8.2	1.4E-10
CXCL2	chemokine (C-X-C motif) ligand 2	NM_174299	22.8	8.5	4.0	1.0E-09
NCAM1	neural cell adhesion molecule 1	NM_174399	22.5	9.7	5.2	5.5E-12
AXL	AXL receptor tyrosine kinase	XM_002695068 /// XM_594754	22.4	9.4	4.9	1.3E-09
GIMAP8 /// LOC100847526	GTPase, IMAP family member 8 /// GTPase IMAP family member 8-like	XM_002687147 /// XM_003582163 /// XM_003586027 /// XM_610014	21.0	9.5	5.1	2.6E-11
FN1	fibronectin 1	NM_001163778	20.9	10.0	5.6	8.5E-11
CAV1	caveolin 1, caveolae protein, 22kDa	NM_174004	20.8	9.3	4.9	2.1E-11
ANXA1	annexin A1	NM_175784	19.3	11.6	7.3	1.9E-10
OLR1	oxidized low density lipoprotein (lectin-like) receptor 1	NM_174132	19.3	9.1	4.8	7.6E-10
COL3A1	collagen, type III, alpha 1	NM_001076831	19.2	10.9	6.7	2.0E-09
LOC100138326 /// LOC100297468 /// LOC786683 /// MGC127538	CD24 antigen /// cD24 molecule-like /// CD24 antigen /// uncharacterized protein MGC127	XM_001254294 /// XM_001788106 /// XM_002684144 /// XM_002684248 ///	18.9	8.7	4.5	1.9E-09

		XM_002687558 /// XM				
TRIB1	tribbles homolog 1 (Drosophila)	NM_001101105	18.5	9.4	5.2	5.9E-09
IGFBP5	insulin-like growth factor binding protein 5	NM_001105327	18.5	10.3	6.1	4.1E-11
MAP1LC3C	microtubule-associated protein 1 light chain 3 gamma	NM_001101058	18.4	8.4	4.2	1.4E-11
NDP	Norrie disease (pseudoglioma)	NM_001046090	17.8	10.1	5.9	3.7E-07
DCLK1	doublecortin-like kinase 1	NM_001109962	17.6	9.3	5.2	8.3E-11
POSTN	periostin, osteoblast specific factor	NM_001040479	17.4	9.7	5.6	1.2E-08
ID1	inhibitor of DNA binding 1, dominant negative helix-loop-helix protein	NM_001097568	17.3	9.1	5.0	4.4E-11
CAPG	capping protein (actin filament), gelsolin-like	NM_178574	17.1	9.4	5.3	5.3E-10
TGFBR2	transforming growth factor, beta receptor II (70/80kDa)	NM_001159566	17.0	9.1	5.0	1.4E-11
SPP1	secreted phosphoprotein 1	NM_174187	16.7	9.4	5.3	5.4E-05
OXT	oxytocin, prepropeptide	NM_176855	16.7	11.2	7.2	1.3E-08
LOC100848920 /// RGS2	regulator of G-protein signaling 2-like /// regulator of G-protein signaling 2, 24kDa	NM_001075596 /// XM_003583217 /// XM_003587052	16.6	10.8	6.7	4.3E-06
PVR	poliovirus receptor	XM_002695088 /// XM_605244	16.6	9.8	5.8	8.9E-11
SLC17A5	solute carrier family 17 (anion/sugar transporter), member 5	NM_001205974	16.3	12.2	8.2	5.4E-12
SERPINE1	serpin peptidase inhibitor, clade E (nexin, plasminogen activator inhibitor type 1), me	NM_174137	16.2	9.9	5.9	8.5E-10
SLC39A8	solute carrier family 39 (zinc transporter), member 8	NM_001205630	15.9	11.0	7.0	1.0E-09
GRO1	chemokine (C-X-C motif) ligand 1 (melanoma growth stimulating activity, alpha)	NM_175700	15.3	7.7	3.8	2.2E-09
CDKN1A	cyclin-dependent kinase inhibitor 1A (p21, Cip1)	NM_001098958	15.2	11.5	7.6	2.2E-12
LOC787239	DKFZP459P193 protein-like	XM_001254697	14.8	9.5	5.7	6.1E-09
PRSS23	protease, serine, 23	NM_001080306	14.3	10.6	6.8	6.5E-09
CA2	carbonic anhydrase II	NM_178572	14.2	8.1	4.3	7.3E-12
LOC100850907 /// MAPK6	mitogen-activated protein kinase 6-like /// mitogen-activated protein kinase 6	XM_002690965 /// XM_002706554 /// XM_003585063	14.1	8.5	4.7	1.8E-09
EFNA5	ephrin-A5	NM_001076432	14.1	10.4	6.5	8.7E-08

STEAP1	six transmembrane epithelial antigen of the prostate 1	NM_001205806	13.9	9.3	5.6	2.9E-08
BOLA /// BOLA	MHC class I antigen clone 2 /// MHC class I heavy chain	NM_001038518 /// NM_001040532 /// XM_002697336	13.7	8.0	4.2	7.7E-07
HSP90AA1	heat shock protein 90kDa alpha (cytosolic), class A member 1	NM_001012670	13.5	12.0	8.2	8.8E-06
SDC2	syndecan 2	NM_001034788	13.3	9.0	5.2	2.1E-10
AP2B1	adaptor-related protein complex 2, beta 1 subunit	NM_001075125 /// XM_003587384	13.0	8.9	5.2	5.2E-10
CLCA3 /// LOC784768	chloride channel regulator 3 /// calcium-activated chloride channel	NM_001242583 /// NM_181018	13.0	8.1	4.4	1.0E-09
NTS	neurotensin	NM_173945	12.5	9.3	5.7	7.3E-08
IER3	immediate early response 3	NM_001075202	12.5	10.3	6.6	1.4E-11
GNG2	guanine nucleotide binding protein (G protein), gamma 2	NM_174072	12.4	10.0	6.4	5.7E-09
PDPK1	3-phosphoinositide dependent protein kinase-1	XM_001788663 /// XM_002697862	12.2	9.2	5.6	1.2E-09
LOC512486	interferon-induced guanylate-binding protein 1	NM_001244229	12.2	8.7	5.1	5.3E-09
CLDN1	claudin 1	NM_001001854	12.2	10.3	6.7	5.9E-08
CD99	CD99 molecule	NM_001244214	12.0	9.3	5.7	4.8E-07
MMP2	matrix metallopeptidase 2 (gelatinase A, 72kDa gelatinase, 72kDa type IV collagenase)	NM_174745	12.0	9.6	6.0	6.9E-09
LHFP	lipoma HMGIC fusion partner	NM_001077990	11.8	8.6	5.0	2.1E-10
MICAL1	microtubule associated monoxygenase, calponin and LIM domain containing 1	NM_001081582	11.6	8.7	5.2	3.8E-10
CTSK	cathepsin K	NM_001034435	11.5	10.4	6.9	3.5E-09
BCL6	B-cell CLL/lymphoma 6	NM_001206450	11.5	11.0	7.5	3.9E-09
FIBIN	fin bud initiation factor homolog (zebrafish)	NM_001015541	11.4	10.0	6.5	5.7E-12
ATF3	activating transcription factor 3	NM_001046193	11.4	9.3	5.8	2.1E-08
EFEMP1	EGF containing fibulin-like extracellular matrix protein 1	NM_001081717	11.2	8.2	4.7	8.2E-08
AMACR /// LOC787920	alpha-methylacyl-CoA racemase /// alpha-methylacyl-CoA racemase-like	XM_001255135 /// XM_002696385 /// XM_593629	11.0	10.6	7.1	2.1E-08
CTSS	cathepsin S	NM_001033615	11.0	8.9	5.5	2.8E-09

PLXDC2	plexin domain containing 2	NM_001077928	10.9	9.1	5.7	1.1E-09
CRABP2	cellular retinoic acid binding protein 2	NM_001008670	10.8	11.5	8.0	1.8E-07
KIAA0408	KIAA0408 ortholog	XM_001789720 /// XM_002690052	10.7	8.4	5.0	4.1E-09
LOC100851547 /// NMI	N-myc-interactor-like /// N-myc (and STAT) interactor	NM_001035098 /// XM_003581830	10.6	9.9	6.5	1.9E-11
ANXA2	annexin A2	NM_174716	10.3	12.4	9.0	1.8E-10
LOC100850955 /// MYLK	myosin light chain kinase, smooth muscle-like /// myosin light chain kinase	NM_176636 /// XM_003581710	10.2	9.1	5.8	1.2E-09
PKIB	protein kinase (cAMP-dependent, catalytic) inhibitor beta	NM_001114518	10.0	8.1	4.8	3.9E-10
LOC100848765	uncharacterized LOC100848765	XR_139527	10.0	10.4	7.1	1.4E-11
LOC100847211	uncharacterized LOC100847211	XM_003584343 /// XR_139747	10.0	10.8	7.5	2.0E-07
KCNE4	potassium voltage-gated channel, Isk-related family, member 4	NM_001081543	10.0	9.8	6.5	1.6E-07
NDRG1	N-myc downstream regulated 1	NM_001035009	9.9	9.4	6.1	1.1E-09
SERPINB2	serpin peptidase inhibitor, clade B (ovalbumin), member 2	NM_001192079	9.9	8.9	5.6	1.1E-07
PLIN2	perilipin 2	NM_173980	9.8	12.5	9.2	1.5E-08
GEM	GTP binding protein overexpressed in skeletal muscle	NM_001083732 /// NM_001276305	9.8	11.5	8.2	1.3E-06
PLXNB2	plexin B2	NM_001205629	9.7	8.9	5.6	1.9E-07
LOC100851475 /// LOC100852174 /// SRPX2	sushi repeat-containing protein SRPX2-like /// sushi repeat-containing protein SRPX2-li	NM_001014926 /// XM_003584298 /// XM_003585524	9.6	8.0	4.7	1.8E-12
RASAL2	RAS protein activator like 2	XM_002694157 /// XM_003587091 /// XM_612667	9.4	9.0	5.7	3.9E-09
ZNF385B	zinc finger protein 385B	NM_001083502	9.4	10.3	7.1	7.6E-09
IL8	interleukin 8	NM_173925	9.4	8.3	5.1	9.1E-08
ADAMTS1	ADAM metallopeptidase with thrombospondin type 1 motif, 1	NM_001101080	9.4	9.7	6.5	5.0E-08
BASP1	brain abundant, membrane attached signal protein 1	NM_174780	9.3	9.6	6.4	1.7E-09

ABCB1 /// ABCB4 /// LOC100296627 /// LOC785554	ATP-binding cassette, sub-family B (MDR/TAP), member 1 /// ATP-binding cassette, sub-fa	XM_002686717 /// XM_002686730 /// XM_002686731 /// XM_003582070 /// XM_003584756 /// XM	9.3	9.2	5.9	1.5E-11
MTPN	myotrophin	NM_203362	9.3	9.1	5.9	4.3E-07
DNER	delta/notch-like EGF repeat containing	XM_003585079 /// XM_003585774 /// XM_594984	9.2	7.6	4.4	8.6E-10
PSPH	phosphoserine phosphatase	NM_001046355	9.1	9.4	6.2	2.0E-11
SNAI2	snail homolog 2 (Drosophila)	NM_001034538	9.0	8.0	4.9	1.5E-11
MAR3	membrane-associated ring finger (C3HC4) 3	NM_001077941	9.0	8.0	4.8	1.3E-07
LOC100847619	uncharacterized LOC100847619	XR_138993 /// XR_139622	8.9	7.4	4.2	6.8E-09
DYNC1LI2	dynein, cytoplasmic 1, light intermediate chain 2	NM_001206152	8.9	11.4	8.3	1.1E-09
CAST	calpastatin	NM_001030318 /// NM_001030319 /// NM_001030320 /// NM_174003	8.8	10.5	7.4	2.1E-10
TSPO	translocator protein (18kDa)	NM_175776	8.8	10.5	7.4	9.5E-11
JUN	jun proto-oncogene	NM_001077827	8.8	11.2	8.1	2.7E-07
CRYAB	crystallin, alpha B	NM_174290	8.8	8.3	5.2	3.4E-07
CXCL16	chemokine (C-X-C motif) ligand 16	NM_001046095	8.7	8.8	5.7	2.5E-09
CPEB2	cytoplasmic polyadenylation element binding protein 2	XM_002688462 /// XM_002704640	8.6	10.6	7.5	1.9E-10
MICAL2	microtubule associated monoxygenase, calponin and LIM domain containing 2	XM_002693051 /// XM_613663	8.6	9.0	5.9	4.4E-08
SYNCRIP	synaptotagmin binding, cytoplasmic RNA interacting protein	XM_002690201 /// XM_002690202 /// XM_002705186 /// XM_003582613 /// XM_003586473 /// XM	8.5	9.3	6.2	5.1E-07

LOC100848375	uncharacterized LOC100848375	XR_138793 /// XR_139401	8.5	8.6	5.5	1.2E-11
CTR9	Ctr9, Paf1/RNA polymerase II complex component, homolog (S. cerevisiae)	NM_001205397	8.4	8.2	5.1	2.1E-10
MIR21	microRNA mir-21	NR_030880	8.3	10.5	7.4	4.8E-06
KLF6	Kruppel-like factor 6	NM_001035271	8.3	8.4	5.3	1.9E-08
ARHGEF3	Rho guanine nucleotide exchange factor (GEF) 3	XM_001250879 /// XM_002697002 /// XM_003583806 /// XM_003583807 /// XM_003587632 /// XM	8.3	7.6	4.6	4.7E-09
EPAS1	endothelial PAS domain protein 1	NM_174725	8.3	10.2	7.1	2.0E-07
EXT1	exostosin 1	NM_001098095	8.2	8.9	5.9	1.2E-06
AIF1L	allograft inflammatory factor 1-like	NM_001078079	8.1	10.2	7.2	7.3E-09
SPATS2L	spermatogenesis associated, serine-rich 2-like	NM_001192987	8.0	9.7	6.7	2.5E-09
KIAA1598 /// LOC100848050	KIAA1598 ortholog /// uncharacterized LOC100848050	NM_001110080 /// XM_003584108 /// XM_003584109 /// XM_003587898 /// XM_003587899 /// XR	7.8	9.9	6.9	8.6E-11
IFI30	interferon, gamma-inducible protein 30	NM_001101251	7.8	8.4	5.4	2.8E-08
PTPN5	protein tyrosine phosphatase, non-receptor type 5 (striatum-enriched)	NM_001102293	7.7	7.5	4.6	1.9E-11
TPR	translocated promoter region (to activated MET oncogene)	NM_001205623	7.7	7.1	4.1	9.2E-10
TAGLN	transgelin	NM_001046149	7.7	9.3	6.3	5.5E-07
CCL2	chemokine (C-C motif) ligand 2	NM_174006	7.7	8.3	5.3	2.7E-05
ZNF317	zinc finger protein 317	XM_002688767 /// XM_583362	7.6	9.1	6.2	2.4E-09
FHL3	four and a half LIM domains 3	NM_001034223	7.6	8.6	5.7	1.1E-09
MDK /// MDK	midkine-like /// midkine (neurite growth-promoting factor 2)	NM_173935 /// XM_002694376 /// XM_870711	7.6	9.4	6.5	2.2E-07

SEP6	septin 6	NM_001035430	7.6	7.5	4.6	5.0E-10
LMNA	lamin A/C	NM_001034053	7.5	9.1	6.2	3.6E-08
LAMB1	laminin, beta 1	NM_001206519	7.5	9.1	6.2	5.8E-07
CD9	CD9 molecule	NM_173900	7.5	11.2	8.3	1.2E-08
USP7	ubiquitin specific peptidase 7 (herpes virus-associated)	XM_002697958 /// XM_002703063	7.5	7.6	4.7	4.4E-08
SYTL2	synaptotagmin-like 2	NM_001102278	7.5	7.0	4.1	8.3E-08
DLC1	deleted in liver cancer 1	NM_001102493	7.4	7.5	4.6	1.1E-06
CSNK2A1	casein kinase 2, alpha 1 polypeptide	NM_174635	7.4	10.1	7.2	1.9E-09
GPNMB	glycoprotein (transmembrane) nmb	NM_001038065	7.4	8.2	5.3	1.1E-07
EFNA5	ephrin-A5	NM_001076432	7.4	9.2	6.3	2.4E-06
EIF4G3	eukaryotic translation initiation factor 4 gamma, 3	NM_001078002 /// XM_003581895 /// XM_003581896 /// XM_003581897 /// XM_003581898 /// XM	7.4	7.3	4.5	1.9E-08
ZNF292	zinc finger protein 292	NM_001192850	7.3	8.5	5.7	2.7E-07
MXRA7	matrix-remodelling associated 7	XM_001253301 /// XM_002696202 /// XM_003583641 /// XM_003583642 /// XM_003587485 /// XM	7.3	12.2	9.4	6.5E-11
SLITRK2	SLIT and NTRK-like family, member 2	XM_002699621 /// XM_591589	7.2	9.7	6.9	4.5E-06
ELOVL4	ELOVL fatty acid elongase 4	NM_001099050	7.2	8.4	5.5	1.0E-07
TFPI2	tissue factor pathway inhibitor 2	NM_182788	7.1	7.6	4.8	2.3E-08
PIP4K2A	phosphatidylinositol-5-phosphate 4-kinase, type II, alpha	NM_001192769	7.1	7.8	4.9	3.8E-07
C16H1orf21	chromosome 16 open reading frame, human C1orf21	NM_001081547	7.1	9.1	6.3	5.4E-09
LOC515676	uncharacterized LOC515676	XM_002695644 /// XM_593741	7.0	8.3	5.5	2.5E-04
PMEPA1	prostate transmembrane protein, androgen induced 1	NM_001078078	7.0	7.1	4.3	5.1E-09

NT5E	5'-nucleotidase, ecto (CD73)	NM_174129	7.0	9.0	6.2	5.7E-09
TOX4	TOX high mobility group box family member 4	NM_001075707	7.0	8.0	5.2	3.9E-10
BMP4	bone morphogenetic protein 4	NM_001045877	7.0	7.9	5.1	4.3E-06
RRAS	related RAS viral (r-ras) oncogene homolog	NM_001038688	7.0	9.3	6.5	1.5E-11
G0S2	G0/G1switch 2	NM_001192147	6.9	10.9	8.1	3.4E-07
DSG2	desmoglein 2	NM_001192172	6.9	8.2	5.4	3.2E-06
FBXO33	F-box protein 33	NM_001205749	6.9	8.3	5.6	8.8E-11
SH3KBP1	SH3-domain kinase binding protein 1	NM_001128500	6.8	8.3	5.5	2.8E-09
SERPINB8	serpin peptidase inhibitor, clade B (ovalbumin), member 8	NM_001035287	6.8	8.5	5.7	2.1E-10
RARRES1	retinoic acid receptor responder (tazarotene induced) 1	NM_001075430	6.8	7.7	4.9	7.3E-10
LOC100847427	uncharacterized LOC100847427	XR_138617 /// XR_139213	6.8	10.5	7.7	2.8E-11
P4HA3	prolyl 4-hydroxylase, alpha polypeptide III	NM_001001598	6.8	9.1	6.3	6.1E-09
PLD1	phospholipase D1, phosphatidylcholine-specific	NM_001102001	6.8	8.9	6.1	5.5E-14
PTGFR	prostaglandin F receptor (FP)	NM_181025	6.8	6.4	3.6	1.2E-07
ANXA3	annexin A3	NM_001035325	6.7	8.4	5.6	6.0E-08
LOC790042 /// WARS	Tryptophanyl-tRNA synthetase, cytoplasmic-like /// tryptophanyl-tRNA synthetase	NM_174218 /// XM_001256626	6.7	8.3	5.5	1.0E-09
COBLL1	COBL-like 1	XM_002685374 /// XM_610577	6.7	8.3	5.5	1.3E-07
BT.106027 /// LOC783653	--- /// ---	---	6.7	7.0	4.2	1.7E-10
HIVEP2	human immunodeficiency virus type I enhancer binding protein 2	XM_002690289 /// XM_593747	6.7	8.3	5.5	8.3E-08
WBSCR22	Williams Beuren syndrome chromosome region 22	NM_001034457	6.7	11.9	9.2	1.0E-08
TAX1BP3	Tax1 (human T-cell leukemia virus type I) binding protein 3	NM_001034474	6.7	11.1	8.4	6.9E-12
CUL1	cullin 1	NM_001193233	6.6	9.4	6.6	4.7E-08
SVIL	supervillin	NM_174190	6.6	8.9	6.2	1.9E-09
DPYSL3	dihydropyrimidinase-like 3	NM_001101068	6.6	9.6	6.8	6.3E-07
OCIAD2	OCIA domain containing 2	NM_001034258	6.5	8.5	5.8	1.9E-10
C7H5orf24	chromosome 7 open reading frame, human C5orf24	NM_001076110	6.5	7.4	4.7	7.1E-10
USP4	ubiquitin specific peptidase 4 (proto-oncogene)	NM_001100319	6.5	7.3	4.6	1.1E-10
SPRY2	sprouty homolog 2 (Drosophila)	NM_001076147	6.5	8.9	6.2	2.3E-09

TOPORS	topoisomerase I binding, arginine/serine-rich, E3 ubiquitin protein ligase	NM_001192578	6.5	7.7	5.0	8.0E-09
CHD4	chromodomain helicase DNA binding protein 4	NM_001206501	6.5	8.1	5.4	1.3E-14
FLRT2	leucine-rich repeat transmembrane protein FLRT2-like	XM_003582749 /// XM_003586603	6.5	11.0	8.3	5.0E-09
XRN2	5'-3' exoribonuclease 2	NM_001192472	6.5	8.5	5.8	3.0E-08
ITGAV	integrin, alpha V (vitronectin receptor, alpha polypeptide, antigen CD51)	NM_174367	6.4	11.5	8.8	3.1E-08
CEBPD	CCAAT/enhancer binding protein (C/EBP), delta	NM_174267	6.4	11.0	8.4	1.0E-07
PNMA2	paraneoplastic antigen MA2	NM_001046473	6.4	7.3	4.7	1.7E-09
LEPREL1	leprecan-like 1	NM_001100345	6.3	8.4	5.7	1.4E-06
NR2F1	nuclear receptor subfamily 2, group F, member 1	NM_175804	6.3	9.2	6.5	2.4E-07
TP53INP1	tumor protein p53 inducible nuclear protein 1	NM_001205423	6.3	10.3	7.7	2.3E-07
SETD7	SET domain containing (lysine methyltransferase) 7	XM_002694368 /// XM_594035	6.3	10.4	7.7	9.4E-09
CYR61	cysteine-rich, angiogenic inducer, 61	NM_001034340	6.3	10.4	7.8	1.7E-05
ID3	inhibitor of DNA binding 3, dominant negative helix-loop-helix protein	NM_001014950	6.3	10.0	7.3	4.7E-10
ECD	ecdysoneless homolog (Drosophila)	NM_001046271	6.2	7.6	4.9	2.3E-08
HSD11B1	hydroxysteroid (11-beta) dehydrogenase 1	NM_001123032	6.2	8.8	6.1	9.0E-09
DNASE1L1	deoxyribonuclease I-like 1	NM_001038634	6.2	7.7	5.0	2.0E-11
PAG11	pregnancy-associated glycoprotein 11	NM_176623 /// XM_003584249	6.2	8.6	6.0	2.6E-04
SON	SON DNA binding protein	NM_001083694	6.2	9.8	7.1	2.9E-07
LHFPL2	lipoma HMGIC fusion partner-like 2	NM_001099151	6.2	8.8	6.1	1.9E-07
KDM3A	lysine (K)-specific demethylase 3A	NM_001192872	6.1	9.0	6.3	2.0E-07
CSTB	cystatin B (stefin B)	NM_001037461	6.1	12.0	9.4	6.1E-09
RAB7A	RAB7A, member RAS oncogene family	NM_001035081	6.1	10.0	7.4	2.1E-09
CCDC80	coiled-coil domain containing 80	NM_001098982	6.0	11.0	8.4	7.6E-08
PRPF40A	PRP40 pre-mRNA processing factor 40 homolog A (S. cerevisiae)	XM_002685353 /// XM_003584917 /// XM_003584918 /// XM_003585746	6.0	9.7	7.1	4.2E-06

ANTXR2	anthrax toxin receptor 2	NM_001076826	6.0	8.8	6.2	4.0E-08
B2M /// B2M	beta-2-microglobulin /// beta-2-microglobulin-like	NM_173893 /// XM_001251107 /// XM_002691119	6.0	12.7	10.1	1.8E-08
TCF4	transcription factor 4	NM_001034621	6.0	7.9	5.3	4.0E-08
FLNB	filamin B, beta	NM_001191460	6.0	9.8	7.3	2.2E-09
BOLA	MHC class I heavy chain	NM_001038518 /// NM_001040532	5.9	12.3	9.7	2.9E-04
MAP2	microtubule-associated protein 2	NM_001205807	5.9	9.4	6.8	1.1E-07
SAT1	spermidine/spermine N1-acetyltransferase 1	NM_001034333	5.9	11.6	9.0	1.8E-04
PID1	phosphotyrosine interaction domain containing 1	NM_001079584	5.9	7.5	4.9	3.1E-07
PIK3R1	phosphoinositide-3-kinase, regulatory subunit 1 (alpha)	NM_174575	5.9	8.7	6.1	1.4E-07
OSTF1	osteoclast stimulating factor 1	NM_174409	5.9	10.4	7.8	1.3E-09
S100A8	S100 calcium binding protein A8	NM_001113725	5.9	8.7	6.1	4.7E-06
SH3GLB1	SH3-domain GRB2-like endophilin B1	NM_001077993	5.9	11.1	8.6	4.2E-10
THBS1	thrombospondin 1	NM_174196	5.9	12.9	10.4	6.6E-05
TES	testis derived transcript (3 LIM domains)	NM_001046390	5.8	9.2	6.6	5.6E-09
ARHGEF6	Rac/Cdc42 guanine nucleotide exchange factor (GEF) 6	NM_001193000	5.8	8.0	5.5	2.9E-12
PSAT1	phosphoserine aminotransferase 1	NM_001102150	5.8	9.5	6.9	5.3E-09
BAZ1A	bromodomain adjacent to zinc finger domain, 1A	NM_001192940	5.8	9.3	6.7	6.0E-07
CBLB	Cas-Br-M (murine) ecotropic retroviral transforming sequence b	NM_001205923	5.8	9.4	6.8	6.5E-09
FILIP1L	filamin A interacting protein 1-like	XM_002684706 /// XM_002684707 /// XM_002702383 /// XM_003581681 /// XM_003581682 /// XM	5.8	8.0	5.4	2.2E-08
RSRC2	arginine/serine-rich coiled-coil 2	NM_001102146	5.8	9.2	6.7	1.1E-05
ARPC1B	actin related protein 2/3 complex, subunit 1B, 41kDa	NM_001014844	5.8	8.7	6.2	1.6E-09
C5H12orf75	chromosome 5 open reading frame, human C12orf75	NM_001145202	5.8	9.1	6.6	1.4E-10
ACTA2 /// ACTG2	actin, alpha 2, smooth muscle, aorta /// actin, gamma 2, smooth muscle, enteric	NM_001013592 /// NM_001034502	5.8	9.2	6.7	6.0E-06

AP1S2	adaptor-related protein complex 1, sigma 2 subunit	NM_001040591	5.8	10.5	8.0	6.4E-09
TMEM176A	transmembrane protein 176A	NM_205779	5.8	10.6	8.1	2.2E-09
RALB	v-ral simian leukemia viral oncogene homolog B (ras related; GTP binding protein)	NM_001097985	5.7	9.1	6.6	7.5E-08
MAPK6	mitogen-activated protein kinase 6	XM_002690965 /// XM_002706554	5.7	8.4	5.9	2.1E-07
STRA6	stimulated by retinoic acid gene 6 homolog (mouse)	NM_001075730	5.7	9.2	6.6	2.8E-04
CD68	CD68 molecule	NM_001045902	5.7	8.1	5.6	1.9E-08
LOC100847108	uncharacterized LOC100847108	XR_138612 /// XR_139205	5.7	10.4	7.9	9.8E-08
PYGL	phosphorylase, glycogen, liver	NM_001075203	5.7	10.1	7.6	7.4E-10
STK17A	serine/threonine kinase 17a	NM_001083422	5.7	8.8	6.3	8.5E-08
CHD1	chromodomain helicase DNA binding protein 1	NM_001192048	5.6	9.1	6.6	1.2E-08
TAGLN2	transgelin 2	NM_001013599	5.6	9.9	7.4	1.2E-09
TC2N	tandem C2 domains, nuclear	NM_001193204	5.6	8.1	5.6	1.9E-05
LOC100848773	uncharacterized LOC100848773	XR_139543	5.6	10.4	7.9	1.9E-07
LOC526200	absent in melanoma 1 protein-like	XM_003582606 /// XM_003586468	5.6	6.8	4.4	2.5E-07
ATP6V1A	ATPase, H+ transporting, lysosomal 70kDa, V1 subunit A	NM_174504	5.6	10.0	7.5	5.0E-07
GNB4	guanine nucleotide binding protein (G protein), beta polypeptide 4	NM_001099033	5.6	6.4	3.9	3.1E-09
ZBTB33	zinc finger and BTB domain containing 33	NM_001098157	5.6	7.4	5.0	8.1E-09
KLHL28	kelch-like 28 (Drosophila)	NM_001099030	5.6	9.4	7.0	3.2E-10
HELZ	helicase with zinc finger	NM_001076166 /// XM_001789612 /// XM_002696228	5.6	7.1	4.6	1.4E-11
TRAF3IP2	TRAF3 interacting protein 2	NM_001035483	5.6	8.5	6.0	3.9E-09
DACT1	dapper, antagonist of beta-catenin, homolog 1 (Xenopus laevis)	XM_002690959 /// XM_002690960 /// XM_002700646	5.5	8.3	5.9	8.7E-08
IQGAP1	IQ motif containing GTPase activating protein 1	NM_001191167 /// XM_002696571 /// XM_003583734	5.5	7.9	5.5	9.7E-10

CLIC4	chloride intracellular channel 4	NM_001080218	5.5	10.7	8.2	4.6E-09
TLE1	transducin-like enhancer of split 1 (E(sp1) homolog, Drosophila)	NM_001098020	5.5	8.9	6.4	4.2E-08
TNFAIP8L3	tumor necrosis factor, alpha-induced protein 8-like 3	XM_002690926 /// XM_601426	5.5	9.5	7.0	2.2E-09
CXCL12	chemokine (C-X-C motif) ligand 12	NM_001113174	5.5	7.9	5.4	3.8E-09
GABARAPL1	GABA(A) receptor-associated protein like 1	NM_001033616	5.5	11.8	9.3	2.7E-07
ROCK2	Rho-associated, coiled-coil containing protein kinase 2	NM_174452	5.5	8.6	6.1	4.4E-06
TOP1	topoisomerase (DNA) I	NM_001206487	5.4	8.6	6.2	8.9E-08
NEDD4	neural precursor cell expressed, developmentally down-regulated 4	XM_002690890 /// XM_003582720 /// XM_003586572 /// XM_584456	5.4	8.4	5.9	1.2E-07
RBM25	RNA binding motif protein 25	XM_002690998 /// XM_879644	5.4	6.6	4.1	1.0E-08
MPP5	membrane protein, palmitoylated 5 (MAGUK p55 subfamily member 5)	NM_001205951	5.4	8.3	5.8	3.8E-06
TGFB2	transforming growth factor, beta 2	NM_001113252	5.4	6.6	4.1	2.4E-08
EIF2C2	eukaryotic translation initiation factor 2C, 2	NM_205794	5.4	7.8	5.4	4.0E-08
MTA3	metastasis associated 1 family, member 3	NM_001101964	5.3	8.5	6.1	2.1E-07
MXI1	MAX interactor 1	NM_001076331	5.3	9.9	7.5	3.8E-08
PVRL2	poliovirus receptor-related 2 (herpesvirus entry mediator B)	NM_001075210	5.3	8.1	5.7	2.1E-07
C1S	complement component 1, s subcomponent	NM_001076550	5.3	7.6	5.2	5.6E-07
FERMT2	fermitin family member 2	NM_001101264	5.3	9.9	7.5	1.7E-07
SELM	selenoprotein M	NM_001163171	5.3	9.2	6.8	1.9E-07
RNF8	ring finger protein 8	NM_001046216	5.3	8.2	5.8	3.5E-07
PARD3	par-3 partitioning defective 3 homolog (C. elegans)	XM_002692115 /// XM_606371	5.3	8.5	6.1	8.8E-11
ODF2L	outer dense fiber of sperm tails 2-like	NM_001075993	5.3	9.6	7.2	1.3E-08
REXO2	REX2, RNA exonuclease 2 homolog (S. cerevisiae)	NM_001081735	5.3	11.5	9.1	1.4E-08
LOC100850718	uncharacterized LOC100850718	XR_139132	5.3	7.6	5.2	1.5E-07
ASCC2	activating signal cointegrator 1 complex subunit 2	NM_001015524	5.3	8.8	6.4	9.7E-10
CHD2	chromodomain helicase DNA binding protein 2	NM_001102181	5.3	7.9	5.5	2.0E-10

SAFB2	scaffold attachment factor B2	NM_001256554 /// XM_002684211 /// XM_002688890	5.2	8.0	5.6	4.7E-06
CD83	CD83 molecule	NM_001046590	5.2	7.7	5.3	4.6E-07
MXRA8	matrix-remodelling associated 8	NM_001075830	5.2	11.1	8.8	1.2E-04
TIMP2	TIMP metallopeptidase inhibitor 2	NM_174472	5.2	12.1	9.7	1.4E-07
COL12A1	collagen, type XII, alpha 1	NM_001206497	5.2	8.0	5.6	5.5E-09
PHF20L1	PHD finger protein 20-like 1	NM_001082432	5.2	7.5	5.1	2.6E-08
ROCK1	Rho-associated, coiled-coil containing protein kinase 1	NM_001191227 /// XM_002697789 /// XM_003583969	5.2	8.5	6.1	1.1E-05
KCTD10	potassium channel tetramerisation domain containing 10	NM_001101216	5.2	10.8	8.4	8.5E-10
MMP23B	matrix metallopeptidase 23B	NM_001038556	5.2	9.0	6.6	2.9E-11
BAMBI	BMP and activin membrane-bound inhibitor homolog (Xenopus laevis)	NM_001046309	5.1	8.0	5.6	4.1E-06
GNAI1	guanine nucleotide binding protein (G protein), alpha inhibiting activity polypeptide 1	NM_174324	5.1	11.0	8.7	1.7E-10
CAPN2	calpain 2, (m/II) large subunit	NM_001103086	5.1	11.0	8.6	9.1E-10
CNN1	calponin 1, basic, smooth muscle	NM_001046379	5.1	8.2	5.9	8.5E-10
TMOD3	tropomodulin 3 (ubiquitous)	NM_001075987	5.1	8.4	6.0	1.5E-07
ANKRD11	ankyrin repeat domain 11	XM_002694765 /// XM_612059	5.1	8.4	6.0	5.9E-09
CHD2 /// LOC100847962	chromodomain helicase DNA binding protein 2 /// chromodomain-helicase-DNA-binding prote	NM_001102181 /// XM_003583720 /// XM_003587543	5.1	8.0	5.7	1.4E-08
PLAUR	plasminogen activator, urokinase receptor	NM_174423	5.1	8.1	5.7	1.9E-10
ISG20	interferon stimulated exonuclease gene 20kDa	XM_002696514 /// XM_583075	5.1	7.5	5.2	6.0E-09
BZW1	basic leucine zipper and W2 domains 1	NM_001206613	5.1	9.4	7.0	1.1E-05
FBXO32	F-box protein 32	NM_001046155	5.1	8.6	6.2	6.0E-07
SULT1A1	sulfotransferase family, cytosolic, 1A, phenol-preferring, member 1	NM_177521	5.1	9.1	6.8	3.4E-11
HECA	headcase homolog (Drosophila)	XM_001255659 /// XM_002690282	5.1	10.4	8.1	5.2E-09

GNPTAB	N-acetylglucosamine-1-phosphate transferase, alpha and beta subunits	NM_001192228	5.1	9.7	7.4	3.9E-08
CMTM3	CKLF-like MARVEL transmembrane domain containing 3	NM_001099399	5.0	10.9	8.6	1.8E-09
ZHX2	zinc fingers and homeoboxes 2	NM_001206406	5.0	8.6	6.3	2.3E-08
MEGF9	multiple epidermal growth factor-like domains protein 9-like	XM_003582585 /// XM_003586445	5.0	7.6	5.3	3.2E-07
IRF9	interferon regulatory factor 9	NM_001024506	5.0	8.1	5.7	1.1E-08
ARMCX3	armadillo repeat containing, X-linked 3	NM_001192453	5.0	8.9	6.6	2.1E-08
CUX1	cut-like homeobox 1	XM_003587841 /// XM_601985	5.0	7.5	5.2	6.4E-11
PDLIM1	PDZ and LIM domain 1	NM_001035455	5.0	10.7	8.4	2.7E-07
TPD52L1	tumor protein D52-like 1	NM_001076033	5.0	7.3	5.0	3.1E-05
RBM5	RNA binding motif protein 5	NM_001046374	5.0	7.4	5.1	3.0E-10
NGF	nerve growth factor (beta polypeptide)	NM_001099362	5.0	7.6	5.2	3.3E-08
FAM43A	family with sequence similarity 43, member A	XM_002684829 /// XM_586734	4.9	7.7	5.4	1.7E-06
SBDS	Shwachman-Bodian-Diamond syndrome	NM_001034439	4.9	9.3	7.0	6.1E-07
ENPP5	ectonucleotide pyrophosphatase/phosphodiesterase 5 (putative)	NM_001206724	4.9	7.7	5.4	2.5E-06
TAP1	transporter 1, ATP-binding cassette, sub-family B (MDR/TAP)	NM_001098058	4.9	9.1	6.8	3.7E-07
ZNF24	zinc finger protein 24	NM_001046445 /// NM_001205479	4.9	8.6	6.3	2.6E-08
STAT1 /// STAT4	signal transducer and activator of transcription 1, 91kDa /// signal transducer and act	NM_001077900 /// NM_001083692 /// XM_001787142 /// XM_002685461	4.9	8.0	5.7	2.9E-08
RNF144B	ring finger protein 144B	NM_001099028	4.9	7.5	5.2	1.5E-06
PDHA1	pyruvate dehydrogenase (lipoamide) alpha 1	NM_001101046	4.9	5.9	3.6	4.7E-12
STK10	serine/threonine kinase 10	NM_001192627	4.9	8.9	6.6	1.1E-07
CLINT1	clathrin interactor 1	NM_001105417	4.9	7.6	5.3	5.6E-09
MYCBP2	MYC binding protein 2	NM_001192817	4.9	9.9	7.6	3.4E-10
CD302	CD302 molecule	NM_001110191	4.9	7.0	4.8	1.5E-08

TSC22D1	TSC22 domain family, member 1	NM_001034377	4.9	13.2	10.9	2.4E-07
UHRF1BP1L	UHRF1 binding protein 1-like	NM_001192527	4.9	8.2	5.9	6.3E-08
SEMA6D	sema domain, transmembrane domain (TM), and cytoplasmic domain, (semaphorin) 6D	NM_001191133	4.9	9.9	7.6	2.4E-05
LRCH1	leucine-rich repeats and calponin homology (CH) domain containing 1	XM_002691830 /// XM_581598	4.9	6.6	4.3	3.6E-08
EIF4H	eukaryotic translation initiation factor 4H	NM_001075752	4.8	8.9	6.6	6.1E-04
ADAM12	ADAM metallopeptidase domain 12	NM_001001156	4.8	7.7	5.4	1.7E-06
CAV1	caveolin 1, caveolae protein, 22kDa	NM_174004	4.8	6.9	4.6	1.3E-08
SERTAD1	SERTA domain containing 1	NM_001040564	4.8	9.1	6.8	1.4E-09
SPTBN1	spectrin, beta, non-erythrocytic 1	NM_001192276	4.8	8.3	6.0	1.7E-08
AIDA	axin interactor, dorsalization associated	NM_001205634	4.8	8.1	5.8	3.8E-07
SPARC	secreted protein, acidic, cysteine-rich (osteonectin)	NM_174464	4.8	12.1	9.8	5.2E-11
RILPL1	Rab interacting lysosomal protein-like 1	NM_001075226	4.8	9.8	7.5	1.1E-08
PPAP2C	phosphatidic acid phosphatase type 2C	NM_001045890	4.8	8.1	5.8	1.7E-10
SDE2	SDE2 telomere maintenance homolog (S. pombe)	NM_001099065	4.8	7.4	5.1	7.5E-08
KCNMB4	potassium large conductance calcium-activated channel, subfamily M, beta member 4	NM_001192523	4.8	7.5	5.3	1.5E-08
WHSC1L1	Wolf-Hirschhorn syndrome candidate 1-like 1	NM_001075595 /// XM_003584171 /// XM_003584172 /// XM_003587971 /// XM_003587972	4.8	8.9	6.6	3.2E-08
NUDT10 /// NUDT11	nudix (nucleoside diphosphate linked moiety X)-type motif 10 /// nudix (nucleoside diph	NM_001035488 /// NM_001101296	4.8	10.5	8.3	1.4E-05
CCL3	chemokine (C-C motif) ligand 3	NM_174511	4.8	7.6	5.3	1.6E-06
ZNF398	zinc finger protein 398	NM_001205918	4.8	8.3	6.1	1.3E-09
SLC31A2	solute carrier family 31 (copper transporters), member 2	NM_001034556	4.8	9.5	7.3	6.1E-08
C4H7orf41	chromosome 4 open reading frame, human C7orf41	NM_001101246	4.7	8.7	6.5	2.7E-07
EFNA5	ephrin-A5	NM_001076432	4.7	9.8	7.5	7.9E-05
CXCL10	chemokine (C-X-C motif) ligand 10	NM_001046551	4.7	6.9	4.6	1.6E-04
SH3BP5	SH3-domain binding protein 5 (BTK-associated)	NM_001206288	4.7	7.9	5.7	6.4E-10

SMARCA4	SWI/SNF related, matrix associated, actin dependent regulator of chromatin, subfamily a	NM_001105614	4.7	9.4	7.2	2.5E-08
LOC100335559 /// SQSTM1	sequestosome-1-like /// sequestosome 1	NM_176641 /// XM_002704190	4.7	12.3	10.1	1.7E-09
PDLIM7	PDZ and LIM domain 7 (enigma)	NM_001017947 /// NM_001113251	4.7	7.0	4.8	8.3E-11
WARS	tryptophanyl-tRNA synthetase	NM_174218	4.7	9.9	7.7	1.4E-08
UTP6	UTP6, small subunit (SSU) processome component, homolog (yeast)	NM_001099140	4.7	8.9	6.7	8.7E-06
JPH1	junctophilin 1	NM_001192887	4.7	7.0	4.8	1.4E-06
KANK2	KN motif and ankyrin repeat domains 2	NM_001076531	4.6	9.0	6.8	1.3E-07
INSIG2	insulin induced gene 2	XM_002685428 /// XM_003581843 /// XM_003585754 /// XM_614207	4.6	8.9	6.6	1.9E-09
RAI14	retinoic acid induced 14	XM_002696398 /// XM_604226	4.6	9.7	7.5	2.5E-07
RHOQ	ras homolog gene family, member Q	NM_001205498	4.6	8.2	6.0	2.1E-10
A2M	alpha-2-macroglobulin	NM_001109795	4.6	8.0	5.8	9.0E-05
IFNAR1	interferon (alpha, beta and omega) receptor 1	NM_174552	4.6	7.1	4.9	7.9E-14
PHF3	PHD finger protein 3	NM_001192571	4.5	6.6	4.4	1.9E-10
CUL3	cullin 3	NM_001192806	4.5	8.2	6.0	3.3E-06
PRPF18	PRP18 pre-mRNA processing factor 18 homolog (S. cerevisiae)	NM_001075323	4.5	7.8	5.6	1.0E-08
S100A11	S100 calcium binding protein A11	NM_001098856	4.5	12.4	10.2	3.0E-09
AFAP1	actin filament associated protein 1	NM_001206044	4.5	7.6	5.5	4.8E-07
ITPK1	inositol-tetrakisphosphate 1-kinase	NM_001192489	4.5	9.1	6.9	4.9E-07
FSTL3	follistatin-like 3 (secreted glycoprotein)	NM_001075710	4.5	9.4	7.2	1.3E-09
KLF10	Kruppel-like factor 10	NM_001168462	4.5	9.0	6.9	1.2E-06
SPOCK2	sparc/osteonectin, cwcv and kazal-like domains proteoglycan (testican) 2	NM_001101115	4.5	7.5	5.3	3.8E-04
RNF168	ring finger protein 168, E3 ubiquitin protein ligase	NM_001076289	4.5	8.5	6.3	1.0E-09
MAP7D1	MAP7 domain containing 1	XM_002686551 /// XM_589552	4.5	9.6	7.4	5.2E-09

KDM5A	lysine (K)-specific demethylase 5A	NM_001205625	4.5	8.9	6.7	1.8E-09
NFIL3	nuclear factor, interleukin 3 regulated	NM_001075240	4.5	9.2	7.1	2.0E-07
COL4A1	collagen, type IV, alpha 1	NM_001166511	4.5	9.5	7.3	2.1E-08
EPHA7	EPH receptor A7	NM_001192726	4.5	6.1	3.9	1.4E-11
LOC539199 /// MAMDC2	MAM domain-containing protein 2-like /// MAM domain containing 2	XM_001787385 /// XM_002689639 /// XM_585556	4.4	6.8	4.7	1.7E-06
FBXO7	F-box protein 7	NM_001038059	4.4	10.9	8.7	1.7E-09
YPEL5	yippee-like 5 (Drosophila)	NM_001079793	4.4	10.4	8.2	2.1E-07
SLC20A2	solute carrier family 20 (phosphate transporter), member 2	NM_001080280	4.4	7.9	5.8	9.1E-07
PSAP	prosaposin	NM_174161	4.4	9.3	7.2	8.2E-06
TP53BP2	tumor protein p53 binding protein, 2	XM_002693966 /// XM_002701521	4.4	11.8	9.6	5.2E-09
UBAP2L	ubiquitin associated protein 2-like	NM_001103107	4.4	9.2	7.0	2.5E-09
ZNF281	zinc finger protein 281	XM_002694236 /// XM_610917	4.4	9.6	7.5	3.2E-10
FRMD6 /// LOC100335364	FERM domain containing 6 /// FERM domain-containing protein 6-like	NM_001102133 /// XM_002700610	4.4	7.3	5.2	1.8E-07
PHF6	PHD finger protein 6	NM_001075769	4.4	8.2	6.0	5.0E-06
LOC100851799 /// SLC40A1	solute carrier family 40 member 1-like /// solute carrier family 40 (iron-regulated tra	NM_001077970 /// XM_003581794	4.4	9.5	7.4	1.1E-03
WDR47	WD repeat domain 47	XM_002686156 /// XM_003581964 /// XM_003581965 /// XM_003585856 /// XM_003585857 /// XM	4.4	7.6	5.5	2.2E-09
EFTUD1	elongation factor Tu GTP binding domain containing 1	XM_002696606 /// XM_002696607 /// XM_606001	4.4	8.9	6.8	2.4E-09
HSPA14	heat shock 70kDa protein 14	NM_001046388	4.3	8.2	6.1	2.5E-06
IGF2	insulin-like growth factor 2 (somatomedin A)	NM_174087	4.3	7.9	5.8	9.7E-09
QSOX1	quiescin Q6 sulfhydryl oxidase 1	NM_001102074	4.3	9.3	7.2	4.7E-09

KANK1	KN motif and ankyrin repeat domains 1	XM_002689629 /// XM_614789	4.3	7.6	5.5	3.1E-09
FABP5	fatty acid binding protein 5 (psoriasis-associated)	NM_174315	4.3	7.8	5.7	2.1E-05
FAM114A1	family with sequence similarity 114, member A1	XM_002688202 /// XM_588946	4.3	7.1	4.9	6.3E-05
LOC537017	cytidine monophosphate-N-acetylneuraminic acid hydroxylase-like	XM_002697563 /// XM_617171	4.3	7.1	5.0	1.0E-04
SDC4	syndecan 4	XM_002692330 /// XM_584869	4.3	9.8	7.7	1.2E-09
PRPF38B	PRP38 pre-mRNA processing factor 38 (yeast) domain containing B	XM_002686159 /// XM_003584875	4.3	7.6	5.5	2.1E-05
CPEB4	cytoplasmic polyadenylation element binding protein 4	NM_001105420 /// XM_003583671 /// XM_003583672 /// XM_003587502 /// XM_003587503	4.3	7.4	5.3	2.1E-08
MARCKS	myristoylated alanine-rich protein kinase C substrate	NM_001076276	4.3	8.7	6.6	2.0E-07
SWAP70	SWAP switching B-cell complex 70kDa subunit	NM_001080297	4.3	8.3	6.1	1.0E-07
IL6ST	interleukin 6 signal transducer (gp130, oncostatin M receptor)	XM_002696322 /// XM_600430	4.3	7.4	5.3	2.8E-07
CFB	complement factor B	NM_001040526	4.3	7.6	5.5	1.4E-09
CDC37L1	cell division cycle 37 homolog (S. cerevisiae)-like 1	NM_001098931	4.3	6.4	4.2	8.7E-08
SGSH	N-sulfoglucosamine sulfohydrolase	NM_001102189	4.3	8.2	6.1	1.3E-08
LIMCH1	LIM and calponin homology domains 1	NM_001191521	4.3	7.2	5.1	1.5E-08
ACVR1	activin A receptor, type I	NM_176663	4.3	8.3	6.2	3.2E-09
RASA2	RAS p21 protein activator 2	NM_001193020	4.3	8.2	6.1	3.5E-07
PLEKHB2	pleckstrin homology domain containing, family B (evectins) member 2	NM_001083529	4.3	10.2	8.1	1.9E-09
MICU1	mitochondrial calcium uptake 1	NM_001075338	4.3	9.4	7.3	1.9E-09
STK38L	serine/threonine kinase 38 like	NM_001101092	4.3	11.0	8.9	5.1E-09
SLC25A12	solute carrier family 25 (mitochondrial carrier, Aralar), member 12	NM_001101194	4.3	8.9	6.8	5.0E-08
ATG4A	ATG4 autophagy related 4 homolog A (S. cerevisiae)	NM_001001171	4.3	6.8	4.7	1.8E-07

FSCN1	fascin homolog 1, actin-bundling protein (Strongylocentrotus purpuratus)	NM_001035045	4.3	8.5	6.4	1.1E-09
TMBIM4	transmembrane BAX inhibitor motif containing 4	NM_001014914	4.3	8.9	6.8	1.8E-07
MSTN	myostatin	NM_001001525	4.3	7.4	5.3	1.1E-03
SIRT2	sirtuin 2	NM_001113531	4.3	7.8	5.7	1.2E-11
GLB1	galactosidase, beta 1	NM_001035043	4.3	9.6	7.5	1.5E-09
PLRG1	pleiotropic regulator 1	NM_001046362	4.3	8.0	5.9	2.1E-08
RABGAP1L	RAB GTPase activating protein 1-like	NM_001105427 /// XM_003583255 /// XM_003583256 /// XM_003587088 /// XM_003587089	4.3	6.8	4.7	1.7E-09
VAT1	vesicle amine transport protein 1 homolog (T. californica)	NM_001192265	4.3	9.8	7.8	4.3E-07
CRYZ	crystallin, zeta (quinone reductase)	NM_174025	4.3	9.2	7.1	2.6E-08
FAF2	Fas associated factor family member 2	NM_001077020	4.2	8.4	6.3	3.3E-10
DSC2	desmocollin 2	NM_001166526	4.2	7.4	5.3	1.2E-07
LITAF	lipopolysaccharide-induced TNF factor	NM_001046252	4.2	8.4	6.4	1.8E-07
C10H5orf13	chromosome 10 open reading frame, human C5orf13	NM_001105045	4.2	8.1	6.0	2.4E-07
TNFRSF21	tumor necrosis factor receptor superfamily, member 21	NM_001076911 /// XM_003583868	4.2	8.7	6.6	3.4E-07
ATP6V0D1	ATPase, H+ transporting, lysosomal 38kDa, V0 subunit d1	NM_174505	4.2	11.3	9.2	1.3E-09
HPSE	heparanase	NM_174082	4.2	9.0	7.0	1.2E-03
UFM1	ubiquitin-fold modifier 1	NM_001046314	4.2	8.2	6.1	7.3E-08
RBFOX2	RNA binding protein, fox-1 homolog (C. elegans) 2	NM_001205372	4.2	9.1	7.1	1.3E-08
NUDT12	nudix (nucleoside diphosphate linked moiety X)-type motif 12	NM_001046608	4.2	6.7	4.6	8.6E-10
GOLGA4	golgin A4	NM_001192125	4.2	6.6	4.6	1.2E-07
TADA2A	transcriptional adaptor 2A	NM_001034244	4.2	8.1	6.1	2.0E-07
LMO7	LIM domain 7	NM_001109801 /// XM_003582872 /// XM_003582873 /// XM_003586719 /// XM_003586720	4.2	6.0	4.0	2.9E-09

DUSP1	dual specificity phosphatase 1	NM_001046452	4.2	10.1	8.1	2.4E-06
GDI1	GDP dissociation inhibitor 1	NM_174064	4.2	10.0	8.0	2.2E-05
MAP3K3	mitogen-activated protein kinase kinase kinase 3	XM_002696094 /// XM_880929	4.2	8.5	6.4	4.7E-12
CRIM1	cysteine rich transmembrane BMP regulator 1 (chordin-like)	NM_001205298	4.2	9.6	7.5	4.2E-07
SMN1	survival of motor neuron 1, telomeric	NM_175701	4.2	8.4	6.3	7.2E-06
TUBB6	tubulin, beta 6 class V	NM_001046373	4.2	10.4	8.4	2.9E-07
SF3B1	splicing factor 3b, subunit 1, 155kDa	NM_001192994	4.2	8.3	6.3	5.1E-08
SACS	spastic ataxia of Charlevoix-Saguenay (sacsin)	XM_002691863 /// XM_586753	4.2	9.0	6.9	2.0E-07
GSK3B	glycogen synthase kinase 3 beta	NM_001101310	4.2	8.7	6.6	2.8E-07
CDH11 /// LOC100851861	cadherin 11, type 2, OB-cadherin (osteoblast) /// cadherin-11-like	NM_001081624 /// XR_138938	4.1	9.8	7.7	8.5E-03
MYB	v-myb myeloblastosis viral oncogene homolog (avian)	NM_175050	4.1	6.8	4.7	5.8E-06
CCM2	cerebral cavernous malformation 2	NM_001193211	4.1	9.8	7.7	1.3E-08
GRIA3	glutamate receptor, ionotrophic, AMPA 3	NM_001206059	4.1	7.4	5.4	4.4E-08
DYNLT3	dynein, light chain, Tctex-type 3	NM_001101220	4.1	8.7	6.7	8.7E-06
VPRBP	Vpr (HIV-1) binding protein	XM_002697028 /// XM_003583810	4.1	8.1	6.0	7.0E-06
DSEL	dermatan sulfate epimerase-like	NM_001206461	4.1	9.3	7.3	2.7E-09
DDAH2	dimethylarginine dimethylaminohydrolase 2	NM_001034704	4.1	9.1	7.1	6.5E-09
ING4	inhibitor of growth family, member 4	NM_001035389	4.1	8.2	6.1	1.1E-06
BRE	brain and reproductive organ-expressed (TNFRSF1A modulator)	NM_001102274	4.1	9.0	7.0	9.0E-09
NDRG3	NDRG family member 3	NM_001101996	4.1	10.6	8.5	3.4E-11
VPS26A	vacuolar protein sorting 26 homolog A (S. pombe)	NM_001075455	4.1	9.6	7.6	6.2E-03
TXNIP	thioredoxin interacting protein	NM_001101875	4.1	11.7	9.7	1.8E-06
TBCE	tubulin folding cofactor E	NM_001038032	4.1	8.5	6.5	4.3E-07
PELI1	pellino homolog 1 (Drosophila)	NM_001078004	4.1	9.7	7.7	1.5E-07
IRGQ	immunity-related GTPase family, Q	NM_001193129	4.1	9.6	7.5	7.0E-10
COL11A1	collagen, type XI, alpha 1	NM_001166509	4.1	7.1	5.1	6.1E-07
RNASET2	ribonuclease T2	NM_001206337	4.1	9.2	7.2	1.1E-11

LOC100847604	uncharacterized LOC100847604	XM_003582700 /// XM_003586558	4.1	8.2	6.1	1.0E-06
C3H1orf226	chromosome 3 open reading frame, human C1orf226	NM_001083790	4.1	8.2	6.2	6.3E-09
CDC42SE2	CDC42 small effector 2	NM_001102537	4.1	7.7	5.7	3.8E-07
DEFB4A /// DEFB5 /// LOC100335951	defensin, beta 4A /// defensin, beta 5 /// beta-defensin 4-like	NM_001130761 /// NM_174775 /// XM_002706761	4.1	6.8	4.8	1.6E-04
RBMS2	RNA binding motif, single stranded interacting protein 2	NM_001034365	4.1	7.6	5.6	8.5E-10
ST3GAL5	ST3 beta-galactoside alpha-2,3-sialyltransferase 5	NM_205807	4.1	8.8	6.8	6.6E-08
PLSCR4	phospholipid scramblase 4	NM_001081732	4.1	9.8	7.7	4.3E-07
TACC2	transforming, acidic coiled-coil containing protein 2	NM_001102159 /// XM_003584113 /// XM_003584114 /// XM_003587903	4.0	7.8	5.8	2.6E-08
ZNF462	zinc finger protein 462	NM_001205811	4.0	7.7	5.7	5.1E-09
CPNE8	copine VIII	NM_001098085	4.0	7.5	5.4	3.0E-09
MLLT11	myeloid/lymphoid or mixed-lineage leukemia (trithorax homolog, Drosophila); translocate	NM_001076169	4.0	9.1	7.1	2.8E-06
CRMP1	collapsin response mediator protein 1	NM_001192021	4.0	6.6	4.6	3.2E-07
FAM32A	family with sequence similarity 32, member A	NM_001105004	4.0	9.3	7.3	1.4E-08
HMBOX1	homeobox containing 1	NM_001192171	4.0	9.0	7.0	4.0E-08
CDO1	cysteine dioxygenase, type I	NM_001034465	4.0	10.2	8.2	1.2E-05
C5H22orf28	chromosome 5 open reading frame, human C22orf28	NM_001015631	4.0	8.9	6.9	5.4E-08
FBN1	fibrillin 1	NM_174053	4.0	9.5	7.5	1.2E-06
VMP1	vacuole membrane protein 1	NM_001075368	4.0	11.2	9.2	2.4E-08
LRRFIP1	leucine rich repeat (in FLII) interacting protein 1	NM_001102308	4.0	7.4	5.4	6.9E-06
LOC100337023	collagen alpha-1(V) chain-like	XR_139433	4.0	9.5	7.5	4.6E-08
RALA	v-ral simian leukemia viral oncogene homolog A (ras related)	NM_001034644	4.0	8.7	6.7	1.8E-06
C7H1orf35	chromosome 7 open reading frame, human C1orf35	NM_001034272	4.0	9.0	7.0	2.4E-08
APOB	---	---	-4.0	5.2	7.2	4.4E-08
ATP10B	ATPase, class V, type 10B	XM_002689352 /// XM_003582473	-4.0	4.4	6.4	6.3E-07

ALG3	asparagine-linked glycosylation 3, alpha-1,3-mannosyltransferase homolog (S. cerevisia	NM_001083511	-4.0	5.6	7.6	1.7E-11
LRRC45	leucine rich repeat containing 45	NM_001191278 /// XM_002696135 /// XM_003583635	-4.0	5.9	7.9	3.9E-07
ALB	albumin	NM_180992	-4.0	4.3	6.3	4.0E-08
CNTROB	centrobin, centrosomal BRCA2 interacting protein	XM_002695837 /// XM_584868	-4.0	7.3	9.3	2.0E-09
CATHL1 /// LOC100847261	cathelicidin 1 /// cathelicidin-1-like	NM_174825 /// XM_003587646	-4.1	4.5	6.5	1.7E-07
CD72	CD72 molecule	XM_001251357 /// XM_002689678	-4.1	4.9	6.9	5.8E-09
CDH26	cadherin 26	XM_002692262 /// XM_869285	-4.1	5.7	7.7	2.0E-08
IL6R	interleukin 6 receptor	NM_001110785	-4.1	5.7	7.7	3.7E-07
NABP1	oligonucleotide/oligosaccharide-binding fold containing 2A	NM_001098124	-4.1	5.4	7.4	2.1E-09
FAM134C	family with sequence similarity 134, member C	XM_001255597 /// XM_002695994	-4.1	7.3	9.3	1.2E-07
FAM134C	family with sequence similarity 134, member C	XM_001255597 /// XM_002695994	-4.1	5.9	8.0	4.8E-07
YBX2	Y box binding protein 2	NM_001098126	-4.1	5.0	7.0	1.1E-07
F2RL2	coagulation factor II (thrombin) receptor-like 2	NM_001038533	-4.1	5.2	7.3	1.1E-06
HEG1	HEG homolog 1 (zebrafish)	XM_002684814 /// XM_589074	-4.2	7.7	9.8	4.8E-04
HOXB2	homeobox B2	NM_001191335	-4.2	5.2	7.3	8.8E-09
EMID1	EMI domain containing 1	NM_001101867	-4.2	9.1	11.2	1.2E-03
TRIB2	tribbles homolog 2 (Drosophila)	NM_178317	-4.2	10.2	12.3	1.6E-05
PLP1	proteolipid protein 1	NM_174149	-4.3	4.5	6.6	3.7E-08
LOC782061	aldo-keto reductase family 1, member C1-like	NM_001166223	-4.3	4.6	6.7	1.8E-07
DHRS3	dehydrogenase/reductase (SDR family) member 3	NM_174180	-4.3	8.3	10.4	9.0E-05
LGALS4	lectin, galactoside-binding, soluble, 4	NM_001034768	-4.3	6.2	8.3	8.0E-07
HNRNPL	heterogeneous nuclear ribonucleoprotein L	NM_001192030	-4.3	5.0	7.1	3.2E-10
GIMAP7	GTPase, IMAP family member 7	NM_001080257	-4.3	5.5	7.6	1.7E-07

LOC100300716 /// LOC100300806	uncharacterized LOC100300716 /// uncharacterized LOC100300806	XM_002683998 /// XM_003584833 /// XM_003585559	-4.3	4.7	6.8	2.2E-06
SLCO2B1	solute carrier organic anion transporter family, member 2B1	NM_174843	-4.3	5.0	7.1	1.3E-09
LRRC17	leucine rich repeat containing 17	NM_001078150	-4.3	6.9	9.0	1.1E-04
LAT	linker for activation of T cells	NM_001104978	-4.3	5.7	7.8	1.6E-08
EXOC3L4	exocyst complex component 3-like 4	NM_001205930	-4.4	6.2	8.3	9.0E-06
SELL	selectin L	NM_174182	-4.4	5.2	7.3	1.0E-08
TCFL5	transcription factor-like 5 (basic helix-loop-helix)	XM_003582947 /// XM_003586831	-4.4	5.7	7.8	2.2E-07
ZP2	zona pellucida glycoprotein 2 (sperm receptor)	NM_173973	-4.4	4.4	6.5	9.2E-06
GCLC	glutamate-cysteine ligase, catalytic subunit	NM_001083674	-4.4	7.7	9.8	5.3E-05
BTN3A3	butyrophilin, subfamily 3, member A3	NM_001034207	-4.4	4.3	6.5	2.9E-07
CCL25	chemokine (C-C motif) ligand 25	NM_001046569	-4.4	6.8	8.9	7.0E-07
IGL@ /// IGLL1	immunoglobulin light chain, lambda gene cluster /// immunoglobulin lambda-like polypeptide 1	NM_001075204 /// NM_001083800	-4.4	5.7	7.8	9.2E-10
ACTA1	actin, alpha 1, skeletal muscle	NM_174225	-4.5	6.5	8.6	5.0E-05
ACSM1	acyl-CoA synthetase medium-chain family member 1	NM_174682	-4.5	4.2	6.4	7.5E-08
TMIGD2	transmembrane and immunoglobulin domain containing 2	XM_002688933 /// XM_602676	-4.5	5.0	7.2	9.8E-08
RPRD1A	regulation of nuclear pre-mRNA domain containing 1A	NM_001075156	-4.5	5.8	8.0	9.9E-08
ZP3	zona pellucida glycoprotein 3 (sperm receptor)	NM_173974	-4.6	5.3	7.5	2.1E-09
CENPE	centromere protein E, 312kDa	XM_002688095 /// XM_002704516	-4.6	6.0	8.2	1.0E-06
CMBL	carboxymethylenebutenolidase homolog (Pseudomonas)	NM_001192983	-4.6	9.9	12.1	6.4E-05
RHBG	Rh family, B glycoprotein	NM_174723	-4.6	4.8	7.0	1.3E-09
ELMO1	engulfment and cell motility 1	NM_001024505 /// NM_001113227	-4.6	4.7	6.9	7.8E-08
ATP5SL	ATP5S-like	NM_001272008 /// NM_001272009 /// XM_003583433 /// XM_003587269	-4.7	5.1	7.3	3.7E-07
SLC35G1	solute carrier family 35, member G1	NM_001076470	-4.7	7.8	10.0	6.6E-04

NUDT16	nudix (nucleoside diphosphate linked moiety X)-type motif 16	NM_001075560	-4.7	6.8	9.0	1.1E-09
DMGDH	dimethylglycine dehydrogenase	NM_001205545	-4.7	4.6	6.8	9.7E-12
AP3B2	adaptor-related protein complex 3, beta 2 subunit	NM_001243335	-4.8	6.2	8.5	8.7E-05
DDO	D-aspartate oxidase	NM_173908	-4.8	4.2	6.4	1.2E-04
TTYH1	tweety homolog 1 (Drosophila)	NM_001077015	-4.9	6.1	8.4	6.8E-08
LOC505468	cytochrome P450 family 2 subfamily C polypeptide 18-like	XM_002698396 /// XM_002698397 /// XM_002703179 /// XM_003584074 /// XM_003587881 /// XM	-4.9	4.2	6.5	6.2E-09
MZB1	marginal zone B and B1 cell-specific protein	NM_001098930	-4.9	6.6	8.9	3.5E-08
ITGA6	integrin, alpha 6	NM_001109981	-5.0	9.5	11.8	3.5E-08
TCRA	T cell receptor, alpha	NM_001075519 /// NM_001098474	-5.0	6.7	9.0	2.9E-08
LOC100297608	uncharacterized LOC100297608	XM_002694589 /// XM_002701727	-5.0	4.2	6.6	1.2E-06
CCDC3	coiled-coil domain containing 3	NM_001172375	-5.0	9.4	11.8	1.3E-08
SORL1	sortilin-related receptor, L(DLR class) A repeats containing	NM_001192757	-5.1	7.2	9.5	7.4E-06
EFHD1	EF-hand domain family, member D1	NM_001075832	-5.1	6.4	8.8	5.0E-05
IQGAP2	IQ motif containing GTPase activating protein 2	XM_002690436 /// XM_003582648	-5.1	7.1	9.4	3.0E-09
PKDCC	protein kinase domain containing, cytoplasmic homolog (mouse)	NM_001206721	-5.1	6.8	9.1	9.7E-10
TOP2A	topoisomerase (DNA) II alpha 170kDa	XM_001254457 /// XM_002696009	-5.2	8.4	10.8	4.2E-05
AOAH	acyloxyacyl hydrolase (neutrophil)	NM_001078096	-5.2	6.5	8.9	8.1E-09
PGF	placental growth factor	NM_173950	-5.2	6.7	9.1	2.2E-04
AQP1	aquaporin 1 (Colton blood group)	NM_174702	-5.4	5.2	7.6	3.5E-08
MOB3B	MOB kinase activator 3B	NM_001046491	-5.4	6.3	8.8	4.1E-07
RAC3	ras-related C3 botulinum toxin substrate 3 (rho family, small GTP binding protein Rac3)	NM_001099179	-5.5	5.6	8.0	2.4E-06
PABPN1	poly(A) binding protein, nuclear 1	NM_174569	-5.5	7.4	9.9	2.1E-09

JAKMIP1	janus kinase and microtubule interacting protein 1	NM_001102251	-5.7	6.6	9.1	6.0E-05
HAUS4	HAUS augmin-like complex, subunit 4	NM_001206190	-5.7	5.4	7.9	1.5E-08
NOS2	nitric oxide synthase 2, inducible	NM_001076799	-5.8	9.4	11.9	7.1E-07
SLC10A2	solute carrier family 10 (sodium/bile acid cotransporter family), member 2	XM_002691982 /// XM_604179	-5.8	4.6	7.2	6.8E-07
STAC3	SH3 and cysteine rich domain 3	NM_001192484	-6.0	5.1	7.7	1.1E-07
LOC100848767	uncharacterized LOC100848767	XR_138691 /// XR_139286	-6.0	5.2	7.8	8.9E-06
KIFC1	kinesin family member C1	NM_001101936	-6.0	6.0	8.5	2.1E-09
CA14	carbonic anhydrase XIV	NM_001192205	-6.0	7.0	9.5	8.6E-08
CRISPLD2	cysteine-rich secretory protein LCCL domain containing 2	NM_001100299	-6.0	8.1	10.7	1.8E-05
LOC100299061 /// LOC777601	uncharacterized LOC100299061 /// uncharacterized LOC777601	NM_001078148 /// XM_003584306 /// XM_003588131 /// XM_003588134	-6.2	6.1	8.8	1.8E-06
SVOPL	SVOP-like	NM_001192500	-6.2	4.5	7.2	5.9E-04
PLEK	pleckstrin	NM_001192496	-6.3	5.8	8.4	3.9E-06
LTF	lactotransferrin	NM_180998	-6.4	6.7	9.4	8.0E-07
CHRDL1	chordin-like 1	XM_592894	-6.5	7.2	9.9	5.5E-09
NALCN	sodium leak channel, non-selective	XM_002691980 /// XM_616223	-6.7	6.2	8.9	6.2E-05
LAMC2	laminin, gamma 2	XM_002694208 /// XM_588297	-6.8	5.9	8.7	2.9E-06
CCBL1 /// LOC100335550 /// LOC100852281 /// LOC781863	cysteine conjugate-beta lyase, cytoplasmic /// kynurenine--oxoglutarate transaminase 1-	NM_001077978 /// XM_001790512 /// XM_002706500 /// XM_002706501 /// XM_003585361 /// XM	-7.0	7.0	9.8	2.4E-07
LOC508666	C-C motif chemokine 23	NM_001244199	-7.1	5.8	8.6	1.4E-04
NUP210	nucleoporin 210kDa	NM_001103320 /// NM_001191461	-7.2	6.8	9.6	4.2E-10
GPT	glutamic-pyruvate transaminase (alanine aminotransferase)	NM_001083740	-7.2	6.8	9.6	3.0E-05
ACSS1	acyl-CoA synthetase short-chain family member 1	NM_174746	-7.3	5.7	8.6	3.9E-08

AMH	anti-Mullerian hormone	NM_173890	-7.7	7.1	10.0	9.9E-06
ABCC8	ATP-binding cassette, sub-family C (CFTR/MRP), member 8	NM_001205610	-7.8	4.5	7.5	3.7E-08
GYLTL1B	glycosyltransferase-like 1B	NM_001206192	-8.0	7.0	10.0	2.7E-05
LOC100337435	tumor necrosis factor alpha-induced protein 2-like	XR_084013	-8.5	7.8	10.9	1.3E-05
GANAB	glucosidase, alpha; neutral AB	NM_001205777	-8.6	7.1	10.2	3.5E-10
SLC22A3	solute carrier family 22 (extraneuronal monoamine transporter), member 3	XM_002690372 /// XM_002705253	-8.7	7.3	10.4	1.3E-06
ANGPT2	angiopoietin 2	NM_001098855	-8.8	6.8	10.0	1.2E-04
MYH11	myosin, heavy chain 11, smooth muscle	NM_001102127	-8.8	6.0	9.1	1.6E-05
HSPA1A	heat shock 70kDa protein 1A	NM_174550 /// NM_203322	-9.0	8.3	11.5	2.7E-11
FSHR	follicle stimulating hormone receptor	NM_174061	-9.1	5.6	8.8	2.8E-07
CYP19A1	cytochrome P450, family 19, subfamily A, polypeptide 1	NM_174305	-9.3	6.2	9.5	1.4E-03
SEP4	septin 4	NM_001034651	-10.0	6.2	9.5	4.5E-08
SLC27A3	solute carrier family 27 (fatty acid transporter), member 3	XM_001790632 /// XM_002686020	-10.0	4.3	7.6	1.7E-07
LOC100297594	uncharacterized LOC100297594	XM_003585353 /// XM_003587190	-11.3	5.0	8.5	3.3E-08
LOC404103	spleen trypsin inhibitor	NM_205786	-12.1	8.2	11.8	7.0E-10
GPC3	glypican 3	NM_001035463	-12.2	5.6	9.2	1.3E-09
PLA2G1B	phospholipase A2, group IB (pancreas)	NM_174646	-12.6	5.5	9.2	7.3E-08
GUCA1A	guanylate cyclase activator 1A (retina)	NM_174546	-12.6	5.3	8.9	1.3E-07
PTI	pancreatic trypsin inhibitor	NM_001001554	-12.8	6.2	9.9	2.8E-09
ETNK2	ethanolamine kinase 2	XM_002693881 /// XM_612564	-13.0	7.0	10.7	2.2E-08
HSD17B1 /// HSD17B1	hydroxysteroid (17-beta) dehydrogenase 1 /// hydroxysteroid (17-beta) dehydrogenase 1-l	NM_001102365 /// XM_001253407	-15.5	7.8	11.8	6.6E-05
MEST	mesoderm specific transcript homolog (mouse)	NM_001083368	-18.9	7.6	11.9	2.4E-09
CARTPT	CART prepropeptide	NM_001007820	-20.0	6.0	10.4	4.6E-06
IHH	Indian hedgehog	NM_001076870	-26.8	5.1	9.8	4.8E-09
C11H2orf40	chromosome 11 open reading frame, human C2orf40	NM_001038113	-45.5	5.0	10.5	6.0E-09

> 4-fold change, FDR P < 0.05
