# Supplementary material for: Transcriptomal profiling of bovine ovarian granulosa and theca interna cells in primary culture in comparison with their in vivo counterparts
Source: PLoS One. 2017 Mar 10;12(3):e0173391. doi: 10.1371/journal.pone.0173391 (PMC5345798; doi:10.1371/journal.pone.0173391)
Supplement: S2 Table — The complete gene list showing full names of the genes, fold changes, signal intensities and FDR values (> 4-fold change, FDR P < 0.05). (PDF) [file pone.0173391.s005.pdf]

**S2 Table. Genes which are differentially regulated *in vitro* compared with *in vivo* in cells from the theca layer**.

| Gene Symbol | Gene Title | RefSeq Transcript ID | Fold-Change | $Log_2$ Mean Intensity | | FDR *P*- value |
|---|---|---|---|---|---|---|
| | | | | *In vitro* | *In vivo* | |
| *BOLA /// BOLA* | MHC class I antigen clone 2 /// MHC class I heavy chain | NM_001038518 /// NM_001040532 /// XM_002697336 | 213.7 | 10.5 | 2.8 | 2.0E-11 |
| *CFB* | complement factor B | NM_001040526 | 123.4 | 11.2 | 4.2 | 4.8E-19 |
| *LOC790042 /// WARS* | Tryptophanyl-tRNA synthetase, cytoplasmic-like /// tryptophanyl-tRNA synthetase | NM_174218 /// XM_001256626 | 81.9 | 10.3 | 3.9 | 4.9E-18 |
| *NT5E* | 5'-nucleotidase, ecto (CD73) | NM_174129 | 59.4 | 9.9 | 4.0 | 5.4E-14 |
| *IDO1* | indoleamine 2,3-dioxygenase 1 | NM_001101866 | 58.3 | 9.4 | 3.5 | 2.0E-16 |
| *SPP1* | secreted phosphoprotein 1 | NM_174187 | 55.2 | 8.6 | 2.8 | 1.9E-14 |
| *LOC512486* | interferon-induced guanylate-binding protein 1 | NM_001244229 | 54.5 | 11.5 | 5.7 | 1.2E-14 |
| *LOC515676* | uncharacterized LOC515676 | XM_002695644 /// XM_593741 | 52.3 | 13.1 | 7.4 | 1.0E-11 |
| *CXCL6* | chemokine (C-X-C motif) ligand 6 (granulocyte chemotactic protein 2) | NM_174300 | 50.8 | 10.6 | 4.9 | 1.3E-15 |
| *CXCL2* | chemokine (C-X-C motif) ligand 2 | NM_174299 | 49.9 | 8.1 | 2.5 | 2.2E-18 |
| *GBP5* | guanylate binding protein 5 | NM_001075746 | 45.3 | 10.4 | 4.9 | 1.6E-16 |
| *ANKRD1* | ankyrin repeat domain 1 (cardiac muscle) | NM_001034378 | 43.2 | 9.0 | 3.5 | 5.3E-13 |
| *BoLA /// BOLA-A* | major histocompatibility complex, class I, A /// major histocompatibility complex, clas | NM_001040554 /// NM_001114855 | 43.0 | 10.4 | 5.0 | 4.0E-10 |
| *IL18* | interleukin 18 (interferon-gamma-inducing factor) | NM_174091 | 40.8 | 10.0 | 4.6 | 3.8E-14 |
| *NUPR1* | nuclear protein, transcriptional regulator, 1 | NM_001114515 | 40.8 | 9.9 | 4.6 | 9.3E-15 |
| *LOC504548* | ubiquitin D-like | XM_002697377 /// XM_580689 | 36.6 | 9.6 | 4.4 | 4.4E-13 |
| *C1S* | complement component 1, s subcomponent | NM_001076550 | 36.0 | 11.6 | 6.5 | 8.8E-14 |
| *SAA3* | serum amyloid A 3 | NM_181016 | 34.6 | 9.7 | 4.6 | 6.2E-13 |
| *CXCL10* | chemokine (C-X-C motif) ligand 10 | NM_001046551 | 32.6 | 12.0 | 7.0 | 5.9E-14 |
| *GPNMB* | glycoprotein (transmembrane) nmb | NM_001038065 | 30.1 | 9.3 | 4.4 | 4.7E-08 |
| *OLR1* | oxidized low density lipoprotein (lectin-like) receptor 1 | NM_174132 | 29.2 | 9.7 | 4.8 | 1.4E-10 |
| *CCL2* | chemokine (C-C motif) ligand 2 | NM_174006 | 27.9 | 10.2 | 5.4 | 4.0E-13 |

| | | | | | | |
|---|---|---|---|---|---|---|
| *BOLA-N /// JSP.1* | MHC class I antigen /// MHC Class I JSP.1 | NM_001040498 /// NM_001105651 | 27.8 | 12.1 | 7.4 | 5.6E-07 |
| *GBP4 /// LOC507055* | guanylate binding protein 4 /// guanylate binding protein 4-like | NM_001102261 /// XM_001788604 /// XM_002686269 | 27.2 | 9.8 | 5.0 | 8.5E-15 |
| *ERAP2* | endoplasmic reticulum aminopeptidase 2 | NM_001075628 | 27.2 | 8.5 | 3.7 | 1.3E-15 |
| *MX2* | myxovirus (influenza virus) resistance 2 (mouse) | NM_173941 | 27.1 | 8.1 | 3.4 | 7.4E-15 |
| *LOC504548 /// UBD* | ubiquitin D-like /// ubiquitin D | NM_001206473 /// XM_002697377 /// XM_580689 | 25.6 | 10.9 | 6.2 | 2.1E-11 |
| *CXCL11* | chemokine (C-X-C motif) ligand 11 | NM_001113173 | 25.1 | 9.9 | 5.2 | 2.0E-11 |
| *WARS* | tryptophanyl-tRNA synthetase | NM_174218 | 24.3 | 12.2 | 7.6 | 9.0E-16 |
| *RASSF4* | Ras association (RalGDS/AF-6) domain family member 4 | NM_001075454 | 24.1 | 8.3 | 3.7 | 1.4E-15 |
| *IL6* | interleukin 6 (interferon, beta 2) | NM_173923 | 23.6 | 7.4 | 2.9 | 5.7E-13 |
| *IL8* | interleukin 8 | NM_173925 | 23.1 | 8.0 | 3.5 | 6.9E-16 |
| *BoLA /// BOLA /// BOLA-A* | major histocompatibility complex, class I, A /// MHC class I heavy chain /// major hist | NM_001040554 /// NM_001114855 /// NM_001193296 | 23.1 | 8.3 | 3.8 | 1.9E-14 |
| *TNC* | tenascin C | NM_001078026 | 22.8 | 9.6 | 5.1 | 1.2E-11 |
| *TNFAIP6* | tumor necrosis factor, alpha-induced protein 6 | NM_001007813 | 21.6 | 7.3 | 2.9 | 5.5E-10 |
| *ATF3* | activating transcription factor 3 | NM_001046193 | 21.5 | 7.9 | 3.5 | 4.3E-13 |
| *CHI3L1 /// LOC788983* | chitinase 3-like 1 (cartilage glycoprotein-39) /// chitinase-3-like protein 1-like | NM_001080219 /// XM_001255877 | 21.4 | 7.6 | 3.2 | 9.9E-15 |
| *IRF1* | interferon regulatory factor 1 | NM_001191261 /// XM_003584726 | 21.0 | 10.8 | 6.4 | 6.1E-15 |
| *SERPINB2* | serpin peptidase inhibitor, clade B (ovalbumin), member 2 | NM_001192079 | 20.3 | 7.9 | 3.5 | 2.6E-12 |
| *MMP9* | matrix metallopeptidase 9 (gelatinase B, 92kDa gelatinase, 92kDa type IV collagenase) | NM_174744 | 20.2 | 8.0 | 3.6 | 9.9E-15 |
| *GRO1* | chemokine (C-X-C motif) ligand 1 (melanoma growth stimulating activity, alpha) | NM_175700 | 19.5 | 7.6 | 3.3 | 3.9E-15 |
| *C1R* | complement component 1, r subcomponent | NM_001034407 | 19.4 | 10.5 | 6.2 | 2.4E-14 |
| *CLDN1* | claudin 1 | NM_001001854 | 18.6 | 10.7 | 6.4 | 3.0E-09 |
| *SRRM2* | serine/arginine repetitive matrix 2 | XM_002697930 /// XM_587832 | 18.1 | 7.3 | 3.2 | 5.1E-14 |

| | | | | | | |
|---|---|---|---|---|---|---|
| *CD74* | CD74 molecule, major histocompatibility complex, class II invariant chain | NM_001034735 /// NM_174799 | 17.5 | 11.1 | 7.0 | 9.9E-13 |
| *CCL5* | chemokine (C-C motif) ligand 5 | NM_175827 | 17.4 | 9.1 | 5.0 | 1.2E-12 |
| *POSTN* | periostin, osteoblast specific factor | NM_001040479 | 17.1 | 8.9 | 4.8 | 1.1E-09 |
| *ARHGAP29* | Rho GTPase activating protein 29 | NM_001102485 | 16.8 | 8.4 | 4.3 | 7.2E-14 |
| *EFEMP1* | EGF containing fibulin-like extracellular matrix protein 1 | NM_001081717 | 16.8 | 9.8 | 5.7 | 3.9E-07 |
| *CCL3* | chemokine (C-C motif) ligand 3 | NM_174511 | 16.2 | 8.1 | 4.1 | 5.7E-14 |
| *LOC100850907 /// MAPK6* | mitogen-activated protein kinase 6-like /// mitogen-activated protein kinase 6 | XM_002690965 /// XM_002706554 /// XM_003585063 | 16.1 | 7.5 | 3.4 | 5.0E-15 |
| *GNLY /// LOC100300483 /// LOC100850470* | granulysin /// antimicrobial peptide NK-lysin-like /// antimicrobial peptide NK-lysin-l | NM_001075143 /// XM_002691364 /// XM_003582783 | 15.8 | 9.8 | 5.9 | 4.8E-11 |
| *ISG20* | interferon stimulated exonuclease gene 20kDa | XM_002696514 /// XM_583075 | 15.6 | 7.7 | 3.8 | 3.6E-14 |
| *BOLA-DQA5* | major histocompatibility complex, class II, DQ alpha 5 | NM_001012675 | 15.5 | 6.9 | 2.9 | 8.7E-11 |
| *AP2B1* | adaptor-related protein complex 2, beta 1 subunit | NM_001075125 /// XM_003587384 | 15.2 | 6.9 | 3.0 | 7.5E-15 |
| *BOLA-DQA2* | major histocompatibility complex, class II, DQ alpha 2 | NM_001012681 | 14.7 | 10.3 | 6.4 | 1.4E-04 |
| *EFR3A* | EFR3 homolog A (S. cerevisiae) | NM_001076047 | 14.5 | 9.1 | 5.3 | 3.4E-13 |
| *LOC510382* | guanylate-binding protein 4-like | XR_083420 /// XR_083633 | 14.5 | 8.5 | 4.6 | 9.8E-13 |
| *GNLY* | granulysin | NM_001075143 | 14.5 | 8.7 | 4.9 | 1.6E-10 |
| *MAP1LC3C* | microtubule-associated protein 1 light chain 3 gamma | NM_001101058 | 14.2 | 6.7 | 2.9 | 6.0E-14 |
| *IFIT2* | interferon-induced protein with tetratricopeptide repeats 2 | XM_001787823 /// XM_002698356 | 14.2 | 7.6 | 3.8 | 1.8E-13 |
| *LOC100297063* | uncharacterized LOC100297063 | XM_002697871 /// XM_002703055 | 14.1 | 6.9 | 3.1 | 7.8E-09 |
| *STEAP1* | six transmembrane epithelial antigen of the prostate 1 | NM_001205806 | 13.9 | 8.3 | 4.5 | 8.3E-10 |
| *LOC100847889 /// SERPING1* | uncharacterized LOC100847889 /// serpin peptidase inhibitor, clade G (C1 inhibitor), me | NM_174821 /// XM_003583189 /// XM_003587021 | 13.5 | 10.8 | 7.0 | 5.3E-13 |
| *BOLA /// BOLA /// BOLA /// BOLA-N* | MHC class I antigen /// MHC class I antigen clone 2 /// MHC class I heavy chain /// MHC | NM_001038518 /// NM_001040498 /// | 13.1 | 8.4 | 4.7 | 1.9E-14 |

| | | | | | | |
|---|---|---|---|---|---|---|
| */// BOLA-NC1 /// JSP.1 /// LOC100125916* | | NM_001040532 /// NM_001105487 /// NM_001105616 /// NM | | | | |
| *S100A8* | S100 calcium binding protein A8 | NM_001113725 | 12.8 | 7.6 | 3.9 | 1.7E-15 |
| *ITGAV* | integrin, alpha V (vitronectin receptor, alpha polypeptide, antigen CD51) | NM_174367 | 12.7 | 11.8 | 8.2 | 3.9E-13 |
| *S100A12* | S100 calcium binding protein A12 | NM_174651 | 12.3 | 7.7 | 4.0 | 7.8E-12 |
| *ISG15* | ISG15 ubiquitin-like modifier | NM_174366 | 12.1 | 10.7 | 7.1 | 2.4E-10 |
| *CFI* | complement factor I | NM_001038096 | 12.1 | 7.5 | 3.9 | 1.7E-12 |
| *CLCA3* | chloride channel regulator 3 | NM_181018 | 11.7 | 6.5 | 2.9 | 6.9E-11 |
| *LOC783920* | interferon-induced very large GTPase 1-like | XR_082927 | 11.3 | 7.1 | 3.6 | 2.1E-13 |
| *UBA7* | ubiquitin-like modifier activating enzyme 7 | NM_001012284 | 11.0 | 9.5 | 6.1 | 1.1E-14 |
| *VCAM1* | vascular cell adhesion molecule 1 | NM_174484 | 11.0 | 10.5 | 7.0 | 1.8E-10 |
| *BOLA-DMB* | major histocompatibility complex, class II, DM beta | NM_001040481 | 10.8 | 10.3 | 6.9 | 3.9E-13 |
| *C5H12orf35* | chromosome 5 open reading frame, human C12orf35 | NM_001101072 | 10.7 | 8.6 | 5.2 | 1.9E-11 |
| *GFPT2* | glutamine-fructose-6-phosphate transaminase 2 | NM_001076883 | 10.7 | 9.8 | 6.4 | 4.7E-11 |
| *LOC512440* | myeloid-associated differentiation marker-like | NM_001101956 | 10.5 | 8.7 | 5.3 | 1.6E-07 |
| *DRAM1* | DNA-damage regulated autophagy modulator 1 | NM_001031767 | 10.1 | 8.0 | 4.6 | 1.1E-13 |
| *BLA-DQB* | MHC class II antigen | NM_001034668 | 10.0 | 9.1 | 5.8 | 6.8E-05 |
| *WHSC1L1* | Wolf-Hirschhorn syndrome candidate 1-like 1 | NM_001075595 /// XM_003584171 /// XM_003584172 /// XM_003587971 /// XM_003587972 | 9.9 | 7.6 | 4.3 | 3.1E-11 |
| *SAMD9* | sterile alpha motif domain containing 9 | NM_001205781 | 9.9 | 7.5 | 4.2 | 1.4E-12 |
| *CHD2* | chromodomain helicase DNA binding protein 2 | NM_001102181 | 9.9 | 6.6 | 3.3 | 8.5E-15 |
| *TAP1* | transporter 1, ATP-binding cassette, sub-family B (MDR/TAP) | NM_001098058 | 9.8 | 11.1 | 7.8 | 9.9E-15 |
| *ADAM10* | ADAM metallopeptidase domain 10 | NM_174496 | 9.8 | 7.9 | 4.6 | 6.8E-14 |
| *ISM1* | isthmin 1 homolog (zebrafish) | NM_001163935 | 9.7 | 7.0 | 3.7 | 2.4E-12 |
| *TFPI2* | tissue factor pathway inhibitor 2 | NM_182788 | 9.6 | 10.5 | 7.2 | 6.2E-13 |

| | | | | | | |
|---|---|---|---|---|---|---|
| *USP7* | ubiquitin specific peptidase 7 (herpes virus-associated) | XM_002697958 /// XM_002703063 | 9.6 | 6.4 | 3.2 | 1.4E-13 |
| *IL13RA1* | interleukin 13 receptor, alpha 1 | NM_001206677 | 9.6 | 6.8 | 3.5 | 3.6E-14 |
| *LGALS3* | lectin, galactoside-binding, soluble, 3 | NM_001102341 | 9.4 | 9.5 | 6.3 | 3.2E-12 |
| *CIITA* | class II, major histocompatibility complex, transactivator | XM_002697962 /// XM_585540 | 9.4 | 8.1 | 4.9 | 3.2E-13 |
| *TPR* | translocated promoter region (to activated MET oncogene) | NM_001205623 | 9.3 | 6.1 | 2.9 | 7.4E-17 |
| *gzmA* | granzyme A | NM_001001142 | 9.3 | 5.9 | 2.7 | 1.1E-08 |
| *IQGAP1* | IQ motif containing GTPase activating protein 1 | NM_001191167 /// XM_002696571 /// XM_003583734 | 9.3 | 7.5 | 4.3 | 1.8E-10 |
| *SECTM1* | secreted and transmembrane 1 | NM_001102326 | 9.3 | 7.9 | 4.7 | 3.2E-12 |
| *SLC25A12* | solute carrier family 25 (mitochondrial carrier, Aralar), member 12 | NM_001101194 | 9.1 | 6.4 | 3.2 | 5.9E-14 |
| *CDKN1A* | cyclin-dependent kinase inhibitor 1A (p21, Cip1) | NM_001098958 | 9.1 | 9.1 | 5.9 | 2.8E-14 |
| *BST2* | bone marrow stromal cell antigen 2 | XM_002688576 /// XM_003582366 | 9.1 | 9.6 | 6.5 | 3.1E-11 |
| *ARL4C* | ADP-ribosylation factor-like 4C | NM_001102348 | 9.0 | 8.2 | 5.1 | 1.3E-11 |
| *PARM1* | prostate androgen-regulated mucin-like protein 1 | NM_001075771 | 8.9 | 6.7 | 3.6 | 6.2E-12 |
| *TIAM1* | T-cell lymphoma invasion and metastasis 1 | NM_001078009 /// XM_003581665 /// XM_003585616 | 8.9 | 7.8 | 4.7 | 1.7E-13 |
| *PSMB8* | proteasome (prosome, macropain) subunit, beta type, 8 (large multifunctional peptidase | NM_001040480 | 8.9 | 10.3 | 7.2 | 6.6E-15 |
| *LOC100848019* | uncharacterized LOC100848019 | XR_139386 | 8.9 | 10.1 | 7.0 | 3.2E-16 |
| *BOLA-DQB* | major histocompatibility complex, class II, DQ beta | NM_001012676 | 8.8 | 7.1 | 3.9 | 5.1E-11 |
| *PARP8* | poly (ADP-ribose) polymerase family, member 8 | NM_001192298 | 8.8 | 7.6 | 4.5 | 1.9E-14 |
| *CXCL16* | chemokine (C-X-C motif) ligand 16 | NM_001046095 | 8.8 | 10.4 | 7.2 | 1.9E-13 |
| *FLRT2* | leucine-rich repeat transmembrane protein FLRT2-like | XM_003582749 /// XM_003586603 | 8.7 | 10.6 | 7.5 | 1.8E-12 |
| *IFI44 /// LOC781857* | interferon-induced protein 44 /// interferon-induced protein 44-like | XM_002686295 /// XM_872122 /// XR_083444 | 8.6 | 8.6 | 5.5 | 1.3E-10 |
| *PKM* | pyruvate kinase, muscle | NM_001205727 | 8.6 | 9.7 | 6.6 | 4.8E-11 |

| | | | | | | |
|---|---|---|---|---|---|---|
| *PLAC8* | placenta-specific 8 | NM_001076987 | 8.5 | 9.5 | 6.4 | 1.7E-09 |
| *RNF168* | ring finger protein 168, E3 ubiquitin protein ligase | NM_001076289 | 8.4 | 7.2 | 4.2 | 1.3E-16 |
| *DYNC1LI2* | dynein, cytoplasmic 1, light intermediate chain 2 | NM_001206152 | 8.4 | 9.8 | 6.8 | 3.0E-17 |
| *ALCAM* | activated leukocyte cell adhesion molecule | NM_174238 | 8.3 | 6.4 | 3.3 | 1.0E-16 |
| *SDC4* | syndecan 4 | XM_002692330 /// XM_584869 | 8.3 | 8.5 | 5.4 | 2.2E-13 |
| *SULT1A1* | sulfotransferase family, cytosolic, 1A, phenol-preferring, member 1 | NM_177521 | 8.1 | 10.8 | 7.8 | 5.7E-12 |
| *SLC38A2* | solute carrier family 38, member 2 | NM_001082424 | 8.1 | 7.1 | 4.0 | 1.7E-12 |
| *FAM114A1* | family with sequence similarity 114, member A1 | XM_002688202 /// XM_588946 | 8.0 | 7.0 | 4.0 | 1.3E-12 |
| *HELZ* | helicase with zinc finger | NM_001076166 /// XM_001789612 /// XM_002696228 | 8.0 | 6.5 | 3.5 | 5.8E-10 |
| *TOB1* | transducer of ERBB2, 1 | NM_001077075 /// XM_001252475 | 7.9 | 10.7 | 7.7 | 1.1E-12 |
| *CCL4* | chemokine (C-C motif) ligand 4 | NM_001075147 | 7.8 | 8.3 | 5.3 | 2.8E-09 |
| *ABI3BP* | ABI family, member 3 (NESH) binding protein | NM_001080307 /// XM_003581686 /// XM_003585635 | 7.7 | 8.2 | 5.2 | 1.1E-07 |
| *PRF1* | perforin 1 (pore forming protein) | NM_001143735 | 7.7 | 6.9 | 3.9 | 2.0E-10 |
| *GOLGA4* | golgin A4 | NM_001192125 | 7.7 | 6.5 | 3.5 | 1.8E-11 |
| *MYO9A* | myosin IXA | XM_002690529 /// XM_593333 | 7.7 | 6.3 | 3.4 | 5.9E-14 |
| *LOC100851529 /// RTP4* | receptor-transporting protein 4-like /// receptor (chemosensory) transporter protein 4 | NM_001075961 /// XM_003585525 | 7.6 | 7.9 | 4.9 | 1.2E-11 |
| *IFNG* | interferon, gamma | NM_174086 | 7.6 | 6.6 | 3.7 | 2.1E-13 |
| *PDPN* | podoplanin | NM_001033120 | 7.6 | 9.7 | 6.8 | 2.4E-11 |
| *LOC100848019 /// SOD2* | uncharacterized LOC100848019 /// superoxide dismutase 2, mitochondrial | NM_201527 /// XR_139386 | 7.6 | 11.8 | 8.9 | 1.0E-15 |
| *FAS* | Fas (TNF receptor superfamily, member 6) | NM_174662 | 7.6 | 7.3 | 4.4 | 9.0E-16 |
| *BCL2A1* | BCL2-related protein A1 | NM_001037100 | 7.6 | 8.2 | 5.3 | 1.6E-16 |
| *LOC100852378* | uncharacterized LOC100852378 | XR_139167 | 7.5 | 6.5 | 3.6 | 2.1E-13 |

| | | | | | | |
|---|---|---|---|---|---|---|
| *TNFRSF12A* | tumor necrosis factor receptor superfamily, member 12A | NM_001206327 /// NM_001206328 | 7.5 | 7.8 | 4.9 | 1.0E-09 |
| *FN1* | fibronectin 1 | NM_001163778 | 7.5 | 6.1 | 3.2 | 9.8E-10 |
| *LOC508347* | interferon-induced protein 44-like | XM_003581993 /// XM_003585891 | 7.4 | 8.3 | 5.4 | 6.9E-11 |
| *GPX3* | glutathione peroxidase 3 (plasma) | NM_174077 | 7.3 | 6.5 | 3.6 | 1.1E-06 |
| *CA2* | carbonic anhydrase II | NM_178572 | 7.3 | 6.9 | 4.0 | 1.9E-08 |
| *PLD1* | phospholipase D1, phosphatidylcholine-specific | NM_001102001 | 7.3 | 5.9 | 3.0 | 1.1E-11 |
| *CLIC4* | chloride intracellular channel 4 | NM_001080218 | 7.2 | 11.1 | 8.2 | 2.1E-14 |
| *RSAD2* | radical S-adenosyl methionine domain containing 2 | NM_001045941 | 7.2 | 7.7 | 4.9 | 1.9E-08 |
| *IFIH1* | interferon induced with helicase C domain 1 | XM_002685338 /// XM_615590 | 7.2 | 7.2 | 4.4 | 2.8E-13 |
| *TIMP1* | TIMP metallopeptidase inhibitor 1 | NM_174471 | 7.2 | 12.5 | 9.7 | 1.7E-13 |
| *RBM5* | RNA binding motif protein 5 | NM_001046374 | 7.1 | 7.2 | 4.4 | 7.7E-07 |
| *PPA1* | pyrophosphatase (inorganic) 1 | NM_001075118 | 7.1 | 11.6 | 8.8 | 1.6E-09 |
| *ZNFX1* | zinc finger, NFX1-type containing 1 | NM_001205716 | 7.1 | 9.9 | 7.0 | 2.7E-13 |
| *IFI27* | putative ISG12(a) protein | NM_001038050 | 7.1 | 12.0 | 9.2 | 1.4E-09 |
| *IFI6* | interferon, alpha-inducible protein 6 | NM_001075588 | 7.1 | 11.3 | 8.5 | 1.1E-10 |
| *SP140* | SP140 nuclear body protein | NM_001075447 | 7.0 | 9.8 | 7.0 | 9.0E-16 |
| *GHR* | growth hormone receptor | NM_176608 | 7.0 | 6.0 | 3.2 | 2.0E-12 |
| *CLCA3 /// LOC784768* | chloride channel regulator 3 /// calcium-activated chloride channel | NM_001242583 /// NM_181018 | 7.0 | 5.4 | 2.6 | 2.5E-09 |
| *CNGA3* | cyclic nucleotide gated channel alpha 3 | NM_174279 | 7.0 | 6.6 | 3.8 | 3.1E-09 |
| *CBLB* | Cas-Br-M (murine) ecotropic retroviral transforming sequence b | NM_001205923 | 7.0 | 7.2 | 4.4 | 1.0E-09 |
| *CD40* | CD40 molecule, TNF receptor superfamily member 5 | NM_001105611 | 6.9 | 8.7 | 5.9 | 9.8E-12 |
| *PPFIBP1* | PTPRF interacting protein, binding protein 1 (liprin beta 1) | XM_002687731 /// XM_003582246 /// XM_003582247 /// XM_003586104 /// XM_003586105 /// XM | 6.8 | 6.6 | 3.9 | 6.9E-16 |
| *LOC614107* | hexokinase 2-like | XM_865470 | 6.8 | 7.0 | 4.3 | 1.9E-09 |

| | | | | | | |
|---|---|---|---|---|---|---|
| *CAPN6* | calpain 6 | NM_001192231 | 6.7 | 8.4 | 5.6 | 7.4E-10 |
| *TRAF3IP2* | TRAF3 interacting protein 2 | NM_001035483 | 6.7 | 8.2 | 5.5 | 6.4E-14 |
| *LOC100297676* | C-type lectin domain family 2 member G-like | XM_002687838 /// XM_002704428 | 6.7 | 10.5 | 7.7 | 3.3E-14 |
| *TOP1* | topoisomerase (DNA) I | NM_001206487 | 6.7 | 7.9 | 5.2 | 4.0E-10 |
| *ZAP70* | zeta-chain (TCR) associated protein kinase 70kDa | NM_001193017 | 6.7 | 6.5 | 3.7 | 5.4E-11 |
| *CSTB* | cystatin B (stefin B) | NM_001100362 | 6.7 | 7.3 | 4.6 | 1.4E-12 |
| *CASP4* | caspase 4, apoptosis-related cysteine peptidase | NM_176638 | 6.6 | 9.4 | 6.6 | 4.4E-15 |
| *LAP3* | leucine aminopeptidase 3 | NM_174098 | 6.6 | 11.1 | 8.4 | 3.6E-14 |
| *LOC100848920 /// RGS2* | regulator of G-protein signaling 2-like /// regulator of G-protein signaling 2, 24kDa | NM_001075596 /// XM_003583217 /// XM_003587052 | 6.5 | 9.0 | 6.3 | 1.2E-07 |
| *PDLIM7* | PDZ and LIM domain 7 (enigma) | NM_001017947 /// NM_001113251 | 6.5 | 6.7 | 4.0 | 1.8E-12 |
| *ERAP1* | endoplasmic reticulum aminopeptidase 1 | NM_001102003 | 6.4 | 8.7 | 6.1 | 3.9E-10 |
| *CXCR6* | chemokine (C-X-C motif) receptor 6 | NM_001014859 | 6.4 | 6.7 | 4.0 | 1.7E-09 |
| *CLDN11* | claudin 11 | NM_001035055 | 6.4 | 8.9 | 6.2 | 2.5E-05 |
| *IFI30* | interferon, gamma-inducible protein 30 | NM_001101251 | 6.3 | 10.5 | 7.8 | 4.0E-13 |
| *PARP9* | poly (ADP-ribose) polymerase family, member 9 | NM_001076828 | 6.3 | 9.1 | 6.5 | 1.9E-13 |
| *KRAS* | v-Ki-ras2 Kirsten rat sarcoma viral oncogene homolog | NM_001110001 | 6.2 | 9.5 | 6.8 | 1.7E-13 |
| *RASGEF1B* | RasGEF domain family, member 1B | NM_001083649 | 6.2 | 6.8 | 4.1 | 2.4E-11 |
| *XRN2* | 5'-3' exoribonuclease 2 | NM_001192472 | 6.2 | 8.7 | 6.1 | 9.1E-14 |
| *STAT3* | signal transducer and activator of transcription 3 (acute-phase response factor) | NM_001012671 | 6.2 | 7.6 | 5.0 | 6.3E-11 |
| *PTGS2* | prostaglandin-endoperoxide synthase 2 (prostaglandin G/H synthase and cyclooxygenase) | NM_174445 | 6.2 | 6.3 | 3.7 | 3.9E-07 |
| *PHLDB2* | pleckstrin homology-like domain, family B, member 2 | NM_001206308 | 6.2 | 7.4 | 4.8 | 2.2E-13 |
| *CD2* | CD2 molecule | NM_001011676 | 6.1 | 6.8 | 4.1 | 2.1E-10 |
| *SLC16A2* | solute carrier family 16, member 2 (monocarboxylic acid transporter 8) | NM_001206939 | 6.1 | 7.2 | 4.6 | 2.0E-09 |
| *FOXO1* | forkhead box O1 | XM_002691748 /// XM_583090 | 6.1 | 8.8 | 6.2 | 4.4E-10 |
| *TMEM150C* | transmembrane protein 150C | NM_001078001 | 6.1 | 8.8 | 6.2 | 1.8E-10 |

| | | | | | | |
|---|---|---|---|---|---|---|
| *BOLA /// BoLA /// BOLA-A* | MHC class I heavy chain /// major histocompatibility complex, class I, A /// major hist | NM_001040554 /// NM_001114855 /// XM_002697349 /// XM_584616 | 6.1 | 6.7 | 4.1 | 2.1E-03 |
| *SNX10* | sorting nexin 10 | NM_001075375 | 6.1 | 7.5 | 4.9 | 1.9E-14 |
| *STXBP1* | syntaxin binding protein 1 | NM_174619 | 6.1 | 7.5 | 4.9 | 3.6E-14 |
| *CD3E* | CD3e molecule, epsilon (CD3-TCR complex) | NM_174011 | 6.1 | 7.0 | 4.4 | 2.5E-12 |
| *NLRC5* | NLR family, CARD domain containing 5 | XM_001250846 /// XM_002694790 | 6.0 | 7.5 | 4.9 | 1.4E-12 |
| *RRAS* | related RAS viral (r-ras) oncogene homolog | NM_001038688 | 6.0 | 8.3 | 5.7 | 5.0E-15 |
| *HSP90AA1* | heat shock protein 90kDa alpha (cytosolic), class A member 1 | NM_001012670 | 6.0 | 10.7 | 8.1 | 7.5E-13 |
| *TAPBPL* | TAP binding protein-like | NM_001102121 | 5.9 | 8.8 | 6.3 | 3.2E-14 |
| *API5* | apoptosis inhibitor 5 | NM_001144083 | 5.9 | 8.7 | 6.1 | 2.8E-12 |
| *SMEK2* | SMEK homolog 2, suppressor of mek1 (Dictyostelium) | NM_001191150 | 5.9 | 9.8 | 7.2 | 6.1E-15 |
| *LOC100336681* | SECIS binding protein 2-like | XM_002690975 /// XM_003585091 | 5.9 | 9.0 | 6.4 | 2.4E-10 |
| *UBE2L6* | ubiquitin-conjugating enzyme E2L 6 | NM_001098917 | 5.9 | 8.7 | 6.2 | 1.3E-10 |
| *DEFB4A /// DEFB5 /// LOC100335951* | defensin, beta 4A /// defensin, beta 5 /// beta-defensin 4-like | NM_001130761 /// NM_174775 /// XM_002706761 | 5.8 | 6.1 | 3.5 | 1.0E-11 |
| *GDI1* | GDP dissociation inhibitor 1 | NM_174064 | 5.8 | 7.8 | 5.3 | 8.8E-14 |
| *CALD1* | caldesmon 1 | NM_174258 | 5.8 | 8.7 | 6.2 | 7.0E-13 |
| *XAF1* | XIAP associated factor 1 | NM_001035075 | 5.8 | 8.9 | 6.4 | 1.5E-09 |
| *MAN1A1* | mannosidase, alpha, class 1A, member 1 | NM_001205972 | 5.8 | 7.6 | 5.1 | 2.5E-09 |
| *EIF4G3* | eukaryotic translation initiation factor 4 gamma, 3 | NM_001078002 /// XM_003581895 /// XM_003581896 /// XM_003581897 /// XM_003581898 /// XM | 5.7 | 6.2 | 3.6 | 3.1E-11 |
| *PCOLCE2* | procollagen C-endopeptidase enhancer 2 | NM_001075629 | 5.7 | 8.3 | 5.7 | 5.5E-09 |
| *TPP1* | tripeptidyl peptidase I | NM_001075718 | 5.7 | 7.0 | 4.5 | 8.8E-14 |

| | | | | | | |
|---|---|---|---|---|---|---|
| *NUDT4* | nudix (nucleoside diphosphate linked moiety X)-type motif 4 | NM_001081618 | 5.7 | 7.8 | 5.3 | 4.6E-10 |
| *CCRL1* | chemokine (C-C motif) receptor-like 1 | NM_174265 | 5.7 | 5.5 | 3.0 | 1.2E-10 |
| *MR1* | major histocompatibility complex, class I-related | NM_001190298 | 5.7 | 6.2 | 3.7 | 7.3E-15 |
| *PSPH* | phosphoserine phosphatase | NM_001046355 | 5.6 | 8.6 | 6.1 | 2.9E-10 |
| *STAT1 /// STAT4* | signal transducer and activator of transcription 1, 91kDa /// signal transducer and act | NM_001077900 /// NM_001083692 /// XM_001787142 /// XM_002685461 | 5.6 | 11.8 | 9.3 | 1.6E-16 |
| *PIK3R1* | phosphoinositide-3-kinase, regulatory subunit 1 (alpha) | NM_174575 | 5.6 | 6.8 | 4.3 | 1.2E-10 |
| *LOC100851547 /// NMI* | N-myc-interactor-like /// N-myc (and STAT) interactor | NM_001035098 /// XM_003581830 | 5.6 | 10.1 | 7.6 | 1.1E-14 |
| *PSMB10* | proteasome (prosome, macropain) subunit, beta type, 10 | NM_001034040 | 5.6 | 10.4 | 7.9 | 3.6E-14 |
| *LOC100297914* | uncharacterized LOC100297914 | XM_002694408 /// XM_003583298 /// XM_003587136 | 5.6 | 6.8 | 4.3 | 3.1E-11 |
| *ATP6V1A* | ATPase, H+ transporting, lysosomal 70kDa, V1 subunit A | NM_174504 | 5.6 | 9.4 | 6.9 | 3.2E-12 |
| *LGALS3BP* | lectin, galactoside-binding, soluble, 3 binding protein | NM_001046316 | 5.5 | 10.3 | 7.9 | 6.1E-15 |
| *PDHA1* | pyruvate dehydrogenase (lipoamide) alpha 1 | NM_001101046 | 5.5 | 5.2 | 2.7 | 3.4E-13 |
| *TRIM21* | tripartite motif containing 21 | NM_182655 | 5.5 | 7.6 | 5.2 | 3.9E-13 |
| *PLBD1* | phospholipase B domain containing 1 | NM_001101044 /// NM_001166298 | 5.5 | 6.3 | 3.8 | 7.2E-07 |
| *CEACAM1* | carcinoembryonic antigen-related cell adhesion molecule 1 (biliary glycoprotein) | NM_205788 | 5.5 | 6.5 | 4.0 | 6.1E-15 |
| *MAR3* | membrane-associated ring finger (C3HC4) 3 | NM_001077941 | 5.5 | 5.8 | 3.4 | 7.9E-11 |
| *PARP14* | poly (ADP-ribose) polymerase family, member 14 | NM_001206538 | 5.5 | 9.6 | 7.1 | 7.6E-12 |
| *BOLA* | MHC class I heavy chain | NM_001038518 /// NM_001040532 | 5.5 | 13.1 | 10.7 | 4.7E-08 |
| *GLB1* | galactosidase, beta 1 | NM_001035043 | 5.5 | 6.3 | 3.8 | 1.9E-12 |
| *TNIP1* | TNFAIP3 interacting protein 1 | NM_001024554 | 5.5 | 8.1 | 5.6 | 6.1E-13 |
| *SLAMF8* | SLAM family member 8 | NM_001205794 | 5.4 | 7.6 | 5.1 | 1.4E-09 |
| *DPP4* | --- | --- | 5.4 | 6.1 | 3.7 | 3.6E-10 |
| *STAT2* | signal transducer and activator of transcription 2, 113kDa | NM_001205689 | 5.4 | 8.1 | 5.7 | 1.0E-15 |

| | | | | | | |
|---|---|---|---|---|---|---|
| *TET2* | tet methylcytosine dioxygenase 2 | XM_001790146 /// XM_002688092 /// XM_003582308 /// XM_003586185 | 5.4 | 7.0 | 4.6 | 1.3E-11 |
| *CHD2 /// LOC100847962* | chromodomain helicase DNA binding protein 2 /// chromodomain-helicase-DNA-binding prote | NM_001102181 /// XM_003583720 /// XM_003587543 | 5.4 | 7.1 | 4.7 | 8.6E-12 |
| *RAC2* | ras-related C3 botulinum toxin substrate 2 (rho family, small GTP binding protein Rac2) | NM_175792 | 5.4 | 8.1 | 5.7 | 3.1E-09 |
| *RNF128* | ring finger protein 128, E3 ubiquitin protein ligase | NM_001076071 | 5.3 | 8.7 | 6.3 | 3.3E-09 |
| *CCNT1* | cyclin T1 | NM_001001147 | 5.3 | 6.0 | 3.6 | 4.3E-13 |
| *GTF2H1* | general transcription factor IIH, polypeptide 1, 62kDa | NM_001046150 | 5.3 | 7.0 | 4.6 | 7.7E-15 |
| *BOLA-N /// JSP.1 /// LOC100125916* | MHC class I antigen /// MHC Class I JSP.1 /// uncharacterized protein 100125016 | NM_001040498 /// NM_001105487 /// NM_001105651 | 5.3 | 13.3 | 10.9 | 6.2E-09 |
| *ART3* | ADP-ribosyltransferase 3 | NM_001040496 | 5.3 | 6.6 | 4.2 | 8.4E-14 |
| *OAS1Y* | 2',5'-oligoadenylate synthetase 1, 40/46kDa | NM_001040606 | 5.3 | 9.9 | 7.5 | 4.0E-07 |
| *ZBTB33* | zinc finger and BTB domain containing 33 | NM_001098157 | 5.3 | 6.1 | 3.7 | 8.1E-13 |
| *GSTM1* | glutathione S-transferase M1 | NM_175825 | 5.3 | 9.4 | 7.0 | 8.1E-11 |
| *IL2RG* | interleukin 2 receptor, gamma | NM_174359 | 5.3 | 6.7 | 4.3 | 2.7E-11 |
| *LOC100850082* | uncharacterized LOC100850082 | XM_003582647 | 5.2 | 5.9 | 3.6 | 7.7E-12 |
| *LOC100126544* | uncharacterized LOC100126544 | NM_001110447 | 5.2 | 7.7 | 5.3 | 3.7E-14 |
| *BOLA-DMA* | major histocompatibility complex, class II, DM alpha | NM_001012674 | 5.2 | 8.8 | 6.4 | 1.2E-09 |
| *RNF19B* | ring finger protein 19B | NM_001205662 | 5.2 | 8.8 | 6.4 | 2.7E-10 |
| *EIF2AK2* | eukaryotic translation initiation factor 2-alpha kinase 2 | NM_178109 | 5.2 | 6.5 | 4.2 | 2.1E-11 |
| *SMOC2* | SPARC related modular calcium binding 2 | NM_001098134 | 5.2 | 9.8 | 7.4 | 3.9E-09 |
| *ARRDC2* | arrestin domain containing 2 | NM_001081592 | 5.2 | 6.4 | 4.1 | 3.2E-12 |
| *SERPINE1* | serpin peptidase inhibitor, clade E (nexin, plasminogen activator inhibitor type 1), me | NM_174137 | 5.2 | 7.4 | 5.1 | 8.2E-08 |
| *SF3B1* | splicing factor 3b, subunit 1, 155kDa | NM_001192994 | 5.2 | 7.4 | 5.0 | 1.1E-09 |
| *PYGL* | phosphorylase, glycogen, liver | NM_001075203 | 5.1 | 8.0 | 5.7 | 1.4E-12 |
| *HIATL1* | hippocampus abundant transcript-like 1 | NM_001083662 | 5.1 | 6.5 | 4.2 | 7.2E-14 |

| | | | | | | |
|---|---|---|---|---|---|---|
| *RDH11* | retinol dehydrogenase 11 (all-trans/9-cis/11-cis) | XM_002690979 /// XM_582373 | 5.1 | 6.5 | 4.1 | 1.6E-09 |
| *MX1* | myxovirus (influenza virus) resistance 1, interferon-inducible protein p78 (mouse) | NM_173940 | 5.1 | 10.2 | 7.9 | 8.6E-09 |
| *LOC530077* | gTPase, IMAP family member 5-like | XM_002687042 /// XM_002687043 /// XM_002704182 /// XM_864682 | 5.1 | 7.1 | 4.7 | 2.8E-08 |
| *CNP* | 2',3'-cyclic nucleotide 3' phosphodiesterase | NM_180993 | 5.1 | 7.1 | 4.7 | 6.1E-14 |
| *LPAR1* | lysophosphatidic acid receptor 1 | NM_174047 | 5.1 | 7.3 | 4.9 | 1.4E-12 |
| *BIRC3* | baculoviral IAP repeat containing 3 | NM_001035293 | 5.1 | 9.3 | 6.9 | 3.2E-13 |
| *PYCARD* | PYD and CARD domain containing | NM_174730 | 5.1 | 7.1 | 4.7 | 3.1E-10 |
| *GIMAP7* | GTPase, IMAP family member 7 | NM_001080257 | 5.1 | 7.2 | 4.9 | 1.1E-07 |
| *CXCL2 /// GRO1* | chemokine (C-X-C motif) ligand 2 /// chemokine (C-X-C motif) ligand 1 (melanoma growth | NM_001048165 /// NM_175700 | 5.1 | 4.8 | 2.5 | 2.3E-11 |
| *LOC507426* | poly [ADP-ribose] polymerase 3-like | XM_003583812 /// XM_003587648 | 5.0 | 7.2 | 4.8 | 2.3E-14 |
| *CD3G* | CD3g molecule, gamma (CD3-TCR complex) | NM_001040472 | 5.0 | 6.1 | 3.7 | 1.8E-09 |
| *GOLGB1* | golgin B1 | XM_001788217 /// XM_002684791 | 5.0 | 6.1 | 3.7 | 5.3E-14 |
| *TIPARP* | TCDD-inducible poly(ADP-ribose) polymerase | NM_001206048 | 5.0 | 7.0 | 4.6 | 7.4E-15 |
| *ARHGAP17* | Rho GTPase activating protein 17 | XM_002698028 /// XM_580330 | 5.0 | 5.9 | 3.6 | 6.6E-13 |
| *IL6ST* | interleukin 6 signal transducer (gp130, oncostatin M receptor) | XM_002696322 /// XM_600430 | 5.0 | 6.0 | 3.7 | 1.8E-11 |
| *NRG1* | neuregulin 1 | NM_174128 | 5.0 | 6.4 | 4.0 | 1.3E-10 |
| *DCLK1* | doublecortin-like kinase 1 | NM_001109962 | 5.0 | 5.5 | 3.2 | 1.8E-12 |
| *ITGA8* | integrin, alpha 8 | XM_002692081 /// XM_002701040 | 5.0 | 6.5 | 4.2 | 1.1E-08 |
| *HSPB6* | heat shock protein, alpha-crystallin-related, B6 | NM_001076027 | 5.0 | 6.8 | 4.5 | 3.6E-14 |
| *MSL3* | male-specific lethal 3 homolog (Drosophila) | NM_001192404 | 5.0 | 7.4 | 5.1 | 1.7E-09 |
| *EMP3* | epithelial membrane protein 3 | NM_001024562 | 4.9 | 8.5 | 6.2 | 1.2E-11 |
| *CD3D* | CD3d molecule, delta (CD3-TCR complex) | NM_001034033 | 4.9 | 6.4 | 4.1 | 8.4E-11 |
| *SLC31A2* | solute carrier family 31 (copper transporters), member 2 | NM_001034556 | 4.9 | 8.2 | 5.9 | 3.5E-13 |

| | | | | | | |
|---|---|---|---|---|---|---|
| *CREG1* | cellular repressor of E1A-stimulated genes 1 | NM_001075942 | 4.9 | 9.7 | 7.4 | 5.7E-11 |
| *IFI44* | interferon-induced protein 44 | XM_002686295 /// XM_872122 | 4.9 | 7.0 | 4.7 | 1.3E-08 |
| *SFRP2* | secreted frizzled-related protein 2 | NM_001034393 | 4.9 | 6.0 | 3.7 | 2.7E-06 |
| *KIT* | v-kit Hardy-Zuckerman 4 feline sarcoma viral oncogene homolog | NM_001166484 | 4.8 | 9.8 | 7.5 | 9.9E-10 |
| *CTSL1* | cathepsin L1 | NM_001083686 | 4.8 | 6.0 | 3.7 | 9.3E-11 |
| *SVEP1* | sushi, von Willebrand factor type A, EGF and pentraxin domain containing 1 | XM_002689921 /// XM_002705057 | 4.8 | 6.6 | 4.3 | 1.2E-10 |
| *MICU1* | mitochondrial calcium uptake 1 | NM_001075338 | 4.8 | 5.7 | 3.5 | 1.4E-10 |
| *CD48* | CD48 molecule | NM_001046002 | 4.8 | 7.3 | 5.0 | 3.0E-07 |
| *WDTC1* | WD and tetratricopeptide repeats 1 | NM_001205398 | 4.8 | 6.4 | 4.2 | 2.1E-13 |
| *ACSL5 /// LOC100851804* | acyl-CoA synthetase long-chain family member 5 /// long-chain-fatty-acid--CoA ligase 5- | NM_001075650 /// XM_003585076 | 4.8 | 9.7 | 7.4 | 1.0E-11 |
| *CD55* | CD55 molecule, decay accelerating factor for complement (Cromer blood group) | NM_001030303 | 4.8 | 8.3 | 6.0 | 8.2E-09 |
| *ANXA8L1* | annexin A8-like 1 | NM_174241 | 4.8 | 5.1 | 2.8 | 5.3E-08 |
| *LOC100337435* | tumor necrosis factor alpha-induced protein 2-like | XR_084013 | 4.8 | 10.1 | 7.8 | 6.9E-06 |
| *HIVEP2* | human immunodeficiency virus type I enhancer binding protein 2 | XM_002690289 /// XM_593747 | 4.8 | 7.9 | 5.6 | 9.5E-14 |
| *TNFSF13B* | tumor necrosis factor (ligand) superfamily, member 13b | NM_001114506 | 4.7 | 5.3 | 3.1 | 6.4E-13 |
| *CLIC2* | chloride intracellular channel 2 | NM_001081727 | 4.7 | 7.4 | 5.2 | 2.3E-14 |
| *PTI* | pancreatic trypsin inhibitor | NM_001001554 | 4.7 | 5.2 | 3.0 | 1.8E-12 |
| *IL2RA* | interleukin 2 receptor, alpha | NM_174358 | 4.7 | 5.8 | 3.6 | 5.9E-10 |
| *LOC100847279 /// STAM2* | signal transducing adapter molecule 2-like /// signal transducing adaptor molecule (SH3 | NM_001076106 /// XM_003585736 | 4.7 | 7.5 | 5.3 | 1.3E-14 |
| *LOC100335559 /// SQSTM1* | sequestosome-1-like /// sequestosome 1 | NM_176641 /// XM_002704190 | 4.7 | 11.0 | 8.8 | 1.3E-10 |
| *ZNF295* | zinc finger protein 295 | NM_001083659 | 4.7 | 6.8 | 4.6 | 1.8E-11 |
| *PML* | --- | --- | 4.7 | 7.6 | 5.4 | 3.9E-13 |
| *PIP4K2A* | phosphatidylinositol-5-phosphate 4-kinase, type II, alpha | NM_001192769 | 4.7 | 5.5 | 3.3 | 2.7E-13 |
| *MPP5* | membrane protein, palmitoylated 5 (MAGUK p55 subfamily member 5) | NM_001205951 | 4.7 | 8.3 | 6.1 | 5.5E-11 |

| | | | | | | |
|---|---|---|---|---|---|---|
| *GSK3B* | glycogen synthase kinase 3 beta | NM_001101310 | 4.7 | 6.6 | 4.4 | 9.0E-14 |
| *ZNF24* | zinc finger protein 24 | NM_001046445 /// NM_001205479 | 4.6 | 7.3 | 5.1 | 4.9E-07 |
| *AIDA* | axin interactor, dorsalization associated | NM_001205634 | 4.6 | 7.3 | 5.1 | 3.6E-12 |
| *CDO1* | cysteine dioxygenase, type I | NM_001034465 | 4.6 | 10.4 | 8.1 | 1.9E-10 |
| *ANKRD11* | ankyrin repeat domain 11 | XM_002694765 /// XM_612059 | 4.6 | 7.0 | 4.8 | 4.4E-11 |
| *CSDE1* | cold shock domain containing E1, RNA-binding | NM_001098025 | 4.6 | 6.1 | 3.9 | 2.9E-10 |
| *SBDS* | Shwachman-Bodian-Diamond syndrome | NM_001034439 | 4.6 | 8.9 | 6.7 | 1.8E-12 |
| *LOC100850526 /// SAMSN1* | uncharacterized LOC100850526 /// SAM domain, SH3 domain and nuclear localization signal | NM_001035404 /// XM_003585466 | 4.6 | 6.0 | 3.8 | 3.1E-09 |
| *TRD@* | T-cell receptor delta chain | --- | 4.6 | 6.1 | 3.9 | 3.5E-07 |
| *LOC512150* | myeloid-associated differentiation marker-like | NM_001104975 | 4.6 | 7.6 | 5.4 | 5.9E-11 |
| *BHLHE40* | basic helix-loop-helix family, member e40 | NM_001024929 | 4.6 | 6.7 | 4.5 | 6.7E-09 |
| *CHD4* | chromodomain helicase DNA binding protein 4 | NM_001206501 | 4.6 | 5.9 | 3.8 | 1.2E-11 |
| *CANX* | calnexin | NM_001105612 | 4.5 | 6.1 | 3.9 | 5.5E-11 |
| *CSN2* | casein beta | NM_181008 | 4.5 | 8.4 | 6.2 | 1.1E-11 |
| *CAST* | calpastatin | NM_001030318 /// NM_001030319 /// NM_001030320 /// NM_174003 | 4.5 | 9.2 | 7.0 | 1.7E-13 |
| *LOC100126815* | MHC class I-like family A1 | NM_001111069 | 4.5 | 6.9 | 4.7 | 1.1E-05 |
| *ANTXR2* | anthrax toxin receptor 2 | NM_001076826 | 4.5 | 6.8 | 4.6 | 2.1E-13 |
| *PAPD5* | PAP associated domain containing 5 | XM_001256515 /// XM_002694879 | 4.5 | 7.8 | 5.6 | 3.6E-14 |
| *TOX4* | TOX high mobility group box family member 4 | NM_001075707 | 4.5 | 6.5 | 4.3 | 5.4E-11 |
| *IL18BP* | interleukin 18 binding protein | XM_002693432 /// XM_869739 | 4.5 | 8.0 | 5.9 | 1.2E-11 |
| *MAP2* | microtubule-associated protein 2 | NM_001205807 | 4.5 | 5.6 | 3.5 | 1.1E-11 |
| *PLAUR* | plasminogen activator, urokinase receptor | NM_174423 | 4.5 | 6.6 | 4.4 | 3.2E-12 |
| *IL15* | interleukin 15 | NM_174090 | 4.5 | 5.7 | 3.5 | 5.5E-12 |
| *CD53* | CD53 molecule | NM_001034232 | 4.5 | 7.1 | 4.9 | 3.4E-07 |

| | | | | | | |
|---|---|---|---|---|---|---|
| *MARF1* | KIAA0430 | NM_001271994 /// XM_002698034 /// XM_583086 | 4.5 | 8.3 | 6.1 | 4.4E-13 |
| *HSD11B1* | hydroxysteroid (11-beta) dehydrogenase 1 | NM_001123032 | 4.5 | 6.0 | 3.8 | 5.7E-10 |
| *HTR2B* | 5-hydroxytryptamine (serotonin) receptor 2B | NM_001205448 | 4.5 | 5.4 | 3.3 | 7.5E-11 |
| *CLEC2D* | C-type lectin domain family 2, member D | XM_002687809 /// XM_002687810 /// XM_002704418 /// XM_869843 | 4.5 | 7.0 | 4.8 | 8.3E-09 |
| *TNFRSF6B* | tumor necrosis factor receptor superfamily, member 6b, decoy | NM_001101306 | 4.5 | 6.8 | 4.6 | 5.4E-11 |
| *CUL5* | cullin 5 | XM_002692966 /// XM_587459 | 4.5 | 5.2 | 3.0 | 2.2E-12 |
| *DTX3L* | deltex 3-like (Drosophila) | NM_001192396 | 4.5 | 9.9 | 7.7 | 2.1E-11 |
| *GLG1* | golgi glycoprotein 1 | NM_001244140 | 4.5 | 8.4 | 6.2 | 1.8E-12 |
| *GNS* | glucosamine (N-acetyl)-6-sulfatase | NM_001075562 | 4.5 | 10.1 | 7.9 | 1.7E-14 |
| *PTX3* | pentraxin 3, long | NM_001076259 | 4.4 | 8.9 | 6.8 | 2.7E-06 |
| *LOC100300510* | uncharacterized LOC100300510 | XM_002687093 /// XM_003582150 | 4.4 | 7.4 | 5.2 | 8.2E-08 |
| *UHRF1BP1L* | UHRF1 binding protein 1-like | NM_001192527 | 4.4 | 7.0 | 4.9 | 1.6E-11 |
| *IRF9* | interferon regulatory factor 9 | NM_001024506 | 4.4 | 8.4 | 6.3 | 1.8E-10 |
| *ODF2L* | outer dense fiber of sperm tails 2-like | NM_001075993 | 4.4 | 8.1 | 6.0 | 2.8E-11 |
| *NBEAL1* | neurobeachin-like 1 | NM_001103173 | 4.4 | 6.1 | 3.9 | 4.6E-15 |
| *TMEM106A* | transmembrane protein 106A | NM_001014870 | 4.4 | 8.4 | 6.3 | 7.0E-14 |
| *BOLA-DRB3* | major histocompatibility complex, class II, DRB3 | NM_001012680 | 4.4 | 10.6 | 8.5 | 2.3E-09 |
| *RNF213* | ring finger protein 213 | XM_002696144 /// XM_002702233 | 4.4 | 9.3 | 7.1 | 1.1E-09 |
| *SEMA5A* | sema domain, seven thrombospondin repeats (type 1 and type 1-like), transmembrane domai | XM_002696441 /// XM_583112 | 4.4 | 7.1 | 4.9 | 6.0E-11 |
| *IFNAR1* | interferon (alpha, beta and omega) receptor 1 | NM_174552 | 4.4 | 5.7 | 3.5 | 1.1E-13 |
| *CROT* | carnitine O-octanoyltransferase | NM_177494 | 4.4 | 8.2 | 6.1 | 1.0E-12 |
| *GZMB* | granzyme B (granzyme 2, cytotoxic T-lymphocyte-associated serine esterase 1) | XM_002696646 /// XM_585453 | 4.4 | 5.3 | 3.2 | 2.3E-09 |

| | | | | | | |
|---|---|---|---|---|---|---|
| *ARL6IP5* | ADP-ribosylation-like factor 6 interacting protein 5 | NM_001014891 | 4.4 | 10.3 | 8.2 | 3.4E-15 |
| *CLINT1* | clathrin interactor 1 | NM_001105417 | 4.4 | 6.3 | 4.2 | 1.5E-12 |
| *ARF3* | ADP-ribosylation factor 3 | NM_001015571 | 4.4 | 7.5 | 5.4 | 9.5E-13 |
| *CEBPD* | CCAAT/enhancer binding protein (C/EBP), delta | NM_174267 | 4.3 | 10.4 | 8.2 | 1.5E-07 |
| *ICAM1* | intercellular adhesion molecule 1 | NM_174348 | 4.3 | 9.3 | 7.2 | 3.3E-13 |
| *C2* | complement component 2 | NM_001034492 | 4.3 | 6.6 | 4.5 | 2.0E-12 |
| *LOC100852066 /// TCRA* | uncharacterized LOC100852066 /// T cell receptor, alpha | NM_001075519 /// NM_001098474 /// XR_139182 | 4.3 | 7.0 | 4.9 | 5.6E-09 |
| *PPM1K* | protein phosphatase, Mg2+/Mn2+ dependent, 1K | NM_001046474 | 4.3 | 7.7 | 5.6 | 7.3E-07 |
| *OSMR* | oncostatin M receptor | NM_001080272 /// XM_003583702 /// XM_003587530 | 4.3 | 8.2 | 6.1 | 4.8E-11 |
| *LOC534200* | aldehyde dehydrogenase family 1, subfamily A3-like | XM_001789815 | 4.3 | 7.1 | 5.0 | 4.7E-08 |
| *CASP3* | caspase 3, apoptosis-related cysteine peptidase | NM_001077840 | 4.3 | 5.3 | 3.2 | 5.6E-11 |
| *SHOC2* | soc-2 suppressor of clear homolog (C. elegans) | NM_001101943 | 4.3 | 4.8 | 2.8 | 5.1E-12 |
| *CD52* | CD52 molecule | NM_001198996 | 4.2 | 7.1 | 5.0 | 4.6E-08 |
| *GRAMD1C* | GRAM domain containing 1C | NM_001103229 | 4.2 | 7.8 | 5.7 | 3.8E-12 |
| *CDH1* | cadherin 1, type 1, E-cadherin (epithelial) | NM_001002763 | 4.2 | 5.3 | 3.3 | 1.6E-09 |
| *VLDLR* | very low density lipoprotein receptor | NM_174489 | 4.2 | 7.5 | 5.4 | 1.9E-09 |
| *SLC39A10* | solute carrier family 39 (zinc transporter), member 10 | NM_001205880 | 4.2 | 6.7 | 4.6 | 4.7E-12 |
| *COPA* | coatomer protein complex, subunit alpha | NM_001105645 | 4.2 | 4.9 | 2.8 | 3.4E-11 |
| *MTHFD2* | methylenetetrahydrofolate dehydrogenase (NADP+ dependent) 2, methenyltetrahydrofolate c | NM_001075755 | 4.2 | 7.9 | 5.8 | 3.9E-09 |
| *PLD3* | phospholipase D family, member 3 | NM_001078041 | 4.2 | 7.9 | 5.8 | 3.2E-11 |
| *HBP1* | HMG-box transcription factor 1 | NM_001046196 | 4.2 | 6.2 | 4.1 | 1.9E-10 |
| *NAV3* | neuron navigator 3 | NM_001192669 | 4.2 | 5.9 | 3.8 | 6.8E-10 |
| *NUDT12* | nudix (nucleoside diphosphate linked moiety X)-type motif 12 | NM_001046608 | 4.2 | 5.3 | 3.2 | 1.4E-14 |
| *LCP1* | lymphocyte cytosolic protein 1 (L-plastin) | NM_001034720 | 4.2 | 7.0 | 4.9 | 1.1E-08 |
| *LHCGR* | luteinizing hormone/choriogonadotropin receptor | NM_174381 | 4.2 | 7.0 | 5.0 | 1.3E-08 |
| *ADAMTSL4* | ADAMTS-like 4 | NM_001101061 | 4.2 | 6.9 | 4.9 | 3.3E-11 |
| *Mar-06* | membrane-associated ring finger (C3HC4) 6 | NM_001205812 | 4.2 | 6.1 | 4.0 | 4.2E-11 |

| | | | | | | |
|---|---|---|---|---|---|---|
| *CDADC1* | cytidine and dCMP deaminase domain containing 1 | NM_001206518 | 4.2 | 5.9 | 3.9 | 1.9E-14 |
| *EEF1A1* | eukaryotic translation elongation factor 1 alpha 1 | NM_174535 | 4.2 | 10.1 | 8.1 | 2.3E-10 |
| *APC* | adenomatous polyposis coli | NM_001075986 | 4.2 | 5.7 | 3.6 | 8.5E-09 |
| *NFKB2* | nuclear factor of kappa light polypeptide gene enhancer in B-cells 2 (p49/p100) | NM_001102101 | 4.1 | 5.7 | 3.7 | 1.8E-12 |
| *IER3* | immediate early response 3 | NM_001075202 | 4.1 | 8.7 | 6.7 | 1.8E-11 |
| *TMEM156* | transmembrane protein 156 | NM_001083469 | 4.1 | 5.6 | 3.5 | 1.9E-08 |
| *PLOD2* | procollagen-lysine, 2-oxoglutarate 5-dioxygenase 2 | NM_001101149 | 4.1 | 7.6 | 5.6 | 1.4E-08 |
| *NCOA7* | nuclear receptor coactivator 7 | NM_001102254 /// XM_003582598 /// XM_003586458 | 4.1 | 7.4 | 5.4 | 1.3E-13 |
| *PPP1CB* | protein phosphatase 1, catalytic subunit, beta isozyme | NM_001034653 | 4.1 | 7.3 | 5.3 | 5.4E-10 |
| *BMPR1A* | bone morphogenetic protein receptor, type IA | NM_001076800 | 4.1 | 6.1 | 4.0 | 2.7E-12 |
| *RYBP* | RING1 and YY1 binding protein | XM_002696972 /// XM_868187 | 4.1 | 8.1 | 6.1 | 7.7E-11 |
| *ATAD1* | ATPase family, AAA domain containing 1 | NM_001205581 | 4.1 | 6.5 | 4.4 | 1.4E-13 |
| *DPH3* | DPH3, KTI11 homolog (S. cerevisiae) | NM_001113299 | 4.1 | 6.0 | 4.0 | 2.0E-13 |
| *CPE* | carboxypeptidase E | NM_173903 | 4.1 | 8.2 | 6.2 | 1.7E-08 |
| *BOLA-NC1* | non-classical MHC class I antigen | NM_001105616 | 4.1 | 7.8 | 5.7 | 6.2E-11 |
| *PPP2R3C* | protein phosphatase 2, regulatory subunit B", gamma | NM_001015645 | 4.1 | 8.0 | 5.9 | 2.8E-12 |
| *CCR5* | chemokine (C-C motif) receptor 5 | NM_001011672 | 4.1 | 6.2 | 4.2 | 6.1E-08 |
| *TNPO1* | transportin 1 | NM_001076540 | 4.1 | 6.0 | 3.9 | 9.7E-11 |
| *AGFG1* | ArfGAP with FG repeats 1 | NM_001038171 | 4.1 | 8.6 | 6.6 | 3.1E-10 |
| *LATS1* | LATS, large tumor suppressor, homolog 1 (Drosophila) | NM_001192866 | 4.1 | 6.1 | 4.1 | 5.8E-10 |
| *ASPH* | aspartate beta-hydroxylase | NM_174757 | 4.1 | 5.6 | 3.6 | 4.2E-12 |
| *ACSS2* | acyl-CoA synthetase short-chain family member 2 | NM_001105339 | 4.0 | 8.3 | 6.3 | 2.4E-05 |
| *LOC100849396 /// PPP1R11* | protein phosphatase 1 regulatory subunit 11-like /// protein phosphatase 1, regulatory | NM_001100295 /// XM_003583880 /// XM_003583881 | 4.0 | 7.0 | 5.0 | 5.4E-14 |
| *LOC100848765* | uncharacterized LOC100848765 | XR_139527 | 4.0 | 9.0 | 7.0 | 1.4E-11 |
| *NUB1* | negative regulator of ubiquitin-like proteins 1 | NM_001080906 | 4.0 | 7.6 | 5.6 | 9.0E-16 |
| *CHD1* | chromodomain helicase DNA binding protein 1 | NM_001192048 | 4.0 | 7.5 | 5.5 | 2.6E-05 |
| *HMGB2* | high mobility group box 2 | NM_001037616 | -4.0 | 8.5 | 10.5 | 5.7E-08 |

| | | | | | | |
|---|---|---|---|---|---|---|
| *LIG1* | ligase I, DNA, ATP-dependent | NM_001102548 | -4.0 | 4.3 | 6.4 | 3.9E-08 |
| *PIR* | pirin (iron-binding nuclear protein) | NM_001102358 /// XM_003584351 | -4.0 | 3.7 | 5.7 | 6.0E-11 |
| *MRC2* | mannose receptor, C type 2 | NM_001192670 | -4.0 | 7.3 | 9.3 | 6.2E-09 |
| *ROR2* | receptor tyrosine kinase-like orphan receptor 2 | NM_001105464 | -4.0 | 5.2 | 7.2 | 3.8E-08 |
| *MAGOHB* | mago-nashi homolog B (Drosophila) | NM_001076252 | -4.0 | 5.7 | 7.7 | 3.9E-10 |
| *TMEM204* | transmembrane protein 204 | NM_001076377 | -4.0 | 6.4 | 8.4 | 1.1E-12 |
| *RASL11B* | RAS-like, family 11, member B | NM_001015635 | -4.1 | 3.8 | 5.9 | 7.3E-09 |
| *GYPC* | glycophorin C (Gerbich blood group) | NM_001002886 | -4.1 | 7.8 | 9.8 | 1.2E-10 |
| *CENP-A /// CENPA /// LOC618307 /// LOC782601 /// LOC782634* | centromere protein-A /// centromere protein A /// Histone H3-like centromeric protein A | NM_001205380 /// XM_001250060 /// XM_001250108 /// XM_001250161 /// XM_001250208 /// XM | -4.1 | 4.6 | 6.7 | 1.9E-07 |
| *HDAC7* | histone deacetylase 7 | NM_001193141 | -4.1 | 6.2 | 8.2 | 3.8E-11 |
| *RHNO1* | chromosome 5 open reading frame, human C12orf32 | NM_001075631 | -4.1 | 6.5 | 8.5 | 2.0E-08 |
| *DOCK6* | dedicator of cytokinesis 6 | NM_001192166 | -4.1 | 6.5 | 8.5 | 1.2E-09 |
| *KLF11* | Kruppel-like factor 11 | NM_001190301 | -4.1 | 4.6 | 6.6 | 2.3E-12 |
| *FOXF1* | forkhead box F1 | XM_002694754 /// XM_003583371 | -4.1 | 4.1 | 6.2 | 4.5E-09 |
| *FLT1* | fms-related tyrosine kinase 1 (vascular endothelial growth factor/vascular permeability | NM_001191132 | -4.2 | 4.2 | 6.3 | 7.0E-13 |
| *MECOM* | MDS1 and EVI1 complex locus | XM_002684943 /// XM_003581739 /// XM_003581740 /// XM_003581741 /// XM_003581742 /// XM | -4.2 | 3.7 | 5.8 | 4.4E-10 |
| *MFSD7* | major facilitator superfamily domain containing 7 | XM_003582356 /// XM_003586230 | -4.2 | 5.2 | 7.3 | 1.2E-12 |
| *BUB1B /// LOC100851213* | budding uninhibited by benzimidazoles 1 homolog beta (yeast) /// mitotic checkpoint ser | NM_001145173 /// XM_003582704 | -4.2 | 6.2 | 8.3 | 3.1E-06 |
| *EDNRA* | endothelin receptor type A | NM_174308 | -4.2 | 5.4 | 7.5 | 6.2E-11 |

| | | | | | | |
|---|---|---|---|---|---|---|
| *NR5A2* | nuclear receptor subfamily 5, group A, member 2 | NM_001206816 | -4.2 | 3.3 | 5.4 | 8.5E-05 |
| *UHRF1* | ubiquitin-like with PHD and ring finger domains 1 | NM_001103098 | -4.2 | 5.4 | 7.5 | 2.2E-04 |
| *NID1* | nidogen 1 | NM_001101155 | -4.2 | 7.7 | 9.8 | 3.4E-11 |
| *GCLC* | glutamate-cysteine ligase, catalytic subunit | NM_001083674 | -4.2 | 3.9 | 6.0 | 6.3E-09 |
| *KHDRBS1* | KH domain containing, RNA binding, signal transduction associated 1 | NM_001046442 | -4.2 | 3.4 | 5.5 | 1.6E-12 |
| *LOC100849055* | uncharacterized LOC100849055 | XR_139071 /// XR_139716 | -4.2 | 3.1 | 5.2 | 1.3E-07 |
| *DYSF* | dysferlin, limb girdle muscular dystrophy 2B (autosomal recessive) | NM_001102490 | -4.2 | 5.4 | 7.5 | 3.6E-09 |
| *TSPAN4* | tetraspanin 4 | NM_001101852 | -4.2 | 5.9 | 7.9 | 1.2E-12 |
| *CDC20* | cell division cycle 20 homolog (S. cerevisiae) | NM_001082436 | -4.3 | 6.2 | 8.3 | 8.3E-06 |
| *PTPRB* | protein tyrosine phosphatase, receptor type, B | NM_001205297 | -4.3 | 3.9 | 6.0 | 1.8E-10 |
| *CCL16* | chemokine (C-C motif) ligand 16 | XM_002695627 /// XM_868834 | -4.3 | 2.8 | 4.9 | 2.4E-14 |
| *CCNB2* | cyclin B2 | NM_174264 | -4.3 | 6.0 | 8.1 | 6.8E-06 |
| *ITGA7* | integrin, alpha 7 | NM_001191305 | -4.3 | 4.0 | 6.2 | 1.2E-13 |
| *SCG5* | secretogranin V (7B2 protein) | NM_001045998 | -4.3 | 5.5 | 7.6 | 4.8E-07 |
| *SPAG5* | sperm associated antigen 5 | NM_001205549 | -4.3 | 5.6 | 7.7 | 2.1E-06 |
| *ZNF521* | zinc finger protein 521 | NM_001105419 | -4.3 | 4.6 | 6.8 | 2.3E-10 |
| *CYGB* | cytoglobin | NM_001206720 | -4.4 | 4.8 | 6.9 | 6.8E-13 |
| *ALAD* | aminolevulinate dehydratase | NM_001014895 | -4.4 | 7.2 | 9.3 | 6.2E-13 |
| *REM1* | RAS (RAD and GEM)-like GTP-binding 1 | NM_001046001 | -4.4 | 3.6 | 5.7 | 4.0E-11 |
| *CCND2* | cyclin D2 | NM_001076372 | -4.4 | 6.8 | 9.0 | 4.1E-10 |
| *GLTSCR2* | glioma tumor suppressor candidate region gene 2 | NM_001038507 | -4.4 | 5.8 | 7.9 | 3.1E-10 |
| *PECAM1* | platelet/endothelial cell adhesion molecule | NM_174571 | -4.4 | 5.7 | 7.9 | 5.0E-09 |
| *HSPA1A* | heat shock 70kDa protein 1A | NM_174550 /// NM_203322 | -4.4 | 7.5 | 9.7 | 9.7E-11 |
| *GRHL1* | grainyhead-like 1 (Drosophila) | NM_001076450 | -4.5 | 4.7 | 6.8 | 1.7E-11 |
| *ETS2* | v-ets erythroblastosis virus E26 oncogene homolog 2 (avian) | NM_001080214 | -4.5 | 4.4 | 6.6 | 1.3E-11 |
| *ZNF532* | zinc finger protein 532 | XM_002697837 /// XM_613386 | -4.5 | 5.3 | 7.5 | 3.1E-13 |

| | | | | | | |
|---|---|---|---|---|---|---|
| *LOC781493* | collagen alpha-1(XIV) chain-like | XM_003583071 /// XM_003586917 | -4.5 | 7.0 | 9.2 | 1.1E-09 |
| *LOC782642 /// MAL* | mal, T-cell differentiation protein-like /// mal, T-cell differentiation protein | NM_001075428 /// XM_001251278 | -4.6 | 3.0 | 5.2 | 8.1E-07 |
| *DUT* | deoxyuridine triphosphatase | NM_001077873 | -4.6 | 8.4 | 10.6 | 1.5E-08 |
| *HJURP* | Holliday junction recognition protein | XM_002686557 /// XM_869720 | -4.6 | 6.1 | 8.3 | 3.6E-06 |
| *ITGA10* | integrin, alpha 10 | NM_001205590 | -4.6 | 4.5 | 6.7 | 1.3E-10 |
| *TGFBI* | transforming growth factor, beta-induced, 68kDa | NM_001205402 | -4.6 | 4.7 | 6.9 | 1.1E-11 |
| *PDK4* | pyruvate dehydrogenase kinase, isozyme 4 | NM_001101883 | -4.6 | 6.1 | 8.3 | 7.4E-10 |
| *ATRX* | alpha thalassemia/mental retardation syndrome X-linked | XM_002699982 /// XM_002699983 /// XM_002707277 /// XM_592333 | -4.6 | 5.9 | 8.1 | 1.3E-13 |
| *NNAT* | neuronatin | NM_001201324 /// NM_178323 | -4.6 | 4.4 | 6.6 | 1.4E-08 |
| *NUSAP1* | nucleolar and spindle associated protein 1 | NM_001046571 | -4.6 | 6.5 | 8.7 | 3.8E-06 |
| *LOC100848911 /// PTTG1* | securin-like /// pituitary tumor-transforming 1 | NM_001034310 /// XM_003582470 /// XM_003582471 /// XM_003582472 /// XM_003586337 /// XM | -4.6 | 7.3 | 9.5 | 2.6E-05 |
| *NPNT* | nephronectin | XM_002688090 /// XM_003586181 /// XM_003586182 /// XM_003586183 /// XM_003586184 /// XM | -4.6 | 5.8 | 8.0 | 6.3E-11 |
| *ID1* | inhibitor of DNA binding 1, dominant negative helix-loop-helix protein | NM_001097568 | -4.6 | 7.5 | 9.7 | 1.6E-11 |
| *LBH* | limb bud and heart development homolog (mouse) | NM_001099152 | -4.7 | 4.9 | 7.1 | 4.4E-10 |
| *TLE2* | transducin-like enhancer of split 2 (E(sp1) homolog, Drosophila) | XM_002688945 /// XM_870478 | -4.7 | 4.4 | 6.7 | 9.0E-14 |

| | | | | | | |
|---|---|---|---|---|---|---|
| *NDRG2* | NDRG family member 2 | NM_001035304 | -4.7 | 4.9 | 7.2 | 1.5E-09 |
| *DNM1* | dynamin 1 | NM_001076820 | -4.7 | 4.7 | 6.9 | 5.6E-11 |
| *RPRD1A* | regulation of nuclear pre-mRNA domain containing 1A | NM_001075156 | -4.7 | 4.5 | 6.7 | 8.1E-14 |
| *ASPM* | asp (abnormal spindle) homolog, microcephaly associated (Drosophila) | NM_001206506 | -4.8 | 5.0 | 7.2 | 5.7E-06 |
| *ELTD1* | EGF, latrophilin and seven transmembrane domain containing 1 | NM_001076908 | -4.8 | 6.0 | 8.2 | 3.6E-10 |
| *STAG3* | stromal antigen 3 | NM_001192411 | -4.8 | 3.5 | 5.8 | 2.7E-10 |
| *AGRN* | agrin | XM_002694193 /// XM_604151 | -4.8 | 7.0 | 9.2 | 1.1E-13 |
| *IQSEC1* | IQ motif and Sec7 domain 1 | NM_001206814 | -4.8 | 5.2 | 7.5 | 3.2E-11 |
| *MYC* | v-myc myelocytomatosis viral oncogene homolog (avian) | NM_001046074 | -4.9 | 7.6 | 9.8 | 9.4E-13 |
| *PEAR1* | platelet endothelial aggregation receptor 1 | NM_001101300 | -4.9 | 5.3 | 7.5 | 8.2E-12 |
| *CDCA7* | cell division cycle associated 7 | NM_001037488 | -4.9 | 5.2 | 7.5 | 2.8E-05 |
| *RAMP3* | receptor (G protein-coupled) activity modifying protein 3 | NM_001083505 | -4.9 | 4.6 | 6.9 | 1.7E-10 |
| *CHST7* | carbohydrate (N-acetylglucosamine 6-O) sulfotransferase 7 | NM_001193203 | -4.9 | 4.7 | 7.0 | 1.4E-12 |
| *TOP2A* | topoisomerase (DNA) II alpha 170kDa | XM_001254457 /// XM_002696009 | -4.9 | 7.1 | 9.4 | 2.8E-06 |
| *ENC1* | ectodermal-neural cortex 1 (with BTB-like domain) | NM_001078067 | -4.9 | 7.8 | 10.1 | 8.3E-13 |
| *EPHX1* | epoxide hydrolase 1, microsomal (xenobiotic) | NM_001034629 | -4.9 | 4.1 | 6.4 | 3.3E-10 |
| *MFAP2* | microfibrillar-associated protein 2 | NM_174388 | -4.9 | 5.4 | 7.7 | 4.2E-11 |
| *TNFRSF21* | tumor necrosis factor receptor superfamily, member 21 | NM_001076911 /// XM_003583868 | -4.9 | 4.1 | 6.4 | 9.0E-11 |
| *JAM2* | junctional adhesion molecule 2 | NM_001083736 | -4.9 | 5.8 | 8.1 | 1.9E-12 |
| *MEST* | mesoderm specific transcript homolog (mouse) | NM_001083368 | -5.0 | 6.4 | 8.7 | 6.7E-09 |
| *ERG* | v-ets erythroblastosis virus E26 oncogene homolog (avian) | NM_001102183 | -5.0 | 4.7 | 7.1 | 2.6E-12 |
| *CSPG4* | chondroitin sulfate proteoglycan 4 | NM_001192782 | -5.0 | 4.2 | 6.5 | 5.4E-16 |
| *LOC100850718* | uncharacterized LOC100850718 | XR_139132 | -5.0 | 4.3 | 6.6 | 5.2E-09 |
| *ITGBL1* | integrin, beta-like 1 (with EGF-like repeat domains) | NM_001206834 | -5.1 | 5.5 | 7.8 | 1.9E-09 |
| *RAB38* | RAB38, member RAS oncogene family | XM_001252194 /// XM_002699047 | -5.1 | 4.2 | 6.5 | 5.5E-09 |
| *LMNB1* | lamin B1 | NM_001103295 | -5.1 | 4.7 | 7.1 | 7.0E-07 |
| *OIT3* | oncoprotein induced transcript 3 | NM_001046064 | -5.1 | 3.7 | 6.1 | 4.9E-08 |

| | | | | | | |
|---|---|---|---|---|---|---|
| *SELP* | selectin P (granule membrane protein 140kDa, antigen CD62) | NM_174183 | -5.1 | 3.3 | 5.6 | 1.4E-11 |
| *H4 /// LOC100848381 /// LOC100849624 /// LOC100849654 /// LOC516742 /// LOC517138 /// LOC517481 /// LOC518961 /// LOC526789 /// LOC527388 /// LOC527645 /// LOC528329 /// LOC529647 /// LOC530773 /// LOC616167 /// LOC617875 /// LOC617905 /// LOC781223 /// LOC787485 /// LOC789113* | histone H4 /// histone H4-like /// histone H4-like /// histone H4-like /// histone clus | NM_001099724 /// NM_001145871 /// NM_173880 /// XM_001249844 /// XM_001253548 /// XM_00 | -5.1 | 5.4 | 7.7 | 2.4E-09 |
| *UBE2G2* | ubiquitin-conjugating enzyme E2G 2 | NM_001076328 | -5.1 | 4.6 | 7.0 | 1.6E-16 |
| *GJC1* | gap junction protein, gamma 1, 45kDa | NM_001046076 | -5.1 | 3.9 | 6.2 | 1.1E-10 |
| *NEDD9* | neural precursor cell expressed, developmentally down-regulated 9 | NM_001101847 | -5.1 | 5.7 | 8.1 | 2.2E-10 |
| *LEF1* | lymphoid enhancer-binding factor 1 | NM_001192856 | -5.1 | 4.2 | 6.5 | 1.8E-11 |
| *TM4SF1* | transmembrane 4 L six family member 1 | NM_001075980 | -5.1 | 6.1 | 8.5 | 3.3E-11 |
| *SERTAD4* | SERTA domain containing 4 | NM_001193037 | -5.1 | 3.8 | 6.2 | 6.7E-10 |
| *CIT* | citron (rho-interacting, serine/threonine kinase 21) | XM_001254412 /// XM_002694582 | -5.1 | 5.3 | 7.6 | 1.1E-07 |
| *PCSK6* | proprotein convertase subtilisin/kexin type 6 | XM_002696654 /// XM_603014 | -5.2 | 4.1 | 6.5 | 6.4E-13 |
| *FAM122B* | family with sequence similarity 122B | NM_001083794 | -5.2 | 6.9 | 9.3 | 3.2E-12 |
| *CA4* | carbonic anhydrase IV | NM_173897 | -5.2 | 4.4 | 6.8 | 2.5E-08 |

| | | | | | | |
|---|---|---|---|---|---|---|
| *ROBO4* | roundabout homolog 4, magic roundabout (Drosophila) | NM_001099191 | -5.2 | 5.2 | 7.6 | 3.6E-12 |
| *LOC788205* | uncharacterized LOC788205 | NM_001105644 | -5.2 | 3.8 | 6.2 | 1.5E-11 |
| *PLCB4* | phospholipase C, beta 4 | NM_001166510 /// NM_174424 | -5.3 | 6.5 | 8.9 | 1.1E-11 |
| *LMNB2* | lamin B2 | NM_001276353 /// XM_002688951 /// XM_594479 | -5.3 | 5.9 | 8.3 | 9.6E-07 |
| *SLC9A3R2* | solute carrier family 9 (sodium/hydrogen exchanger), member 3 regulator 2 | NM_001077065 | -5.3 | 5.3 | 7.7 | 7.5E-15 |
| *GATA2* | GATA binding protein 2 | NM_001192114 | -5.3 | 3.7 | 6.1 | 3.2E-14 |
| *UPK1B* | uroplakin 1B | NM_174482 | -5.3 | 3.7 | 6.1 | 1.5E-05 |
| *SH3BGRL2* | SH3 domain binding glutamic acid-rich protein like 2 | NM_001083791 | -5.3 | 4.3 | 6.7 | 1.6E-10 |
| *ID3* | inhibitor of DNA binding 3, dominant negative helix-loop-helix protein | NM_001014950 | -5.4 | 6.7 | 9.2 | 2.7E-10 |
| *CKB /// LOC100851864* | creatine kinase, brain /// creatine kinase B-type-like | NM_001015613 /// XM_003583773 | -5.4 | 4.9 | 7.3 | 3.2E-10 |
| *TIE1* | tyrosine kinase with immunoglobulin-like and EGF-like domains 1 | NM_173965 | -5.5 | 3.5 | 6.0 | 3.0E-14 |
| *LAMC3* | laminin, gamma 3 | XM_002691608 /// XM_597117 | -5.5 | 5.0 | 7.5 | 1.0E-10 |
| *TEK* | TEK tyrosine kinase, endothelial | NM_173964 | -5.5 | 5.3 | 7.8 | 7.0E-13 |
| *ARAP3* | ArfGAP with RhoGAP domain, ankyrin repeat and PH domain 3 | XM_003582447 /// XM_003586307 | -5.5 | 4.0 | 6.5 | 1.3E-14 |
| *PDLIM3* | PDZ and LIM domain 3 | NM_001034646 | -5.6 | 5.6 | 8.1 | 2.6E-08 |
| *CP* | ceruloplasmin (ferroxidase) | NM_001256556 /// XM_002685026 /// XM_592003 | -5.6 | 2.8 | 5.3 | 2.1E-11 |
| *PPP2R2B* | protein phosphatase 2, regulatory subunit B, beta | NM_001014879 /// NM_001272085 /// NR_073586 | -5.6 | 3.5 | 6.0 | 1.1E-08 |
| *RN18S1* | 18S ribosomal RNA | NR_036642 | -5.6 | 6.0 | 8.5 | 1.2E-03 |
| *MMRN1* | multimerin 1 | NM_001102500 | -5.7 | 2.9 | 5.4 | 7.5E-08 |
| *KDR* | kinase insert domain receptor (a type III receptor tyrosine kinase) | NM_001110000 | -5.7 | 5.4 | 7.9 | 1.6E-13 |

| | | | | | | |
|---|---|---|---|---|---|---|
| *ITGA6* | integrin, alpha 6 | NM_001109981 | -5.7 | 6.9 | 9.4 | 1.1E-11 |
| *SMIM10* | small integral membrane protein 10 | NM_001163442 | -5.9 | 3.7 | 6.3 | 4.6E-11 |
| *UBE2C* | ubiquitin-conjugating enzyme E2C | NM_001037449 | -5.9 | 6.9 | 9.5 | 2.0E-06 |
| *FREM1* | FRAS1 related extracellular matrix 1 | NM_001192995 | -5.9 | 3.2 | 5.8 | 2.4E-08 |
| *OAZ2* | ornithine decarboxylase antizyme 2 | NM_001172576 | -5.9 | 5.3 | 7.9 | 5.0E-15 |
| *COL11A1* | collagen, type XI, alpha 1 | NM_001166509 | -5.9 | 5.2 | 7.8 | 7.8E-07 |
| *CLEC3B* | C-type lectin domain family 3, member B | NM_001046212 | -5.9 | 7.4 | 10.0 | 1.5E-12 |
| *LOC100851833 /// SKA1* | spindle and kinetochore-associated protein 1-like /// spindle and kinetochore associate | NM_001075327 /// XM_003584991 | -6.0 | 4.6 | 7.2 | 2.1E-06 |
| *STAB1* | stabilin 1 | NM_001205570 | -6.0 | 4.3 | 6.9 | 1.2E-15 |
| *MFSD2A* | major facilitator superfamily domain containing 2A | NM_001101959 | -6.0 | 4.4 | 6.9 | 8.3E-11 |
| *H19* | H19, imprinted maternally expressed transcript (non-protein coding) | NR_003958 | -6.0 | 7.2 | 9.8 | 6.6E-09 |
| *LOC100137759* | N-acetyl-beta-glucosaminyl-glycoprotein 4-beta-N-acetylgalactosaminyltransferase 1-like | XM_003584770 /// XM_003588069 | -6.2 | 3.1 | 5.8 | 3.5E-13 |
| *FAM101B* | family with sequence similarity 101, member B | XM_002695711 /// XM_870793 | -6.2 | 7.0 | 9.6 | 9.6E-12 |
| *RASSF3* | Ras association (RalGDS/AF-6) domain family member 3 | NM_001192886 | -6.3 | 5.4 | 8.1 | 1.0E-15 |
| *EMCN* | endomucin | NM_001076420 | -6.3 | 5.8 | 8.5 | 3.2E-12 |
| *APOLD1* | apolipoprotein L domain containing 1 | NM_001101180 | -6.3 | 4.0 | 6.6 | 3.6E-11 |
| *SEP4* | septin 4 | NM_001034651 | -6.3 | 5.4 | 8.1 | 2.4E-12 |
| *COL15A1* | collagen, type XV, alpha 1 | NM_001191285 | -6.4 | 8.7 | 11.3 | 1.6E-12 |
| *CENPF* | centromere protein F, 350/400kDa (mitosin) | NM_001256586 /// XM_002694283 /// XM_612376 | -6.4 | 6.4 | 9.1 | 9.0E-08 |
| *MALL* | mal, T-cell differentiation protein-like | NM_001046115 | -6.4 | 3.0 | 5.7 | 9.3E-13 |
| *SLCO2B1* | solute carrier organic anion transporter family, member 2B1 | NM_174843 | -6.5 | 3.7 | 6.4 | 7.2E-11 |
| *MATN2* | matrilin 2 | NM_001102528 | -6.5 | 8.6 | 11.3 | 1.6E-14 |
| *LOC100847497* | uncharacterized LOC100847497 | XM_003584676 /// XM_003588086 | -6.6 | 7.7 | 10.4 | 6.6E-10 |
| *IGFBP6* | insulin-like growth factor binding protein 6 | NM_001040495 | -6.6 | 8.0 | 10.7 | 3.3E-12 |
| *SLCO2A1* | solute carrier organic anion transporter family, member 2A1 | NM_174829 | -6.6 | 3.5 | 6.2 | 4.8E-11 |

| | | | | | | |
|---|---|---|---|---|---|---|
| *TPBG* | trophoblast glycoprotein | XM_002690047 /// XM_593502 | -6.6 | 6.2 | 8.9 | 2.2E-14 |
| *ADAMDEC1 /// LOC100847471* | ADAM-like, decysin 1 /// ADAM DEC1-like | NM_001206371 /// XM_002689785 /// XM_003582556 /// XM_003586413 /// XM_582254 | -6.7 | 5.3 | 8.0 | 8.2E-08 |
| *C10H5orf13* | chromosome 10 open reading frame, human C5orf13 | NM_001105045 | -6.7 | 4.0 | 6.8 | 3.0E-08 |
| *ADAMDEC1* | ADAM-like, decysin 1 | NM_001206371 /// XM_002689785 /// XM_582254 | -6.7 | 7.0 | 9.8 | 1.6E-09 |
| *COL4A4* | collagen, type IV, alpha 4 | XM_002685639 /// XM_002703716 /// XM_003581872 /// XM_003585388 | -6.8 | 5.3 | 8.1 | 9.7E-11 |
| *KCNJ8* | potassium inwardly-rectifying channel, subfamily J, member 8 | NM_001040482 | -6.9 | 3.5 | 6.3 | 9.5E-12 |
| *HES1* | hairy and enhancer of split 1, (Drosophila) | NM_001034678 | -6.9 | 4.2 | 7.0 | 4.4E-11 |
| *TNRC6C* | trinucleotide repeat containing 6C | XM_002696160 /// XM_580298 | -6.9 | 3.3 | 6.0 | 6.5E-13 |
| *ASS1* | argininosuccinate synthase 1 | NM_173892 | -7.0 | 5.0 | 7.8 | 2.8E-12 |
| *ARHGEF25* | Rho guanine nucleotide exchange factor (GEF) 25 | NM_001024481 | -7.1 | 3.7 | 6.5 | 4.3E-14 |
| *LMO2* | LIM domain only 2 (rhombotin-like 1) | NM_001076352 | -7.1 | 4.7 | 7.5 | 2.5E-12 |
| *ATP6V1B2* | ATPase, H+ transporting, lysosomal 56/58kDa, V1 subunit B2 | NM_001001146 /// NM_176671 | -7.2 | 4.4 | 7.2 | 7.7E-15 |
| *C1QTNF7* | C1q and tumor necrosis factor related protein 7 | NM_001076201 | -7.2 | 3.6 | 6.5 | 1.4E-09 |
| *DENND2A* | DENN/MADD domain containing 2A | XM_002687070 /// XM_590179 | -7.2 | 3.7 | 6.6 | 3.4E-14 |
| *LDB2* | LIM domain binding 2 | NM_001046611 | -7.3 | 4.9 | 7.8 | 3.9E-15 |
| *IGF2* | insulin-like growth factor 2 (somatomedin A) | NM_174087 | -7.4 | 8.1 | 11.0 | 1.2E-12 |
| *AS3MT /// AS3MT* | arsenic (+3 oxidation state) methyltransferase /// arsenic (+3 oxidation state) methylt | NM_001035023 /// XM_001255467 | -7.5 | 5.6 | 8.5 | 1.1E-08 |
| *C1QC* | complement component 1, q subcomponent, C chain | NM_001206396 | -7.5 | 3.5 | 6.4 | 7.4E-11 |
| *TFF2* | trefoil factor 2 | NM_001083521 | -7.5 | 4.1 | 7.0 | 9.6E-10 |

| | | | | | | |
|---|---|---|---|---|---|---|
| *CDKN1C* | cyclin-dependent kinase inhibitor 1C (p57, Kip2) | NM_001077903 | -7.6 | 6.3 | 9.2 | 3.8E-09 |
| *RBM3* | RNA binding motif (RNP1, RRM) protein 3 | NM_001034363 | -7.9 | 6.1 | 9.0 | 8.7E-10 |
| *HFM1* | HFM1, ATP-dependent DNA helicase homolog (S. cerevisiae) | NM_001205576 | -8.0 | 2.9 | 5.9 | 5.7E-13 |
| *FAM64A* | family with sequence similarity 64, member A | NM_001099109 | -8.2 | 3.9 | 6.9 | 2.1E-07 |
| *PLXND1* | plexin D1 | XM_001789172 /// XM_002697122 | -8.2 | 5.7 | 8.8 | 7.4E-15 |
| *TM4SF18* | transmembrane 4 L six family member 18 | NM_001034287 /// NM_001184724 | -8.4 | 5.3 | 8.4 | 2.1E-10 |
| *MEOX2* | mesenchyme homeobox 2 | NM_001098045 | -8.4 | 2.8 | 5.9 | 1.8E-07 |
| *GANAB* | glucosidase, alpha; neutral AB | NM_001205777 | -8.4 | 5.6 | 8.7 | 3.1E-13 |
| *AFAP1L1* | actin filament associated protein 1-like 1 | NM_001100334 | -8.5 | 3.2 | 6.2 | 1.1E-14 |
| *ISLR* | immunoglobulin superfamily containing leucine-rich repeat | NM_001080729 | -8.6 | 3.9 | 7.0 | 2.0E-13 |
| *AKAP8L* | A kinase (PRKA) anchor protein 8-like | XM_002688560 /// XM_864732 | -8.6 | 4.5 | 7.6 | 9.0E-14 |
| *HEYL* | hairy/enhancer-of-split related with YRPW motif-like | NM_001024565 | -9.1 | 5.2 | 8.4 | 1.5E-13 |
| *COL13A1* | collagen, type XIII, alpha 1 | NM_001105433 | -9.6 | 3.5 | 6.8 | 2.8E-12 |
| *TCF21* | transcription factor 21 | NM_001014899 | -9.8 | 6.9 | 10.2 | 1.0E-11 |
| *CD34* | CD34 molecule | NM_174009 | -9.8 | 4.2 | 7.5 | 2.7E-14 |
| *NALCN* | sodium leak channel, non-selective | XM_002691980 /// XM_616223 | -9.9 | 3.6 | 6.9 | 7.0E-13 |
| *C1QA* | complement component 1, q subcomponent, A chain | NM_001014945 | -9.9 | 4.9 | 8.2 | 1.2E-11 |
| *PABPN1* | poly(A) binding protein, nuclear 1 | NM_174569 | -10.0 | 5.3 | 8.6 | 1.0E-15 |
| *CCL14* | chemokine (C-C motif) ligand 14 | NM_001046585 | -10.1 | 4.0 | 7.3 | 6.6E-09 |
| *PTH1R* | parathyroid hormone 1 receptor | NM_001075332 | -10.4 | 4.1 | 7.5 | 1.7E-13 |
| *PRMT1* | protein arginine methyltransferase 1 | NM_001015624 | -10.5 | 6.5 | 9.9 | 7.7E-15 |
| *LMCD1* | LIM and cysteine-rich domains 1 | NM_001076222 | -10.7 | 4.4 | 7.8 | 5.7E-14 |
| *APLNR* | apelin receptor | NM_001102524 | -11.0 | 3.4 | 6.9 | 1.4E-12 |
| *EGFLAM /// LOC100847583* | EGF-like, fibronectin type III and laminin G domains /// pikachurin-like | NM_001083478 /// XM_003587531 | -11.4 | 7.1 | 10.6 | 5.9E-11 |
| *RNASE6* | ribonuclease, RNase A family, k6 | NM_174594 | -11.4 | 3.5 | 7.0 | 2.5E-12 |
| *AMH* | anti-Mullerian hormone | NM_173890 | -11.5 | 3.6 | 7.1 | 1.6E-16 |

| | | | | | | |
|---|---|---|---|---|---|---|
| *COL16A1 /// LOC100849968* | collagen, type XVI, alpha 1 /// collagen alpha-1(XVI) chain-like | XM_002685618 /// XM_003585098 /// XM_583549 | -11.8 | 7.1 | 10.7 | 3.2E-11 |
| *FRZB* | frizzled-related protein | NM_174059 | -11.8 | 3.2 | 6.8 | 1.2E-12 |
| *LAMA2 /// LOC100848461* | laminin, alpha 2 /// laminin subunit alpha-2-like | XM_001787958 /// XM_002690220 /// XM_003585167 /// XM_003585378 /// XM_003586475 | -11.9 | 5.1 | 8.6 | 6.3E-12 |
| *LRRC70* | leucine rich repeat containing 70 | XM_002696307 /// XM_002702499 | -12.0 | 4.1 | 7.6 | 8.4E-15 |
| *RGS5* | regulator of G-protein signaling 5 | NM_001034707 | -12.2 | 5.3 | 8.9 | 9.6E-12 |
| *TMEM88* | transmembrane protein 88 | NM_001098378 | -12.3 | 4.2 | 7.9 | 2.0E-13 |
| *CLEC14A* | C-type lectin domain family 14, member A | NM_001077890 | -12.4 | 3.9 | 7.5 | 1.0E-15 |
| *PLIN5* | perilipin 5 | NM_001101136 | -12.5 | 2.9 | 6.6 | 4.3E-14 |
| *ALDH1A2* | aldehyde dehydrogenase 1 family, member A2 | XM_002690855 /// XM_615062 | -13.2 | 4.2 | 7.9 | 7.7E-15 |
| *ASPN* | asporin | NM_001034309 | -13.6 | 6.4 | 10.2 | 7.5E-11 |
| *SDPR* | serum deprivation response | XM_002685467 /// XM_610845 | -13.7 | 5.2 | 9.0 | 1.3E-14 |
| *HSD17B1 /// HSD17B1* | hydroxysteroid (17-beta) dehydrogenase 1 /// hydroxysteroid (17-beta) dehydrogenase 1-l | NM_001102365 /// XM_001253407 | -15.5 | 5.0 | 8.9 | 7.5E-11 |
| *HPGD* | hydroxyprostaglandin dehydrogenase 15-(NAD) | NM_001034419 | -16.0 | 6.2 | 10.2 | 6.7E-11 |
| *GPC3* | glypican 3 | NM_001035463 | -16.6 | 5.6 | 9.7 | 9.0E-14 |
| *BRB* | brain ribonuclease | NM_173891 | -17.4 | 2.6 | 6.8 | 1.6E-12 |
| *DHRS3* | dehydrogenase/reductase (SDR family) member 3 | NM_174180 | -17.7 | 5.9 | 10.0 | 5.0E-13 |
| *HMGB3* | high mobility group box 3 | NM_001076285 /// NM_001113257 | -17.7 | 5.4 | 9.5 | 1.3E-13 |
| *AQP1* | aquaporin 1 (Colton blood group) | NM_174702 | -17.9 | 3.4 | 7.6 | 4.6E-11 |
| *COLEC11* | collectin sub-family member 11 | NM_001076303 | -20.3 | 3.8 | 8.1 | 6.6E-11 |
| *C11H2orf40* | chromosome 11 open reading frame, human C2orf40 | NM_001038113 | -20.8 | 3.7 | 8.1 | 3.2E-10 |
| *DUSP12* | dual specificity phosphatase 12 | XM_002685847 /// XM_581568 | -26.0 | 5.4 | 10.1 | 5.8E-14 |
| *SHISA2* | shisa homolog 2 (Xenopus laevis) | NM_001101265 | -57.2 | 3.9 | 9.7 | 2.4E-14 |

| | | | | | | |
|---|---|---|---|---|---|---|
| *CXCL14* | chemokine (C-X-C motif) ligand 14 | NM_001034410 | -76.5 | 4.5 | 10.7 | 1.6E-16 |

*> 4-fold change, FDR P < 0.05*
